# Spatial transcriptomics delineates potential differences in intestinal phenotypes of cardiac and classical necrotizing enterocolitis

**DOI:** 10.1016/j.isci.2025.112166

**Published:** 2025-03-07

**Authors:** Kathryn Y. Burge, Constantin Georgescu, Hua Zhong, Adam P. Wilson, Aarthi Gunasekaran, Zhongxin Yu, Addison Franca, Jeffrey V. Eckert, Jonathan D. Wren, Hala Chaaban

**Affiliations:** 1 Department of Pediatrics, Section of Neonatal-Perinatal Medicine, University of Oklahoma Health Sciences Center, Oklahoma City, OK 73104, USA; 2 Genes and Human Disease Research Program, Oklahoma Medical Research Foundation, Oklahoma City, OK 73104, USA; 3 Department of Pathology, University of Oklahoma Health Sciences Center, Oklahoma City, OK 73104, USA

**Keywords:** Gastroenterology, Pediatrics, Disease, Components of the immune system, Proteomics, Transcriptomics

## Abstract

Necrotizing enterocolitis (NEC) is a devastating neonatal gastrointestinal disease, often resulting in multi-organ failure and death. While classical NEC is strictly associated with prematurity, cardiac NEC is a subset of the disease occurring in infants with comorbid congenital heart disease. Despite similar symptomatology, the NEC subtypes vary slightly in presentation and may represent etiologically distinct diseases. We compared ileal spatial transcriptomes of patients with cardiac and classical NEC. Epithelial and immune cells cluster well by cell-type segment and NEC subtype. Differences in metabolism and immune cell activation functionally differentiate the cell-type makeup of the NEC subtypes. The classical NEC phenotype is defined by dysbiosis-induced inflammatory signaling and metabolic acidosis, while that of cardiac NEC involves reduced angiogenesis and endoplasmic reticulum stress-induced apoptosis. Despite subtype-associated clinical and demographic variability, spatial transcriptomics has substantiated pathway and network differences within immune and epithelial segments between cardiac and classical NEC.

## Highlights

• Cardiac and classical necrotizing enterocolitis cause similar symptoms in neonates
• Spatial transcriptomics hints at differing etiologies for these two NEC subtypes
• ER stress-induced apoptosis and reduced angiogenesis characterize cardiac NEC
• Dysbiosis-induced inflammation and metabolic acidosis characterize classical NEC

## Introduction

Necrotizing enterocolitis (NEC) is the most common gastrointestinal emergency in the neonatal intensive care unit (NICU), affecting 7% of very low birthweight (VLBW; <1,500 g) and 10% of extremely LBW (ELBW; <1,000 g) infants.^1^ Though the etiology is not clearly understood, a hyperinflammatory, immature immune system, and a functionally and structurally underdeveloped intestine compound prematurity-associated dysbiosis to allow for bacterial translocation of the intestinal barrier, necrosis of the bowel, multisystem organ failure,^2^ and mortality approaching 30% in VLBW infants.^3^

The multifactorial and heterogeneous pathophysiology of the disease has resulted in the belief that several subsets, still poorly defined, exist within the umbrella of “NEC”, potentially with unique etiologies. Classical NEC^4^^,^^5^ is predominantly associated with preterm (<37 weeks gestational age) infants, often initiates with feeding intolerance and abdominal distention, and may progress with alarming rapidity to bowel perforation or sepsis. Transfusion-associated NEC (TANEC) temporally relates packed red blood cell transfusions with development of the disease,^6^^,^^7^ but recent studies have suggested severe anemia contributes more substantially to NEC risk.^8^ Atypical NEC presents at earlier time points in preterm and term infants, generally within the first week postpartum.^9^^,^^10^ Finally, a commonly recognized risk factor for NEC development in both preterm^11^ and term^12^ neonates is the presence of congenital heart disease (CHD), particularly ductal-dependent hypoplastic left heart syndrome (HLHS).^13^^,^^14^^,^^15^ In normal weight (>2,500 g) infants born term or near-term, CHD is associated with 20% of NEC cases.^16^

While classical and CHD-associated (cardiac) NEC share nearly identical clinical symptomatology and progression, cardiac NEC is increasingly viewed as a distinct disease.^17^^,^^18^^,^^19^^,^^20^ The prevailing hypothesis of cardiac NEC pathophysiology is predicated upon a primary etiology of CHD-associated reductions in splanchnic perfusion, resulting in intestinal ischemia and localized hypoxia.^21^ Subtle differences in cardiac and classical NEC presentation and clinical outcomes may indicate differential pathogenesis at a cellular level. In addition to affecting infants of higher birthweight and gestational age,^22^ infants with cardiac NEC typically develop the disease at an earlier postnatal age than infants with classical NEC.^23^ The terminal ileum is a significant site of injury in both NEC subtypes,^24^^,^^25^^,^^26^ but due to variability in blood supply and associated ischemic risk, the colon is commonly also affected in cardiac NEC.^18^^,^^27^^,^^28^ While surgical resection of the bowel is less often indicated in cardiac compared with classical NEC, infants with cardiac NEC, as a consequence of CHD, generally suffer higher all-cause mortality,^17^ though mortality specifically attributable to vascular versus intestinal components is difficult to estimate.^29^

Advances in biological understanding of classical NEC have occurred in recent decades, but the rarity (3–5% incidence^13^^,^^17^^,^^30^) of cardiac NEC has deemed the latter disease significantly understudied. Studies of cardiac NEC, to date, have been largely descriptive.^18^^,^^20^ Recent work has highlighted clinical and histological differences between the NEC subtypes, indicating cardiac NEC is defined by decreased blood gas pH, increased leukocyte and neutrophil concentrations, and increased neutrophil activation (neutrophil elastase and citrullinated H3) compared with classical NEC.^20^ However, transcriptional differences, particularly while preserving spatial tissue architecture, have never been evaluated in either disease. Here, we analyzed spatial transcriptomes derived from the terminal ileum of infants diagnosed with cardiac and classical NEC, highlighting potential pathophysiological mechanisms for future mechanistic study, driven by differential gene expression in two cell-type compartments thought to dictate the respective phenotypes, intestinal immune cells and epithelium.

## Results

### Cardiac and classical NEC appear transcriptionally distinct

To evaluate differences in intestinal pathology associated with cardiac and classical NEC, we utilized surgically resected, formalin-fixed, paraffin-embedded (FFPE) samples of the terminal ileum from patients histologically diagnosed with cardiac or classical NEC (Table S1). We utilized NanoString GeoMx digital spatial profiling (DSP) to sequence the whole transcriptome of specific immune- and epithelial-rich regions of interest (ROIs). ROIs from each patient tissue were selected through a combination of hematoxylin and eosin (H&E)-aided histopathological analysis and immunofluorescent cell-type segmentation (Figure S1; STAR Methods).

**Figure 1:**
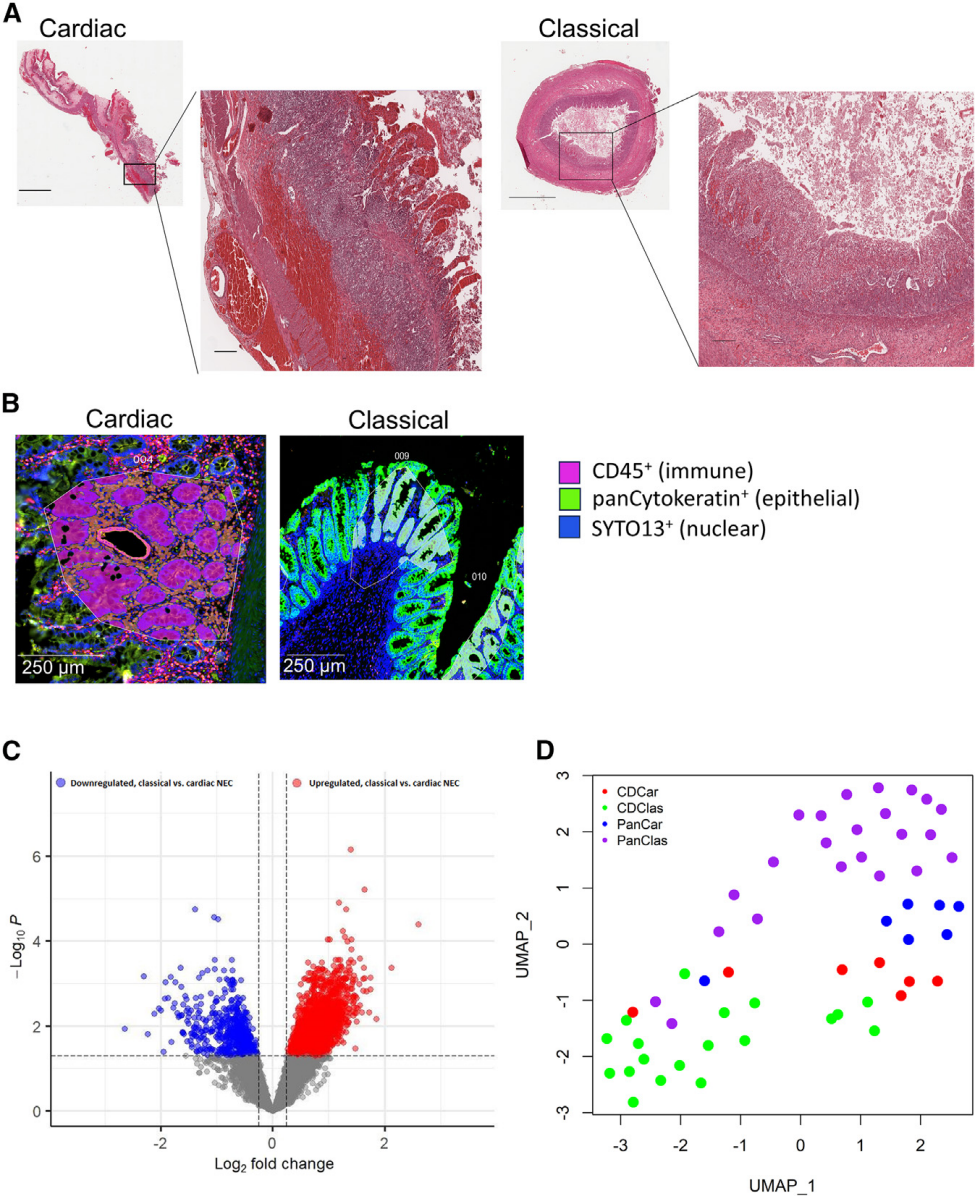
Cardiac and classical NEC appear transcriptionally distinct (A) Representative H&E histology of cardiac (left; diffuse ischemic changes and areas of hemorrhagic necrosis) and classical (right; transmural necrosis and marked inflammatory infiltrates) NEC samples. Magnification: 4× (left), 20× (right); Scale bar: 2 mm (left), 200 μm (right). (B) Representative immunofluorescent staining (scale bars: 250 μm) and ROI masking of CD45 (magenta), panCK (green), and nuclei (blue) in representative cardiac CD45^+^ ROI (left) and classical PanCK^+^ ROI (right) NEC sections. (C) Volcano plot demonstrating upregulated (red) or downregulated (blue) DEGs in classical (*n* = 5) compared to cardiac (*n* = 2) NEC (FDR-adjusted *p* < 0.05 and log_2_ fold change ≥0.25). (D) UMAP 2D visualization of ROI transcriptional signatures differentiated by NEC subtype and cell-type segment. Red: cardiac NEC immune; green: classical NEC immune; blue: cardiac NEC epithelial; purple: classical NEC epithelial. NEC, necrotizing enterocolitis; H&E, hematoxylin and eosin; ROI, region of interest; panCK, pancytokeratin; FDR, false discovery rate; DEGs, differentially expressed genes; UMAP, uniform manifold approximation and projection. Also see Table S1 and Figures S1–S6.

Cardiac and classical NEC patients displayed typical clinical signs of NEC and acknowledged differences among the NEC subtypes, high-normal neutrophil percentage and normal platelet counts in the former and neutropenia and moderate or severe thrombocytopenia in the latter (Table S1), were demonstrated in this small patient cohort. However, blinded histopathologist analysis of resected tissues, characterized by ischemic necrosis, acute inflammation, and in some cases, focal perforation, could not differentiate between the two conditions (Figures 1A and S2). Given critical roles of intestinal epithelial and immune cells in NEC pathogenesis,^31^ we morphologically stained patient tissue sections for intestinal epithelial (pan-cytokeratin^+^ or panCK^+^) and immune (CD45^+^) cell types using immunofluorescent antibodies (Figures 1B and Figures S3–S6). The transcriptomes within these ROIs were subsequently sequenced according to cell-type segment and NEC subtype, resulting in 18,677 probes sequenced in a total of 56 ROIs. Despite the popular adage that NEC represents convergence of unique, heterogeneous etiologies on a final common pathway,^32^ cardiac and classical NEC epithelial and immune cells appear transcriptionally distinct even at this late stage of disease (Figure 1C). Further, dimensionality reduction of the transcriptomes of all patient ROIs using uniform manifold approximation and projection (UMAP, Figure 1D) indicates individual ROIs cluster well by both cell-type segment and NEC subtype, a surprising finding given the recognized heterogeneity in NEC etiology.^33^^,^^34^

### Patterns of immune cell activation and regulation of cellular metabolism differentiate cardiac and classical NEC cell-type clusters

To evaluate differences in intestinal cell-type abundances in cardiac and classical NEC, cell counts for 9 (main; Figure S7A) and 93 (granular; Figure S8A) distinct cell types were estimated using cellular deconvolution and gene expression profiles derived from the Human Cell Atlas (HCA) adult gut single-cell RNA sequencing (scRNA-seq) reference dataset.^35^ Cardiac NEC ROIs were defined by enriched B cells (granular: pre-B cells) and mesenchymal cells (granular: follicular dendritic cells, interstitial cells of Cajal, and cycling stromal cells), and reduced *CA2* (carbonic anhydrase 2)-, *SLC26A2* (solute carrier family 26 member 2)-, and *FABP1* (fatty acid-binding protein 1)-expressing epithelial cells compared with classical NEC ROIs (Figures S7B–S7D, S8B and S8C). In addition, while not significantly inducing differences in the main cell-type category, endothelial (*p* = 0.11), granular cell-type characterization indicated cardiac NEC ROIs were enriched in mature venous endothelial and fetal arterial endothelial cells compared with classical NEC ROIs.

**Figure 2:**
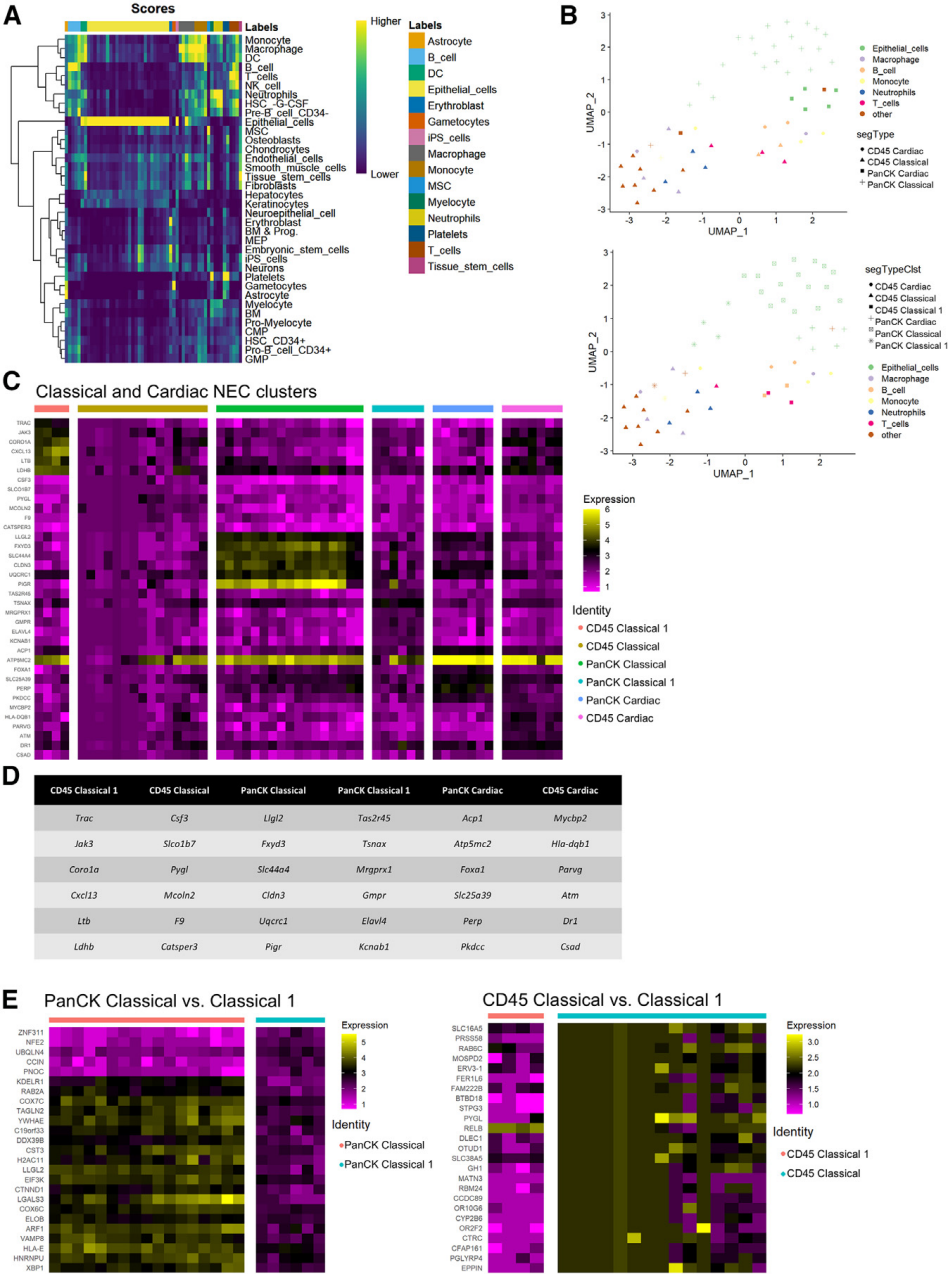
Cell deconvolution and ROI clustering based on cell-type abundances (A) Estimated cell-type composition per ROI (HPCA reference; see STAR Methods). Each column shows one ROI estimated cell-type distribution. The cell type corresponding with the highest expression intensity was designated the ROI cell-type identity and denoted by column label at top of heatmap. (B) UMAP representation of Seurat guided clustering with initial 4 NEC subtype and cell-type segments (top) and eventual 6 suggested clusters (bottom). (C) Heatmap showing expression profile variation across all ROIs for top 6 marker genes characterizing each of the six NEC subtype and cell-type clusters. Cluster identities are specified by column blocks and top heatmap labels. (D) Table of marker genes for 6 clusters illustrated in heatmap (C). (E) Expression profile of marker genes differentiating PanCK+ (left) and CD45^+^ (right) clusters within classical NEC. ROI, region of interest; HPCA, Human Primary Cell Atlas; DC, dendritic cells; iPS, inducible pluripotent stem cells; MSC, mesenchymal stem cells; UMAP, uniform manifold approximation and projection; PanCK, pancytokeratin; NEC, necrotizing enterocolitis. Also see Figures S7–S8.

To explore further heterogeneity within cell-type abundance patterns in cardiac and classical NEC, we performed cell deconvolution using the Human Primary Cell Atlas (HPCA) reference dataset^36^ (Figure 2A). After assigning a primary cell type to each ROI, guided clustering in Seurat (Figure 2B) delineated marker genes for each cluster (Figures 2C and 2D). While cardiac CD45^+^ and PanCK^+^ ROIs segregated well, the multifactorial nature of classical NEC^37^ resulted in more heterogenous clustering within CD45^+^ and PanCK^+^ ROIs. Cardiac NEC CD45^+^ ROIs were characterized by marker genes associated with DNA damage response and induction of transforming growth factor β (TGF-β) signaling (*MYCBP2* [MYC binding protein 2], *ATM* [ataxia telangiectasia mutated]),^38^^,^^39^ antigen presentation (*HLA-DQB1*, major histocompatibility complex, class II, DQ beta 1),^40^ and regulation of blood cell homeostasis (*PARVG*, gamma-parvin),^41^ processes largely driven by B and dendritic cells estimated (Figures S7B and S8C) to be upregulated in cardiac NEC ROIs. Consistent with acknowledged roles in NEC pathogenesis,^31^ T cell inflammatory programming and chemotaxis (*TRAC* [T cell receptor alpha constant], *CORO1A* [coronin 1A], *CXCL13* [CXC motif chemokine ligand 13], *LDHB* [lactate dehydrogenase B], *LTB* [lymphotoxin beta], *JAK3* [janus kinase 3])^42^^,^^43^^,^^44^^,^^45^^,^^46^^,^^47^ and inflammatory macrophage signaling (*PYGL* [glycogen phosphorylase L], *CSF3* [colony stimulating factor 3], *MCOLN2* [mucolipin TRP cation channel 2])^48^^,^^49^^,^^50^ characterized respective clusters of classical NEC CD45^+^ ROIs. Cardiac NEC PanCK^+^ ROIs were characterized by marker genes associated with mitochondrial function (*SLC25A39*, *ATP5MC2* [ATP synthase membrane subunit c locus 2])^51^^,^^52^ and regulation of apoptosis (*PERP* [p53 apoptosis effector related to PMP22], *ACP1* [acid phosphatase 1], *FOXA1* [forkhead box A1]).^53^^,^^54^^,^^55^ Classical NEC PanCK^+^ ROI clusters were characterized either by marker genes associated with oxidative phosphorylation (*UQCRC1* [ubiquinol-cytochrome c reductase core protein 1], *SLC44A4*, *FXYD3* [FXYD domain containing ion transport regulator 3])^56^^,^^57^^,^^58^ and regulation of epithelial-mesenchymal transition (*LLGL2* [LLGL scribble cell polarity complex component 2], *PIGR* [polymeric immunoglobulin receptor], *CLDN3* [claudin 3]),^59^^,^^60^^,^^61^ or those associated with G protein-coupled receptor (GPCR) signaling (*TAS2R45* [taste 2 receptor member 45], *MRGPRX1* [MAS related GPR family member X1])^62^^,^^63^ and neuronal function (*TSNAX* [translin associated factor X], *ELAVL4* [ELAV like RNA-binding protein 4], *KCNAB1* [potassium voltage-gated channel subfamily A regulatory beta subunit 1]).^64^^,^^65^^,^^66^ Additional genes differentiating CD45^+^ and PanCK^+^ clusters within classical NEC are shown in Figure 2E.

### Dopaminergic and vascular endothelial growth factor signaling characterize CD45^+^ transcriptional differences

**Figure 3:**
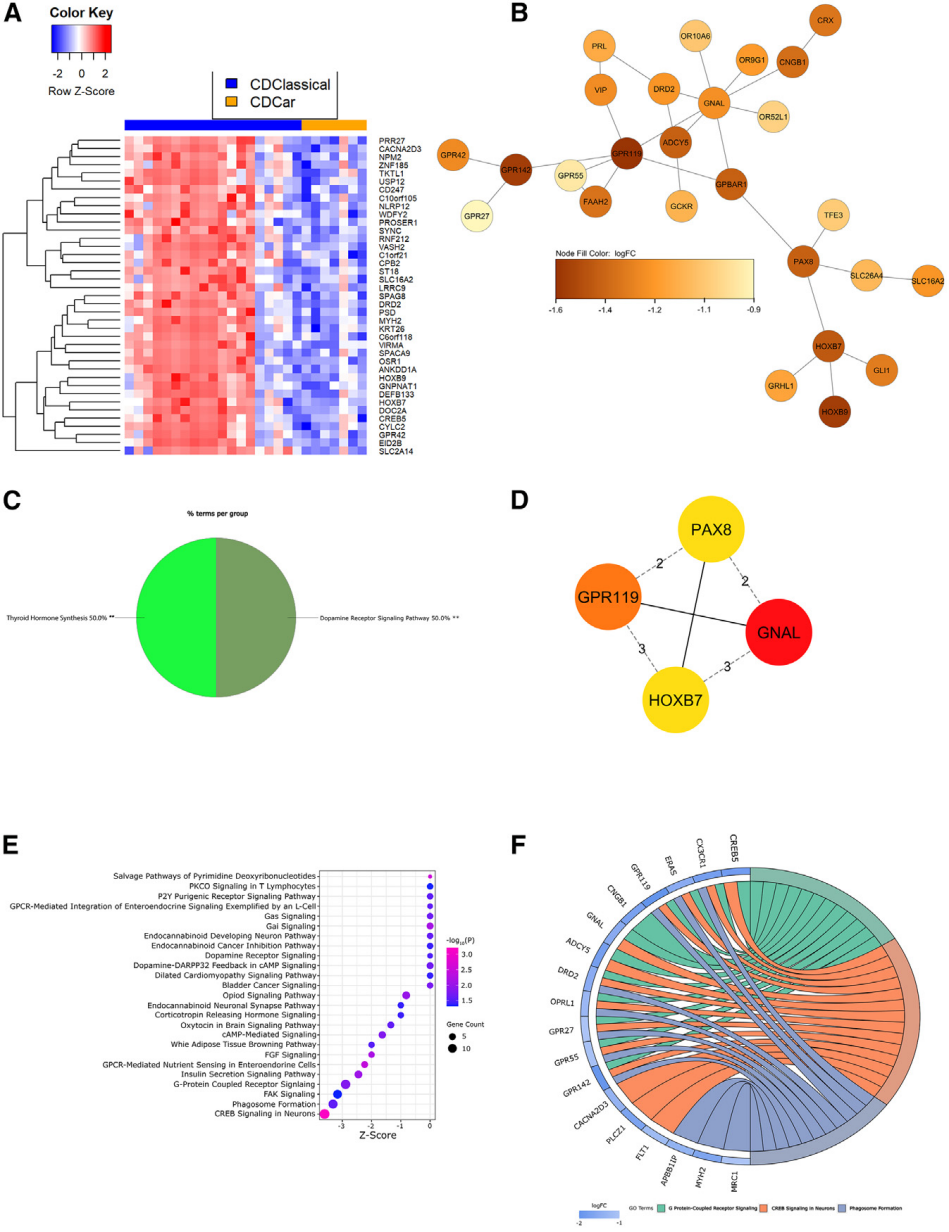
Dopaminergic and VEGF signaling characterize CD45^+^ transcriptional differences in cardiac and classical NEC (A) Heatmap of Z-scored normalized expression of the top 40 DEGs (FDR-adjusted *p* < 0.15). (B) Largest PPI subnetwork determined by STRING analysis in CytoScape. (C) ClueGO pie chart of GO term functional enrichment (% terms/group); ∗∗adjusted *p* < 0.01. (D) Top 4 hub genes identified through CytoHubba (red = strongest associations). (E) Bubble plot of IPA-defined differentially enriched pathways. Bubble size correlates with gene overlap (≥2), color indicates significance (-log_10_(*P*)) of pathway enrichment, and x axis represents *Z* score. (F) Chord diagram illustrating composition of 3 differentially regulated pathways (by *Z* score and gene count) annotated in (E). Pathways are shown on the right and gene fold change is indicated on the left. Chords connecting genes to pathways indicate gene inclusion within the pathway. VEGF, vascular endothelial growth factor; NEC, necrotizing enterocolitis; DEGs, differentially expressed genes; FDR, false discovery rate; PPI, protein-protein interaction; STRING, search tool for the retrieval of interacting genes/proteins; GO, gene ontology; IPA, ingenuity pathway analysis. Also see Figures S11A, S13, S19A, S20A–S20C, Table S4, Figures S23 and S24.

**Figure 4:**
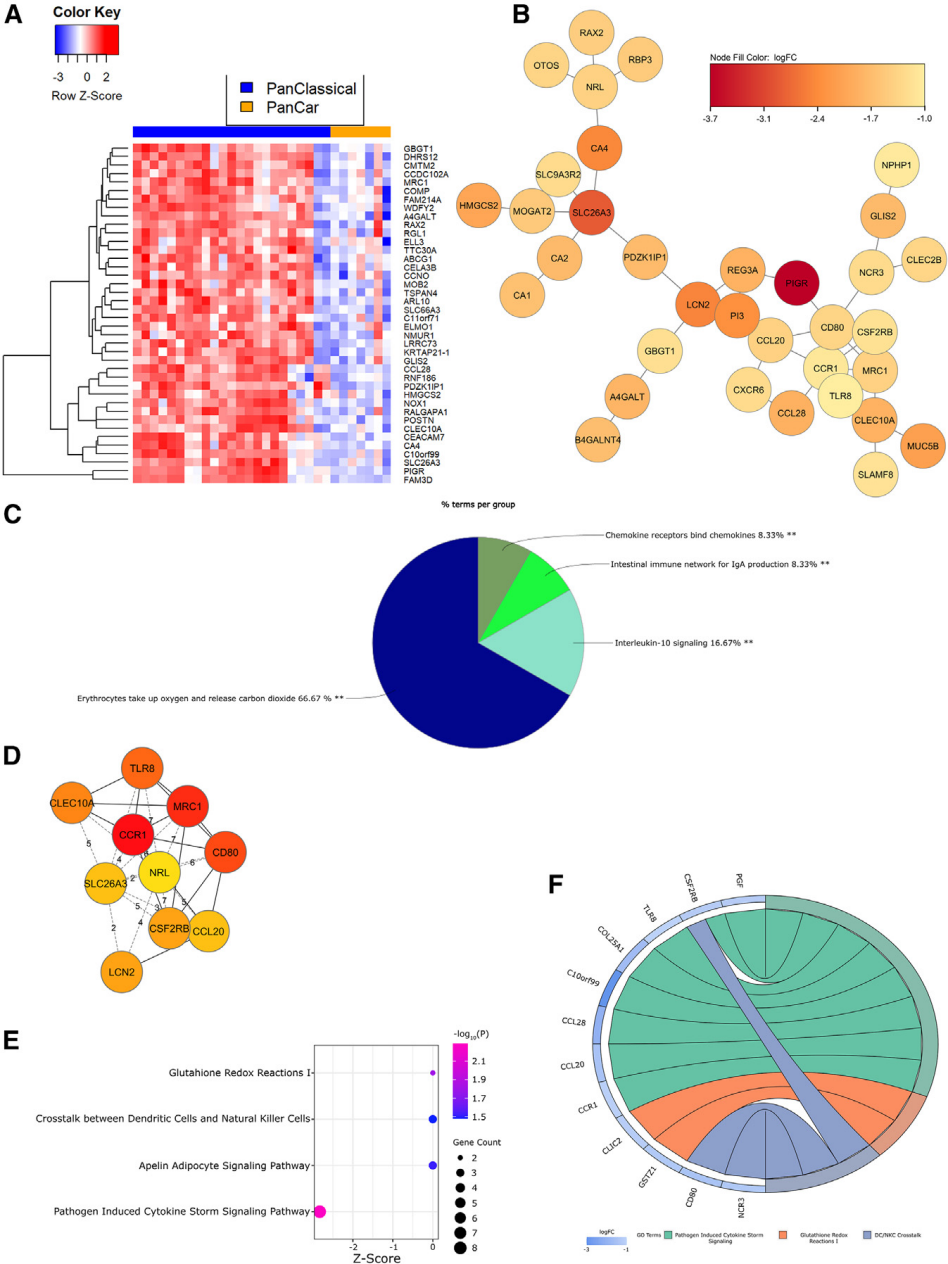
Classical NEC PanCK^+^ ROIs are defined by pathogen-induced cytokine storm signaling (A) Heatmap of *Z* scored normalized expression of the top 40 DEGs (FDR-adjusted *p* < 0.01) (B) Largest PPI subnetwork determined by STRING analysis in CytoScape. (C) ClueGO pie chart of GO term functional enrichment (% terms/group); ∗∗adjusted *p* < 0.01. (D) Top 10 hub genes identified through CytoHubba (red = most connections). (E) Bubble plot of IPA-defined differentially enriched pathways. Bubble size correlates with gene overlap (≥2), color indicates significance (-log_10_(*P*)) of pathway enrichment, and x axis represents *Z* score. (F) Chord diagram illustrating composition of 3 differentially regulated pathways (by *Z* score and gene count) annotated in (E). Pathways are shown on the right and gene fold change is indicated on the left. Chords connecting genes to pathways indicate gene inclusion within the pathway. NEC, necrotizing enterocolitis; PanCK, pancytokeratin; ROIs, regions of interest; DEGs, differentially expressed genes; FDR, false discovery rate; PPI, protein-protein interaction; STRING, search tool for the retrieval of interacting genes/proteins; GO, gene ontology; IPA, ingenuity pathway analysis. Also see Figures S11B, S14, S19B, S20D, Table S4, and Figures S5–S26.

**Figure 5:**
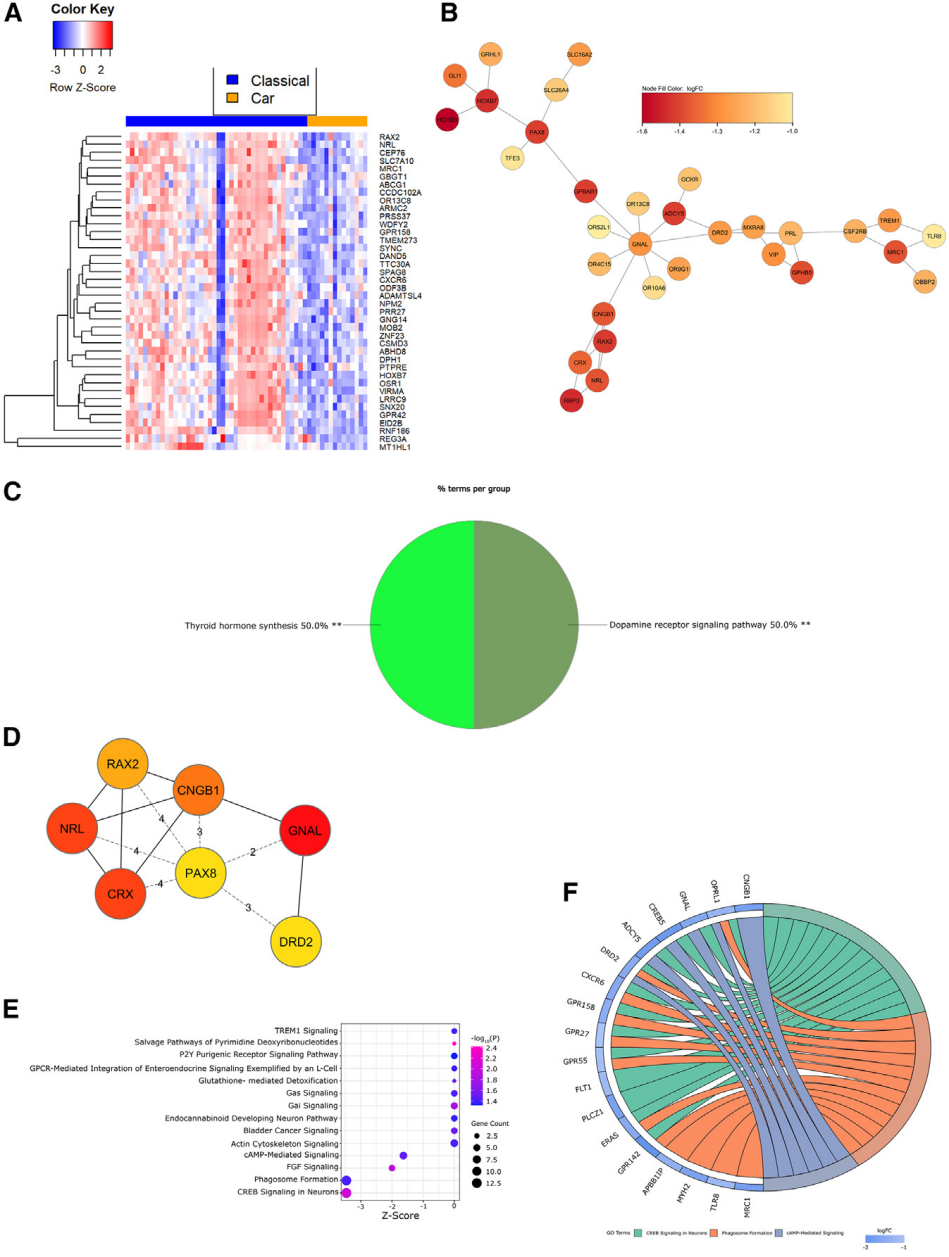
The cardiac NEC transcriptome reflects ER stress while a classical NEC phenotype of inflammatory mucosal acidosis is prominent in directionally concordant DEGs (A) Heatmap of *Z* scored normalized expression of the top 40 DEGs (FDR-adjusted *p* < 0.15). (B) Largest PPI subnetwork determined by STRING analysis in CytoScape. (C) ClueGO pie chart of GO term functional enrichment (% terms/group); ∗∗adjusted *p* < 0.01. (D) Top 7 hub genes identified through CytoHubba (red = most connections). (E) Bubble plot of IPA-defined differentially enriched pathways. Bubble size correlates with gene overlap (≥2), color indicates significance (-log_10_(*P*)) of pathway enrichment, and x axis represents *Z* score. (F) Chord diagram illustrating composition of 3 differentially regulated pathways (by *Z* score and gene count) annotated in (E). Pathways are shown on the right and gene fold change is indicated on the left. Chords connecting genes to pathways indicate gene inclusion within the pathway. NEC, necrotizing enterocolitis; DEG, differentially expressed gene; PanCK, pancytokeratin; ROIs, regions of interest; FDR, false discovery rate; PPI, protein-protein interaction; STRING, search tool for the retrieval of interacting genes/proteins; GO, gene ontology; IPA, ingenuity pathway analysis. Also see Figures S11C, S15, S19C, S21A, S21B, Table S4, Figures S27 and S28.

**Figure 6:**
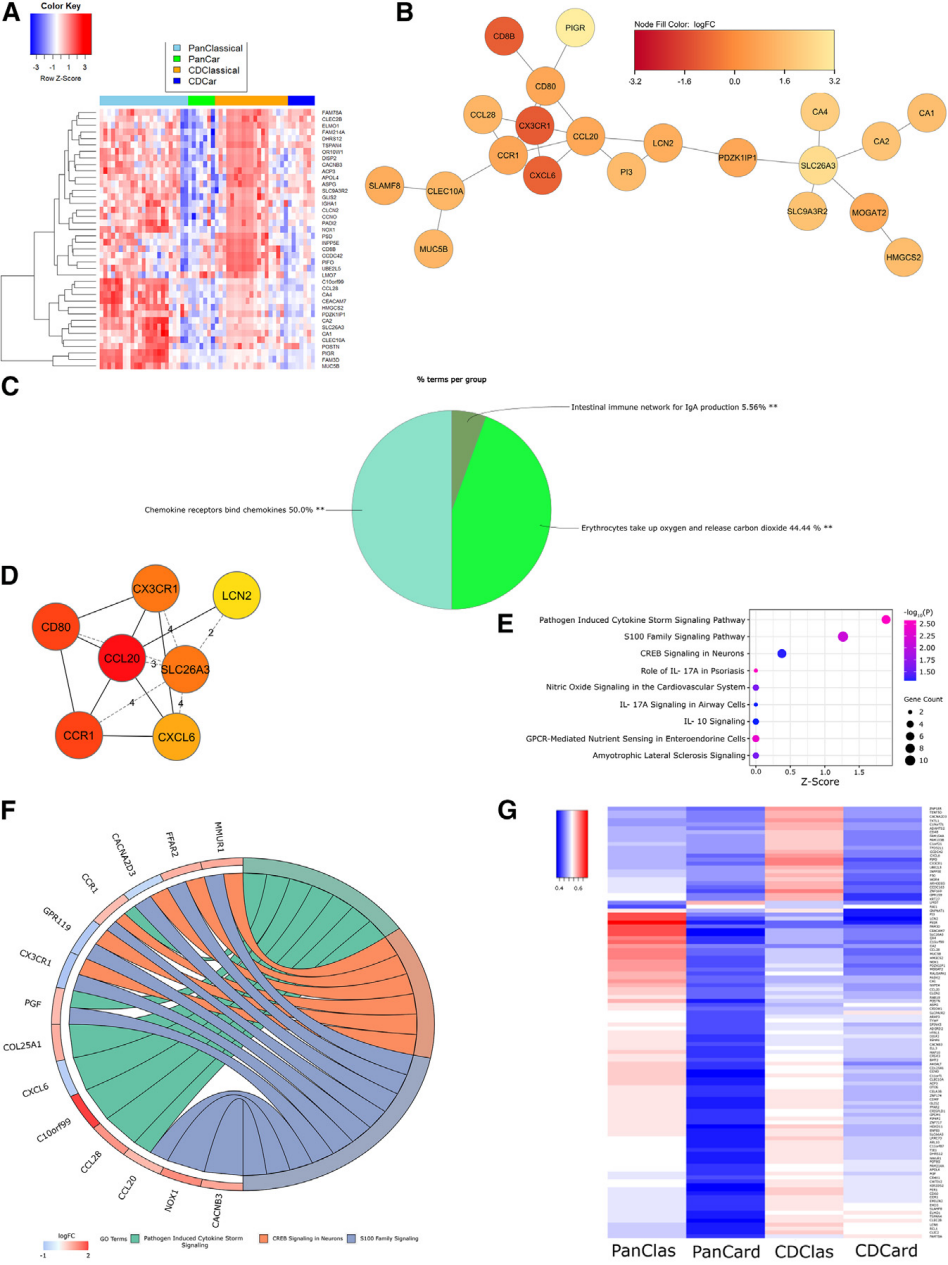
Gene interactions differentiate NEC subtypes via pathogen-induced cytokine storm signaling (A) Heatmap of *Z* scored normalized expression of the top 40 DEGs (FDR-adjusted *p* < 0.05). (B) Largest PPI subnetwork determined by STRING analysis in CytoScape. (C) ClueGO pie chart of GO term functional enrichment (% terms/group); ∗∗adjusted *p* < 0.01. (D) Top 7 hub genes identified through CytoHubba (red = most connections). (E) Bubble plot of IPA-defined differentially enriched pathways. Bubble size correlates with gene overlap (≥2), color indicates significance (-log_10_(*P*)) of pathway enrichment, and x axis represents *Z* score. (F) Chord diagram illustrating composition of 3 differentially regulated pathways (by *Z* score and gene count) annotated in (E). Pathways are shown on the right and gene fold change is indicated on the left. Chords connecting genes to pathways indicate gene inclusion within the pathway. (G) Heatmap of *Z* scored normalized expression of interacting DEGs (FDR-adjusted *p* < 0.05), averaged across cell-type segment and NEC subtype. NEC, necrotizing enterocolitis; DEGs, differentially expressed genes; FDR, false discovery rate; PPI, protein-protein interaction; STRING, search tool for the retrieval of interacting genes/proteins; GO, gene ontology; IPA, ingenuity pathway analysis. Also see Figures S11D, S16, S19D, S21D, S22, Table S4, Figures S29 and S30.

Segregating immune and epithelial transcriptomes in both NEC subtypes allowed for 6 comparative analyses: immune cell expression between NEC subtypes, epithelial cell expression between NEC subtypes, cell-type segment concordant gene expression between NEC subtypes, cell-type segment interaction gene expression between NEC subtypes, and epithelial and immune expression within the same NEC subtype (Figures S9, S10, S11E and S11F; false discovery rate [FDR]-adjusted thresholds in Table S2). Within sequencing analyses, cell-type abundances and differential regulation of transcriptional programs within cell types contribute to variability in gene expression patterns.^67^ Using reverse deconvolution, we compared the expression of each gene within each ROI predicted/fitted via computational cell mixing with that observed through cell-type abundance patterns. Genes with high residual standard deviation and low correlation between fitted and observed expression values (overlap of blue and red in Figure S12; see STAR Methods) are genes with highly variable expression that is poorly predicted by cell-type abundance within the ROI. Because these cell-type abundance-independent genes represent those with coordinated expression or regulation within respective cell-type (CD45^+^ or PanCK^+^) segments, we focus exclusively on analyses utilizing these differentially expressed genes (DEGs) within the main text (Figures 3, 4, 5, and 6). Analyses utilizing the comprehensive (cell-type abundance-independent and -dependent; **see** STAR Methods) suite of DEGs are found in Figures S13–S19.

When comparing gene expression within CD45^+^ ROIs of cardiac and classical NEC ileum, we utilized DEGs (Figures 3A and S11A) to construct a protein-protein interaction (PPI) network, where nodes indicate discrete genes and edges represent gene connections. The largest subnetwork (Figure 3B) was subjected to functional annotation, indicating classical NEC immune enrichment in dopamine and GPCR signaling pathways (Figure 3C). Hub gene analysis identified *GNAL* (G protein subunit alpha L), *GPR119* (GPCR 119), *PAX8* (paired box gene 8), and *HOXB7* (homeobox B7) as genes driving connections within the PPI network (Figure 3D). Genes overlapping with enriched pathways (Figures 3E and 3F) identified through ingenuity pathway analysis (IPA) further confirm dopaminergic signaling as a driver of immune transcriptional differences in the cardiac and classical NEC intestine. To visually explore spatial heterogeneity of gene expression, expression levels of select genes were overlayed on original tissue images using SpatialOmicsOverlay (Figures S20–S22). Finally, panels targeting nearly 80 proteins were assessed for a subset of NEC tissues (*n* = 2 cardiac, *n* = 2 classical; Table S3, see STAR Methods). Integration of the targeted proteome within CD45^+^ ROI transcriptomes revealed differential regulation of the Kyoto encyclopedia of genes and genomes (KEGG) pathways, viral protein interaction with cytokine and cytokine receptor and chemokine signaling (combined *p* values, 0.00485 and 0.01299, respectively; Table S4).

Reduced vascular endothelial growth factor (VEGF) expression is noted in both human and experimental NEC^68^^,^^69^ and is associated more broadly with intestinal ischemia.^70^ The hub gene, *HOXB7*, an inducer of VEGF expression,^71^ is downregulated in cardiac compared with classical NEC CD45^+^ ROIs. *HOXB7* is a transcription factor regulating bone marrow-derived mesenchymal progenitor proliferation and differentiation,^72^ activating basic fibroblast growth factor (bFGF) secretion,^73^ and inducing epithelial-mesenchymal transition (EMT)^74^ and epithelial restitution^75^ through binding of the epidermal growth factor receptor (EGFR).^76^ VEGF regulates angiogenic activities largely through expression of the dopamine D2 receptor (D2R).^77^ Intriguingly, both D2R (encoded by *DRD2*; Figure S20A), a member of the GPCR family and adenylate cyclase 5 (AC5, encoded by *ADCY5*; Figure S20B), an enzyme inhibited by D2R signaling,^78^ are common constituents of the functional pathways ascribed to this CD45^+^ comparison. The hub gene, *GNAL* (Figure S20C), encodes the G protein subunit, Gα_olf_, responsible for coupling dopamine receptor D1 (D1R) and the adenosine A_2A_ receptor (ADORA2A) to activate AC5 during dopaminergic signaling.^79^ Dopamine receptor signaling has variable effects across immune cell types, inducing chemokine release and cell migration in monocytes, release of neutrophil extracellular traps (NETs) in neutrophils, activation of nuclear factor kappa B (NF-κB) and bidirectional effects on phagocytosis in macrophages, and heterogeneous effects on cytokine production and differentiation within T cell subsets.^80^ Downregulated D2R signaling is associated with fibrosis and oxidative stress,^81^ and negatively correlated with cell proliferation via regulation of Wnt3a.^82^ Dopaminergic signaling is disrupted in inflammatory bowel disease (IBD), but D2R agonists are protective against the disease through reductions in VEGF-related vascular permeability.^83^ Finally, the hub gene, *GPR119*, a GPCR binding long-chain fatty acids and dopamine derivatives,^84^ is reduced in Crohn’s disease (CD)^85^ and correlates with tissue-specific dopamine synthesis.^86^ Altogether, functional comparisons of CD45^+^ cardiac and classical NEC ROIs indicate a relative dearth of VEGF and D2R signaling in cardiac NEC, while classical NEC is associated with increased *HOXB7* expression as a likely function of EMT-associated attempts at epithelial restitution.^87^

### Classical NEC PanCK^+^ ROIs are defined by pathogen-induced cytokine storm signaling

When comparing gene expression within PanCK^+^ ROIs of cardiac and classical NEC ileum, DEGs (Figures 4A and S11B) used to construct the largest PPI subnetwork (Figure 4B) mapped functionally to immune chemotaxis, immunoglobulin A (IgA) production, interleukin 10 (IL-10) signaling, erythrocyte metabolism, and carbonate dehydratase activity (Figure 4C), the latter involved in regulation of cellular pH. Hub genes with the strongest influence on pathway enrichments included *CCR1* (CC motif chemokine receptor 1), *MRC1* (mannose receptor C-type 1), *CD80*, *TLR8* (toll-like receptor 8), and *CLEC10A* (C-type lectin domain containing 10A; Figure 4D). IPA functional analysis (Figures 4E and 4F) indicates pathogen-induced cytokine storm signaling likely mediates immune chemotaxis and antioxidant/pH responses in the classical NEC epithelium. Integrated proteome and transcriptional network analysis revealed differential regulation of the KEGG pathway, small cell lung cancer (combined *p* value, 0.02769; Table S4), associated with high levels of apoptosis.

The cytokine storm signaling pathway, involving monocyte, macrophage, and neutrophil recruitment, capillary permeability and disruption of the endothelial barrier, and an insufficient anti-inflammatory response, can result in severe tissue damage.^88^ Classical NEC intestinal epithelial cell (IEC) upregulation of the anti-viral hub gene *TLR8*, potentially induced via dysbiosis,^89^ directs enrichment of CD14^+^CD16^+^ monocytes, driving mucosal inflammation.^90^ Interestingly, TLR8 binding of the CD1c^+^ dendritic cell receptor, CLEC10A, increases tumor necrosis factor alpha (TNFα), IL-8, and IL-10 secretion from these antigen-presenting cells.^91^ CCR1, a chemokine receptor expressed constitutively on the apical surface of IECs, binds proinflammatory ligands, aiding in the recruitment of inflammatory monocytes to the intestine.^92^ The transmembrane C-type lectin receptor (CLR), MRC1 (Figure S20D), functions in M2 macrophage phagocytosis of immune cells,^93^ while CD80 plays a role in T cell activation, regulation of B cells, and stimulation of EMT.^94^ While IL-10 signaling is protective against experimental NEC through modulation of intestinal inflammation,^95^ infants with classical NEC are known to suffer from a lack of IgA binding of pathogenic intestinal bacteria,^96^ one reason maternal IgA supplementation through breastfeeding is thought to be protective against the disease.^97^ Taken together, these analyses point to insufficient defenses against microbial-driven epithelial cytokine storm signaling within the classical NEC epithelium.

### Directionally concordant epithelial and immune transcriptional differences are defined by differential response to endoplasmic reticulum stress and mucosal acidosis

We next evaluated transcriptional differences in cardiac and classical NEC restricted to genes with cell-type segment concordance, or those genes differing between NEC subtypes but differing similarly in both epithelial and immune cells (Figure S15C). While classical NEC appeared more heterogeneous in this analysis, DEGs (Figures 5A and S11C) used to construct the largest PPI subnetwork (Figure 5B) largely functionally mapped to dopamine signaling (Figure 5C), similar to those comprising differentially enriched pathways in the CD45^+^ analysis (Figures 3E and 3F). However, hub genes (Figure 5D) in this cell-type concordant analysis included components of broader dopamine signaling, as well as more specific genes associated with chemosensory functions (e.g., *RAX2* [retina and anterior neural fold homeobox 2], *NRL* [neural retina leucine zipper], *CNGB1* [cyclic nucleotide gated channel subunit beta 1], and *CRX* [cone-rod homeobox]). Interestingly, several of these chemosensory genes are known to function in fetal retinal development and, to our knowledge, their expression in the human intestine has not previously been reported. Additionally, IPA functional analysis (Figures 5E and 5F) indicated neuronal cAMP response element-binding protein (CREB) signaling and phagosome formation, both involved in the mucosal response to reduced pH, were significantly enriched in classical NEC. Integrated proteome and transcriptional network analysis also indicated differential regulation of the reactome pathway, Fc gamma receptor (FCGR)-dependent phagocytosis (combined *p* value, 0.01904; Table S4).

Acidosis associated with acute intestinal inflammation, such as that of NEC, is thought to result, in part, through neutrophil-epithelial crosstalk. Neutrophil recruitment to sites of tissue infection or damage is required for pathogen elimination, but collateral tissue damage occurs via neutrophil release of proinflammatory reactive oxygen species (ROS) and proteases. The accumulation of these noxious compounds, as well as the depletion of microenvironmental oxygen associated with neutrophil migration, stabilizes epithelial hypoxia-inducible factor and reduces extracellular pH through metabolic shifts in IECs favoring glycolytic production of lactate.^98^ IECs convert adenosine triphosphate (ATP) generated by migrating neutrophils to adenosine, triggering apical expression of the IEC Cl^−^/HCO_3_^−^ transporter, SLC26A3 (Figure S21A), via CREB/cAMP signaling in an effort to buffer intra- and extracellular drops in pH.^98^^,^^99^ Simultaneously, CREB/cAMP signaling, the signaling pathway most enriched in classical NEC in this cell-type segment concordant analysis (e.g., *CREB5*, Figure S21B), stimulates intracellular calcium flux in neutrophils to promote degranulation and increased bactericidal activity in the intestinal lumen. Reduced extracellular pH also triggers macrophage phagocytosis to clean damaged tissue of apoptotic cells.^100^ Genes associated with macrophage activation and phagocytosis (e.g., *MRC1* (Figure S20D), *CSF2RB* [colony stimulating factor 2 receptor subunit beta]) are upregulated in classical NEC. Conversely, IPA predicted reduced cardiac NEC expression of RNF186 (RING finger protein 186), a protein responsible for polyubiquitination of substrates and regulation of ER stress within the intestinal epithelium.^101^ Reduced RNF186 expression is associated with defective unfolded protein response (UPR) during endoplasmic reticulum (ER) stress, increased intestinal permeability, dysfunctional autophagy, reduced bacterial clearance, increased susceptibility to intestinal inflammation, and IEC apoptosis.^101^^,^^102^ In addition, the cannabidiol receptor, GPR55, is protective against ER stress,^103^ but its expression is significantly reduced in cardiac NEC. Finally, reduction in *ERAS* (ER-associated RNA silencing) expression in cardiac NEC indicates dysfunctional capacity to maintain ER homeostasis through degradation of misfolded proteins.^104^^,^^105^ Altogether, mucosal acidosis prompted through neutrophil migration and activation characterizes transcriptional signatures within the classical NEC intestinal epithelium and immune cells, while a dysfunctional response to ER stress paints a transcriptional signature of apoptosis, reduced barrier function, and reduced pathogen clearance in cardiac NEC.

### Pathogen-induced cytokine storm signaling defines gene interaction differences in classical and cardiac NEC

Studies investigating transcriptional regulation of NEC pathogenesis generally focus on intestinal epithelial or immune effects independently, largely ignoring potential integrations of these tissue responses in driving disease pathogenesis. To explore the degree to which cell-type segment (immune or epithelial) modulates transcriptional differences in classical and cardiac NEC, we explored the interaction of cell-type segment with NEC subtype. These genes changing expression between NEC subtypes discordant in the two cell-type segments are also referred to as interaction genes (Figure S16D) and indicate additive or subtractive effects beyond those at the regulatory level. Interaction DEGs (Figures 6A and S11D) used to construct the largest PPI subnetwork in this analysis (Figure 6B) mapped largely to chemokine receptor-binding and erythrocyte metabolism (Figure 6C), components of the innate immune response. Hub genes (Figure 6D) in this interaction gene analysis largely overlapped with pathogen-induced cytokine storm signaling. In particular, genes associated with antibacterial processes (*LCN2*, lipocalin 2), leukocyte activation and recruitment (e.g., *CX3CR1* [C-X3-C motif chemokine receptor 1], *CCR1*, *CXCL6*, *CCL20* [CC motif chemokine ligand 20]), and cytokine production and associated consequences (e.g., *CD80*, *SLC26A3*) are well-represented among these DEGs. Additionally, similar to analysis of PanCK^+^ differentially expressed pathways (Figures 4E and 4F), IPA functional analysis (Figures 6E and 6F) indicates pathogen-induced cytokine storm signaling and S100 family signaling pathways are enriched to the greatest extent in the classical NEC epithelium and comparatively minimally enriched within the cardiac NEC epithelium. Because this analysis, by definition, discriminates among all 4 groups (cell-type and NEC subtype), pathway analysis can rank enrichment by cell-type segment and NEC subtype. Thus, expression differences in cell-segment and NEC subtype are perhaps easiest to visualize in Figure 6G, where gene expressions are averaged across samples within cell-segment and NEC subtype. Integrated proteome and transcriptional network analysis also indicates differential regulation of the KEGG proteasome and focal adhesion pathways (combined *p* values, 0.02553 and 0.02887, respectively) and reactome pathways, TNFR2 (TNF receptor 2) non-canonical nuclear factor kappa B (NF-kB) pathway, host interaction of HIV factors, and interleukin-10 signaling (combined *p* values 0.02366, 0.02762, and 0.04769, respectively; Table S4).

As components of the pathogen-induced cytokine storm signaling pathway, genes associated with redox-mediated regulation of microbial growth and host defense and innate immune activation and trafficking were upregulated substantially in the classical NEC epithelium (*CCL20*, *CCL28*, *LCN2*, and *NOX1* [NADPH oxidase 1]; Figure S21C) and immune (*CCR1*, *CX3CR1*, and *CXCL6*) compartments, respectively. Genes overlapping with the S100 family signaling pathway were also differentially expressed in NEC subtype and cell-type segment gene interactions. *FFAR2* (free fatty acid receptor 2) is maximally expressed in classical NEC immune and epithelial compartments and least expressed in cardiac NEC epithelium. An upstream enhancer of CREB-signaling and receptor for short-chain fatty acids (SCFAs), reduced FFAR2 spurs epithelial barrier defects and epigenetic dysregulation of suppressors of inflammation.^106^^,^^107^ *POSTN* (periostin), maximally expressed in classical NEC epithelium and least expressed in cardiac NEC epithelium, is upregulated in IBD and involved in macrophage migration and NF-kB-mediated intestinal inflammation.^108^ *C10ORF99* (chromosome 10 open reading frame 99), maximally expressed in classical NEC epithelium and least expressed in cardiac NEC epithelium, regulates proinflammatory signaling and reduction of epithelial barrier function.^109^ Importantly, recent work has suggested the antimicrobial peptide encoded by this gene, AP-57, may exhibit broad antimicrobial, antitumor, and DNA binding effects, and participates in T cell trafficking within the intestine.^110^ *CEACAM7* (carcinoembryonic antigen-related cell adhesion molecule 7), maximally expressed in classical NEC epithelium and least expressed in cardiac NEC epithelium, is an intercellular adhesion molecule highly expressed in IECs. Dysbiosis, particularly of adherent-invasive and enterotoxigenic *Escherichia coli* (AIEC), has been shown to induce acute expression of this gene in IBD, while SCFA treatment reduces CEACAM7 expression.^111^^,^^112^ Finally, *SLC26A3*, also differentially expressed in directionally concordant analyses (Figure S21A), is expressed at high levels in the classical NEC epithelium, as it is in human CD and animal models of colitis,^113^ and low levels in the cardiac NEC epithelium. High expression of SLC26A3 represents an adaptive and protective phenotype to mucosal acidification, while misexpression or reduced expression of SLC26A3 results in congenital chloride diarrhea.^114^ While SLC26A3 is a marker for mature enterocytes,^115^ the stark difference in *SLC26A3* expression is unlikely to represent developmental differences in our study, as the relatively older cardiac NEC infants exhibit lower expression of this transporter gene.

Interestingly, multiple genes associated with enteric nerve function and homeostatic body weight regulation are upregulated in classical NEC. Expression of *GPR119*, a hub gene upregulated in classical NEC in the CD45^+^ analysis (Figure 3D), is maximal in classical NEC immune cells and least expressed in the cardiac NEC epithelium. A fat sensor regulated by bile acid composition and microbial metabolites,^116^^,^^117^ GPR119 induces GLP-1 (glucagon-like peptide 1) and PYY (peptide YY) release,^118^ hormonally regulating gastric emptying and food intake during energy deficits through activation of cAMP signaling.^119^ *NMUR1* (neuromedin U receptor 1), maximally expressed in classical NEC immune cells and least expressed in cardiac NEC epithelium, is involved in body weight regulation in adults and promotes hypophagia in animal studies.^120^ Interestingly, NMUR1 also promotes immune cell activation and cytokine production in neurogenic inflammation, potentially also through cAMP-mediated processes.^121^

In cardiac NEC, genes associated with endothelial migration and angiogenesis were downregulated. *COL25A1* (collagen type XXV alpha 1 chain) was maximally expressed in classical NEC epithelium and least expressed in cardiac NEC epithelium. As a marker for angiogenic pericytes^35^ and enteric neurons,^122^ reduced levels of this neuronal collagen regulated by IL-22^123^ may result in dysmotility. *PlGF (placental growth factor)*, maximally expressed in classical NEC immune cells and least expressed in cardiac NEC epithelium, is a marker of IBD-associated pathological angiogenesis with the capacity to alter growth, migration, and survival of endothelial cells.^124^ Reduced expression of this VEGF subfamily gene may represent a deficient response to localized hypoxia within the cardiac NEC epithelium.

Finally, expression of genes associated with ER stress is evident in cardiac NEC. *HMGCS2* (3-methoxy-3-methylglutaryl-CoA synthase 2) is maximally expressed in classical NEC epithelium and least expressed in cardiac NEC epithelium. Acutely involved with intestinal stem cell renewal, HMGCS2 is lost during active IBD and strongly associated with ER stress.^125^ *CA4*, maximally expressed in classical NEC epithelium and least expressed in cardiac NEC epithelium, is central to tissue metabolism. Inhibition of this enzyme in other tissues results in significant ER stress.^126^ *CACNB3* (calcium voltage-gated channel auxiliary subunit beta 3, Figure S21D) and *CACNA2D3* (calcium voltage-gated channel auxiliary subunit alpha 2/delta 3, Figure S22) are expressed maximally in the classical NEC epithelium and immune cells, respectively, and least expressed in cardiac NEC epithelium. Intimately associated with contractility and intestinal motility,^127^ ileal expression of these ion channel genes regulates calcium overload during ischemia-reperfusion injury^128^ and may contribute to malabsorption in the context of short bowel syndrome,^129^ a postoperative condition commonly affecting infants following resection of significant bowel length. Further, CACN family proteins are often downstream of activated CREB signaling,^130^ a pathway indicated to be enriched in classical NEC in the concordant gene expression analysis (Figures 5E and 5F).

## Discussion

Recent studies have indicated that cardiac NEC may be a distinct NEC subtype with an etiology different than that of classical NEC.^17^^,^^18^^,^^19^^,^^20^ However, the relative rarity of cardiac NEC cases has deemed these discussions primarily speculative. Here, we sought to establish differences in cardiac and classical NEC epithelial and immune compartments, and their respective interplays. To meet this objective, we utilized distal ileal tissues from a historical repository from a level IV NICU in an attempt to identify etiological differences between cardiac and classical NEC.

A key advantage of this study is the use of novel bioinformatics and pathway analysis programs in evaluating relationships among key genes expressed. For example, our use of SpatialOmicsOverlay in portraying averaged gene expression on original histological samples is, to our knowledge, the first manuscript to do so outside of protocol-centered publications. In addition to substantiating involvement of pathways reported in NEC pathophysiology, our approach highlights processes not previously described in cardiac (e.g., dopaminergic signaling) and classical (e.g., genes associated with enteric nerve function and systemic metabolism [*NMUR1* and *GPR119*]) NEC.

Our data suggest a classical NEC phenotype, in direct comparison to that of cardiac NEC, initiated through dysbiosis and involving induction of elevated CREB signaling, extracellular acidosis, and progression of cytokine storm signaling. Though toll-like receptor 4 (TLR4) signaling is commonly referenced,^131^^,^^132^^,^^133^ CREB is a downstream mediator of TLR4 signaling,^134^ resulting in TNFα, IL-6, and COX-2 (cyclooxygenase-2) expression, and has been proposed and investigated in classical NEC pathogenesis. In both human and experimental murine NEC tissues, cAMP production and CREB activation are increased before IEC death ensues.^135^ Induced by a wide variety of inflammatory and proliferative signals, CREB is unique in its potential to both engage and inhibit NF-kB signaling,^136^ a pathway generally believed central to classical NEC pathogenesis.^137^^,^^138^ Interestingly, recent studies have indicated bacteria upregulate CREB signaling in macrophages, promoting both macrophage and bacterial survival through reduced host necroptosis.^139^ However, given the purported pathological role of macrophages in classical NEC,^140^^,^^141^^,^^142^ enhanced monocyte and macrophage survival may not prove beneficial to the host.

Conversely, in cardiac NEC, a phenotypic signature arises for reduced VEGF-induced angiogenesis and enhanced ER stress, phenomena closely related to tissue hypoxia and reduced perfusion.^21^ Insufficient VEGF production has been observed in NEC,^68^^,^^69^ but its relative reduction in cardiac NEC is somewhat surprising and may be representative of an underlying inability to compensate for reduced intestinal perfusion. The resulting hypoxia from ischemia/reperfusion events, as well as increased energetic demands and oxidative stress, are inducers of ER stress in the small intestine.^143^ As the site of protein production in the cell, the ER enacts UPR in an attempt to ameliorate negative effects, such as calcium overload, on cell function.^144^ However, UPR signaling is unable to alleviate prolonged ER stress, resulting in apoptosis.^143^ Interestingly, ER stress-induced apoptosis appears to disproportionately impact Paneth cells,^145^ epithelial cells critical to stem cell support and host defense, with a likely role in NEC pathogenesis.^146^^,^^147^^,^^148^^,^^149^

While infant samples were matched to the extent possible, variables associated with gestational age and respective NEC pathologies, such as mode of delivery and some clinical features (e.g., thrombocytopenia), differed significantly among infants in this study. Cardiac NEC is more common in term or near-term infants, but we included tissues from a preterm infant with cardiac NEC in an attempt to limit transcriptional differences that might be attributed to differing developmental stages. C-section deliveries are far more common among preterm infants compared with term.^150^ Though studies are mixed in implicating C-section delivery as a risk factor for NEC,^151^^,^^152^ the altered seeding of the neonatal microbiome induced by C-section delivery likely increases neonatal NEC risk indirectly. The microbiome of infants born via C-section reflects bacteria found on the maternal skin surface rather than the vaginal canal, and compared to that of infants born vaginally, includes high levels of opportunistic pathogens (e.g., *Staphyloccus*, *Enterobacter*, and *Klebsiella*) and low levels of protective Bifidobacteria.^153^ While both infants with cardiac NEC were born vaginally, 80% of classical NEC cases in this study were born via C-section. Though we are unable to confirm microbiome alterations via sequencing due to the archival nature of these tissue samples, we can reasonably hypothesize blooms of potential pathogens and increased direct microbial contact with the intestinal epithelium is responsible for much of the pathogen-induced cytokine storm signaling phenotype that emerges as a signature of classical NEC. Importantly, the signal of pathogen-induced cytokine storm signaling is strongly represented in classical NEC despite the direct comparison to cardiac NEC, a clinically similar disease. Additionally, infants with classical NEC were moderately (*n* = 2) or severely (*n* = 3) affected by thrombocytopenia, while both infants with cardiac NEC had normal platelet counts. As in neonatal sepsis,^154^ thrombocytopenia associated with classical NEC is thought to result from excessive production of thrombopoietin, driving functional “exhaustion” of the bone marrow. In the case of classical NEC, excessive lipopolysaccharide-induced TLR4 signaling likely contributes to thrombocytopenia and bone marrow suppression.^155^ While platelet depletion may influence intestinal pathways and network analyses both directly and indirectly, the utilization of comparisons centered exclusively on cell-type abundance-independent DEGs (Figures 3, 4, 5, and 6) excludes many of the pathways and networks uniquely associated with platelet abundance/activation. While variables such as gestational age, mode of delivery, and clinical/demographic differences can contribute to transcript differences, we attempted to compensate for our relatively small sample size through enhanced inclusion of ROIs (*n* = 42 classical NEC ROIs, *n* = 14 cardiac NEC ROIs).

These first etiological findings on cardiac NEC are important, as the current standard-of-care for these NEC subtypes is identical despite the growing belief that NEC subtype etiologies might differ. For example, suggestions for pre- and/or probiotic therapeutics for infants at-risk of classical NEC,^156^ the pathogenesis of which is believed to require dysbiosis,^157^ may not extrapolate well to infants suffering from cardiac NEC, a NEC subtype potentially, according to our data, less dependent upon shifts in microbial composition.

### Limitations of study

A common limitation of transcript-based studies is the potential lack of correlation between transcriptional and proteomic changes in the tissues of interest, resulting in discovered relationships that likely cannot move beyond associative in nature. Though inexact as only a subset (*n* = 2 cardiac NEC and *n* = 2 classical NEC) of our patient cohort samples were evaluated via targeted proteomic panels, we compared raw counts for those genes with corresponding protein measurements (Figures S23–S34). Many, but not all, gene counts directionally corresponded with protein counts. While homeostatic protein levels are primarily determined by mRNA levels, discrepancies in our gene-protein correlations are not unexpected, as synthesis of proteins from respective mRNAs is generally delayed during state transitions, including periods of rampant inflammation.^158^ However, the ability to run full proteomics on the same tissues and on a similar platform at the initiation of this study was not yet available, and gene-specific RNA-to-protein conversion factors, independent of tissue type,^159^ are generally not available for genes/proteins of interest here.

Further, as a transcript-based study incorporating rare patient tissues from diseases considered to be heterogeneous, our study is subject to several additional limitations. Because patient tissues, especially those of the relatively rare cardiac NEC, are difficult to acquire, we included only a small number of samples (*n* = 5 classical NEC and *n* = 2 cardiac NEC) that were well-matched for disease severity. Additionally, our patient cohort was predominantly male (*n* = 6). This demographic difference likely reflects the increased risk of preterm birth of fetal males,^160^ as sex is not considered to significantly influence NEC incidence.^161^ Cardiac NEC often occurs in the colon, in lieu of or in addition to the ileum, but in order to maintain physiological consistency among samples, we included only infants with ileal involvement. In addition, in order to delineate cardiac and classical NEC-specific signatures, independent of confounding overt inflammatory signatures common to both NEC subtypes, we directly compared transcriptomes of cardiac NEC ROIs to those of classical NEC rather than comparing each NEC subtype to a “healthy” control. While this analytical technique does not allow for a true, traditional “control”, truly healthy tissue is not obtainable from preterm or term infants with no bowel indications, resulting in traditional controls representing “healthy” tissues resected for conditions such as intestinal atresia. Further, by directly comparing NEC subtype signatures, we could eliminate generic mucosal inflammatory signals, thereby highlighting only relevant differences specific to the NEC subtypes, as has similarly been done previously.^162^

## Resource availability

### Lead contact

Further information and requests for resources and reagents should be directed to, and will be fulfilled by, the lead contact, Dr. Kathryn Burge (kathryn-burge@ouhsc.edu).

### Materials availability

This study did not generate new unique reagents.

### Data and code availability

Spatial transcriptomics, targeted proteomics data, and full-size, high-resolution images have been deposited to the Gene Expression Omnibus (GEO) repository (GEO: GSE268423) for public access. This accession number is also listed in the key resources table. All original code has been deposited on Github and is publicly available (URL listed in the key resources table). Any additional information required to reanalyze the data reported in this paper is available from the lead contact upon request.

## Acknowledgments

The authors thank the OUHSC Department of Pathology for providing samples of human intestinal resection, and the Histology and Immunochemistry Core at the University of Oklahoma Health Sciences Center, the Tissue Management Shared Resource at the University of Texas Southwestern Medical Center, and the Microarray and Immune Phenotyping Core at the University of Texas Southwestern Medical Center for technical assistance with this project. We thank Dr. David Dyer, Dr. Bishwa Sapkota, and Edgar Scott for consultation during data analysis for this manuscript. K.Y.B. was supported by the 10.13039/100009633NIH/NICHD (R21HD112659), NIH/10.13039/100000057NIGMS (P20GM134973) and the 10.13039/501100024932Children’s Health Foundation (CHF). C.G. was supported by the NIH/NIGMS (P30GM149376 and U54GM104938). J.D.W. was supported by the NIH/NIGMS (P30GM149376 and U54GM104938). H.C. was supported by the 10.13039/100009633NIH/NICHD (R01HD109784), NIH/10.13039/100000057NIGMS (P20GM134973), and CHF.

## Author contributions

H.C. and A.G. conceived of the study design and assisted with patient sample selection; Z.Y. assisted with patient sample selection and performed histopathological analysis; K.Y.B., C.G., and H.Z. performed bioinformatic and statistical analyses; K.Y.B. analyzed data, prepared figures, drafted the original manuscript, and contributed to its review and editing; J.V.E. visualized samples with a slide scanner; A.P.W. analyzed data and prepared figures; H.C., J.D.W., and K.Y.B. administered the project and supervised the work; C.G., H.Z., A.P.W., A.G., Z.Y., J.V.E., J.D.W., and H.C. reviewed and edited the manuscript; K.Y.B. and H.Z. had unrestricted access to all data; All authors agreed to manuscript submission, read and approved the final draft, and take full responsibility for its content, including the accuracy and fidelity of all data and statistical analyses.

## Declaration of interests

H.C. is a scientific advisory board member for the NEC Society (501c-3).

## STAR★Methods

### Key resources table

**Table:** 

REAGENT or RESOURCE	SOURCE	IDENTIFIER
**Antibodies**		

Alexa Fluor® 594 monoclonal mouse anti-panCK (AE-1/AE-3) diluted to 2 μg/mL	Novus	Cat#NBP2-33200DL594; RRID: AB_289577
Alexa Fluor® 647 monoclonal mouse-*anti*-CD45 [2B11 + PD7/26] diluted to 5 μg/mL	Novus	Cat#NBP2-34528AF647; RRID: AB_2864384
Alexa Fluor 488 monoclonal rabbit-*anti*-carbonic anhydrase 9 [EPR4151(2)] diluted 1:100	Abcam	Cat#ab225075; RRID: AB_2943135

**Biological samples**		

Human Ileal tissue	University of Oklahoma Health Sciences Center Department of Pathology	Table S1

**Critical commercial assays**		

GeoMx® Whole Transcriptome Atlas Human RNA Probes for NGS	NanoString	Cat#121401102; Lot#HWTA12002 (TAP); Lot#HWTA21003 (UTSW)
GeoMx® Solid Tumor TME Morphology Kit diluted 1:20 (PanCK) and 1:10 (CD45)	NanoString	Cat#121300310; Lot#210910-1 (PanCK), Lot#211109-1 (CD45); RRID: AB_2935724
GeoMx® Nuclear Stain Morphology Kit diluted 1:10	NanoString	Cat#121300303; Lot#20220129
Immune Cell Profiling Panel Human Protein Core	NanoString	Cat#GMX-PROCO-NCT-HICP-12; Lot#0474026; RRID: AB_2935726
Immune Activation Status Panel Human Protein Module	NanoString	Cat#GMX-PROMOD-NCT-HIAS-12; Lot#0474032; RRID: AB_2935733
Immune Cell Typing Panel Human Protein Module	NanoString	Cat#GMX-PROMOD-NCT-HICT-12; Lot#0474035; RRID: AB_2935731
PI3K-Akt Signaling Panel Human Protein Module	NanoString	Cat#GMX-PROMOD-NCT-HPI3K-12; Lot#474128; RRID: AB_2935737
Pan-Tumor Panel Human Protein Module	NanoString	Cat#GMX-PROMOD-NCT-HPT-12; Lot#0474038; RRID: AB_2935734
nC Cell Death Panel Human Protein Module	NanoString	Cat#GMX-PROMOD-NCTHCD-12; Lot#0474050
MAPK Signaling Panel Human Protein Module	NanoString	Cat#GMX-PROMOD-NCT-HMAPK-12; Lot#0474047; RRID: AB_2935736

**Deposited data**		

NanoString GeoMx® RNA (FASTQ) data	This manuscript	GEO: GSE268423
Raw and analyzed NanoString GeoMx® targeted protein data	This manuscript	GEO: GSE268423
Single-cell RNA-sequencing data	Mabbott et al.^36^	http://biogps.org/dataset/BDS_00013/primary-cell-atlas/
Single-cell RNA-sequencing data	Elmentaite et al.^35^	https://www.humancellatlas.org/

**Software and algorithms**		

BioRender		https://www.biorender.com; RRID: SCR_018361
Prism v10.2.3	GraphPad	www.graphpad.com
Aperio ImageScope v12.4.3	Leica Biosystems	https://www.leicabiosystems.com/us/digital-pathology/manage/aperio-imagescope/; RRID: SCR_018361
OlyVIA v2.9.1	Olympus Life Science	https://www.olympus-lifescience.com/en/downloads/detail-iframe/?0[downloads][id] = 847252030; RRID: SCR_0161667
GeoMx DSP Data Center v3.0.0.111	NanoString	https://www.nanostring.com/products/geomx-digital-spatial-profiler/geomx-data-center
R v4.3	CRAN	https://www.r-project.org; RRID: SCR_001905
gplots R package v3.1.3	CRAN	https://cran.r-project.org/web/packages/gplots/index.html
ggplot2 R package v3.4.2	CRAN	https://cran.r-project.org/web/packages/ggplot2/index.html; RRID: SCR_014601
GeoMxTools R package v3.4.0	Bioconductor	https://www.bioconductor.org/packages/release/bioc/html/GeomxTools.html; RRID: SCR_023424
Seurat v4.0.3	Stuart et al.^163^	https://cran-r-project.org/web/packages/Seurat/index.html RRID: SCR_007322
SpatialDecon R package v1.10.0	Bioconductor	https://www.bioconductor.org/packages/release/bioc/html/SpatialDecon.html
edgeR v3.42.4	Bioconductor	https://bioconductor.org/packages/release/bioc/html/edgeR.html; RRID: SCR_012802
Celldex R package v3.17	Bioconductor	http://bioconductor.org/packages/release/data/experiment/html/celldex.html
GraphBio	Zhao and Wang^164^	http://www.graphbio1.com/en/
SRplot		https://www.bioinformatics.com/cn/srplot
CytoScape v3.10.0	Shannon et al.^165^	http://cytoscape.org; RRID: SCR_003032
stringAPP v2.0.1	Doncheva et al.^166^	https://apps.cytoscape.org/apps/stringapp
CytoHubba plug-in v0.1	Chin et al.^167^	https://apps.cytoscape.org/apps/cytohubba; RRID: SCR_017677
ClueGo v2.5.10	Bindea et al.^168^	https://apps.cytoscape.org/apps/cluego; RRID: SCR_005748
Ingenuity Pathway Analysis v94302991	Qiagen	https://digitalinsights.qiagen.com/products-overview/discovery-insights-portfolio/analysis-and-visualization/qiagen-ipa/; RRID: SCR_008653
PaintOmics 4 v1.00	Liu et al.^169^	https://paintomics.org/; RRID: SCR_021859
SpatialOmicsOverlay v1.2.1	Bioconductor	https://www.bioconductor.org/packages/release/bioc/html/SpatialOmicsOverlay.html
objSeurat_UMAPs.rda	This paper	https://github.com/ttgeo/codeShaabanPaper/blob/master/objSeurat_UMAPs.rda
Convolution.rda	This paper	https://github.com/ttgeo/codeShaabanPaper/blob/master/convolution.rda
chaabanProcessing_toSend.rmd	This paper	https://github.com/ttgeo/codeShaabanPaper/blob/master/chaabanProcessing_toSend.rmd
chaabanProcessing_toSend.md	This paper	https://github.com/ttgeo/codeShaabanPaper/blob/master/chaabanProcessing_toSend.md

### Experimental model and subject details

#### Human subjects

Patient samples were collected during bowel resection for cardiac (*n* = 2 patients) or classical (*n* = 5 patients) NEC. The acquisition, analysis, and storage of deidentified human samples, including a waiver of informed consent and Health Insurance Portability and Accountability Act waiver of authorization, was approved by the University of Oklahoma Health Sciences Center Internal Review Board (OUHSC-IRB-11154). The Institutional Review Board granted a waiver of informed consent under 45 CFR § 46.116 based on review and determination that this research meets the following requirements: (1) the research involves no more than minimal risk to subjects; (2) the research could not practicably be carried out without the requested waiver; (3) the waiver will not adversely affect the rights and welfare of the subjects. Table S1 contains demographic and clinical data for all patients. Participant information on race was self-reported, while participant information on sex and gestational age was physician-reported. Information on gender, ethnicity, and socioeconomic status was not collected. Investigators were not blinded as to NEC subtype. Statistical analysis was performed in GraphPad Prism using Student’s T-test or Fisher’s exact tests, as appropriate.

### Method details

#### Histology

Ileal tissues were fixed with 10% neutral buffered formalin and embedded in paraffin blocks. Freshly cut and sequential 5 μm tissue sections were mounted onto charged slides for histopathological analysis by H&E staining. Slides were photographed using either a high-resolution Aperio CS bright-field scanner (Leica Biosystems) at 20× magnification and analyzed via Aperio ImageScope v12.4.3 software (Leica Biosystems) or an Olympus VS120 Virtual Slide System (Olympus Life Science) at 20× magnification and analyzed with OlyVIA v2.9.1 (Build 13771, Olympus Life Science).

#### GeoMx DSP of transcriptome

Tissue sections were processed using either the Technology Access Program (TAP) at NanoString Technology Laboratories (*n* = 2 cardiac and *n* = 2 classical NEC; Seattle, WA, USA) or the Tissue Management Shared Resource and Histology and Immunochemistry Core at the University of Texas Southwestern Medical Center (UTSW; *n* = 3 classical NEC; Dallas, TX, USA). Figure S1 illustrates the GeoMx DSP workflow. Slides were prepared according to the GeoMx DSP slide preparation user manual (MAN-10087; NanoString). Tissue sections with the NanoString TAP were stained with visualization markers for panCK (2 μg/mL; NBP2-33200AF594, clone: AE-1/AE-3, Novus), CD45 (5 μg/mL; NBP2-34528AF647, clone: 2B11 + PD7/26, Novus), and CA9 (1:100; ab225075, Abcam). Tissue sections at UTSW were stained with visualization markers for panCK and CD45 (1:20 and 1:10, respectively; GeoMx Solid Tumor TME Morphology Kit). In all samples, nuclei were counterstained with SYTO13 (1:10; GeoMx Nuclear Stain Morphology Kit). All slides were incubated with custom probes included in the human GeoMx Whole Transcriptome Atlas (WTA) gene set, encompassing 18,677 gene targets. Selection of ROIs (∼6/patient) was performed based upon histopathological NEC features gleaned from H&E staining (e.g., sloughed villi and lymphocyte infiltration, but excluding clearly necrotic tissue) and immunofluorescent segmentation by anti-CD45 (immune-rich) and anti-panCK (epithelial-rich) staining. To the extent possible, ROIs containing intestinal epithelium, vasculature, and submucosal compartments representative of the entire range of ileal NEC pathology were selected within each tissue section. Exposure to ultraviolet light released DNA oligo tags from RNA probes within ROIs into individual wells of a microtiter plate. Oligo tags were sequenced using Illumina NGS, and the GeoMx NGS Pipeline (DND) was used to filter, demultiplex, and convert FASTQ to DCC files. High-resolution, full-size immunofluorescent images and FASTQ files were deposited to GEO GSE268423.

#### GeoMx DSP of targeted proteome

Tissue sections sequential to those evaluated for the transcriptome were processed using the NanoString TAP (*n* = 2 cardiac and *n* = 2 classical NEC) and stained as indicated above and according to the GeoMx DSP slide preparation user manual (MAN-10087). Slides were incubated with a custom antibody cocktail composed of 77 target proteins from the Human Immune Cell Profiling, Human Immune Activation Status, Human Immune Cell Typing, Human PI3K-Akt-Signaling, Human Pan-Tumor, Human nC Cell Death, and Human MAPK Signaling panels (Table S3). Selection of ROIs (∼6/patient) was performed as indicated above. Targeted proteomic data were deposited to GEO GSE268423.

### Quantification and statistical analysis

#### GeoMx NGS data normalization and analysis

NanoString GeoMx sequencing accuracy is enhanced through indirect quantification of probe tags rather than transcript numbers.^170^ Raw DCC files from GeoMx DSP runs generated by both NanoString TAP and UTSW were imported into R using the GeoMxTools R Package. All ROIs met a sequencing threshold >10,000 reads and a sequencing saturation (1-% unique sequencing reads) above 50%, and all genes were expressed above the limit of quantitation (LOQ, the geometric mean of the negative probes + [the geometric standard deviations of the negative probes∗2]) in at least 5% of ROIs. RNA probe counts were normalized using the geometric mean of the negative control probes (Q3 normalization).^171^ Probes not detected in at least 5% of all ROIs were excluded. Normalized data were coerced into a Seurat object for subsequent analysis^163^ using the SCTransform Integrate Data workflow of the Seurat R Package. Data analysis was performed in R, and graphics were produced using ggplot2 unless otherwise noted.

#### Dimensionality reduction and clustering

After selection of highly variable genes through non-parametric Wilcoxon rank-sum test and Benjamini-Hochberg adjustments, the integrated data from all 7 patient samples and associated ROIs were subjected to principal component analysis (PCA) and nonlinear dimensionality reduction with uniform manifold approximation and projection/stochastic neighbor embedding (UMAP/sSNE) via the ‘RunUMAP’ function, using the top 3000 most variable genes. ROI clustering was accomplished by the ‘FindClusters’ function (resolution = 0.015), using the K-nearest neighbor (KNN) approach with smart local moving (SLM) modularity optimization. 2D UMAP was used to visualize clustering results. The Seurat function, ‘FindMarkers’, was used to identify differential expression among cell-type segments and NEC subtypes and conserved cell-type markers within each cluster. Expression profiles were visualized using the ‘DoHeatmap’ function. Adjusted *p* < 0.05 and |log_2_FoldChange(FC)|>0.25 was used to define DEGs within ROI clusters. Mixed cell deconvolution in each ROI was performed using the Celldex R package and the single cell sequencing survey, Human Primary Cell Atlas (HPCA).^36^ Using gene expression data, abundance of each cell type within each observation was estimated and the dominant category was assigned as ROI cell type. Relative distribution and enrichment of cell types across clusters, NEC subtypes, and immune/epithelial segments were studied and visualized using Correspondence Analysis tools in R.

#### Mixed cell deconvolution

Mixed cell deconvolution was also performed for each ROI using the SpatialDecon R Package and both primary and granular matrices derived from cell profiles defined in the Human Cell Atlas (HCA) intestine dataset.^35^ This secondary method of deconvolution was selected due to the option of corresponding reverse deconvolution and identification of cell abundance-independent genes. SpatialDecon utilizes constrained log-normal regression and nuclei segmentation, reducing skewness and variation within the transcriptional data and improving upon cell-type percentages with estimated cell counts, respectively.^67^ Median cell abundances were compared using Wilcoxon signed rank test and plotted using boxplots.

#### Reverse deconvolution

Expression of each gene was predicted from cell abundance estimates generated through SpatialDecon, and reverse deconvolution residuals were calculated using the edgeR R package function, ‘Voom’, as the log2 fold change (observed-expected).^172^ Genes with low correlation and high residual standard deviation (Figure S11) are likely cell-type abundance-independent, while the complete list of DEGs likely contains genes dependent upon cell-type abundance and additional, unexplained variance.^67^ *p*-values were adjusted (*q*-values) for multiple testing using Benjamini-Hochberg methods, and thresholds for DEG significance are defined in Table S2. Functional analysis of genes with low correlation and high residual standard deviation is included in the main text, while functional analysis of full DEG lists is included in Supplemental Information.

#### Individual DEG comparisons and interactions

DEGs were defined as a function of segment (Seg, either CD45^+^ or PanCK^+^) and type (Typ, cardiac NEC or classical NEC). For each gene, Seg+Typ+Seg∗Typ returns 4 coefficients: intercept, Seg, Typ, and Seg:Typ. Six comparisons were thus achieved: cardiac NEC immune vs. classical NEC immune (Seg+Seg:Typ), cardiac NEC epithelial vs. classical NEC epithelial (Seg+Seg:Typ), cardiac NEC immune vs. cardiac NEC epithelial (Typ+Typ:Seg), classical NEC immune vs. classical NEC epithelial (Typ+Typ:Seg), genes differing significantly by type but not significantly by segment (Typ), and interaction genes (genes significantly differing in both type and segment; seg:Typ). Significant genes in each comparison were visualized with heatmaps using the ‘heatmap.2’ function in the R gplots package.

#### Protein-protein interaction (PPI) networks

*Q*-values and FC values for DEGs were imported to CytoScape.^165^ Overlapping gene-protein interaction networks were constructed via query of the STRING (search tool for the retrieval of interacting genes) database (confidence cutoff, 0.4) using the Cytoscape stringApp,^166^ where nodes indicate discrete genes and edges represent connections between genes. The largest subnetwork was selected for each analysis, and annotation of functional enrichment was performed with ClueGO,^168^ using GO Biological Functions, GO Immune System, GO Molecular Function, KEGG, Reactome Pathways, and WikiPathway databases. Significantly enriched functions were defined through two-sided hypergeometric tests with Bonferroni step down correction (Bonferroni-corrected *p* < 0.05), with a minimum of 2–3 genes/pathway and a kappa score of 0.4. Hub genes were identified through degree of connectivity and shortest paths using maximal clique centrality (MCC) ranking of 15 or fewer nodes in the CytoScape plugin, CytoHubba.^167^

#### Ingenuity Pathway Analysis (IPA)

DEGs were uploaded into IPA for prediction of significantly altered or enriched pathways and regulators. Core Analysis was used to explore differential biological processes, canonical pathways, upstream transcriptional regulators, and gene networks. Significantly enriched or depleted pathways (FDR-adjusted *p* < 0.05 via Fisher’s Exact Test) were represented with bubble plots using SRPlot, and three pathways per comparison were graphed as chord plots using GraphBio,^164^ with gene overlap ≥2.

#### GeoMx protein normalization and analysis

Protein counts were normalized by negative IgG isotype control probes to account for signal-to-noise, and also to a geometric mean of housekeeping proteins (GAPDH [glyceraldehyde-3-phosphate dehydrogenase], histone H3, and ribosomal protein S6) to adjust for variable cell-type composition among ROIs. Of the 77 proteins evaluated (Table S3), 45 were expressed with a signal-to-noise ratio >3.

#### Integrated analysis of gene/protein data

Integrative analysis and visualization of transcriptomics and targeted proteomics was performed using PaintOmics 4,^169^ using KEGG and Reactome databases (Table S4). A matrix of significant features and fold-changes was uploaded, and the Fisher’s combined probability test was utilized to determine joint enrichment significance. Averaged raw gene and protein counts were plotted side-by-side to demonstrate correlations in gene/protein data, and significance was determined through Wilcoxon test (*p* < 0.05).

#### SpatialOmicsOverlay

Expression of select genes was overlayed on ROIs within original OME-TIFF images of cardiac and a subset of classical NEC samples using the R package, SpatialOmicsOverlay. To visualize gene expression on original tissue images, OME-TIFF images, segment annotations, and gene counts were used to create a SpatialOverlay object. Image and XML were extracted from OME-TIFFs, ROIs overlaid on images, and ROI gene expression colored and cropped for optimal visualization.
