# Supplementary material for: Spatial transcriptomics delineates potential differences in intestinal phenotypes of cardiac and classical necrotizing enterocolitis
Source: iScience. 2025 Mar 7;28(4):112166. doi: 10.1016/j.isci.2025.112166 (PMC11978348; doi:10.1016/j.isci.2025.112166)
Supplement: Document S1. Figures S1–S34 and Tables S1–S4 [file mmc1.pdf]

iScience, Volume *28*

Supplemental information

Spatial transcriptomics delineates potential differences in intestinal phenotypes of cardiac and classical necrotizing enterocolitis

Kathryn Y. Burge, Constantin Georgescu, Hua Zhong, Adam P. Wilson, Aarthi Gunasekaran, Zhongxin Yu, Addison Franca, Jeffrey V. Eckert, Jonathan D. Wren, and Hala Chaaban

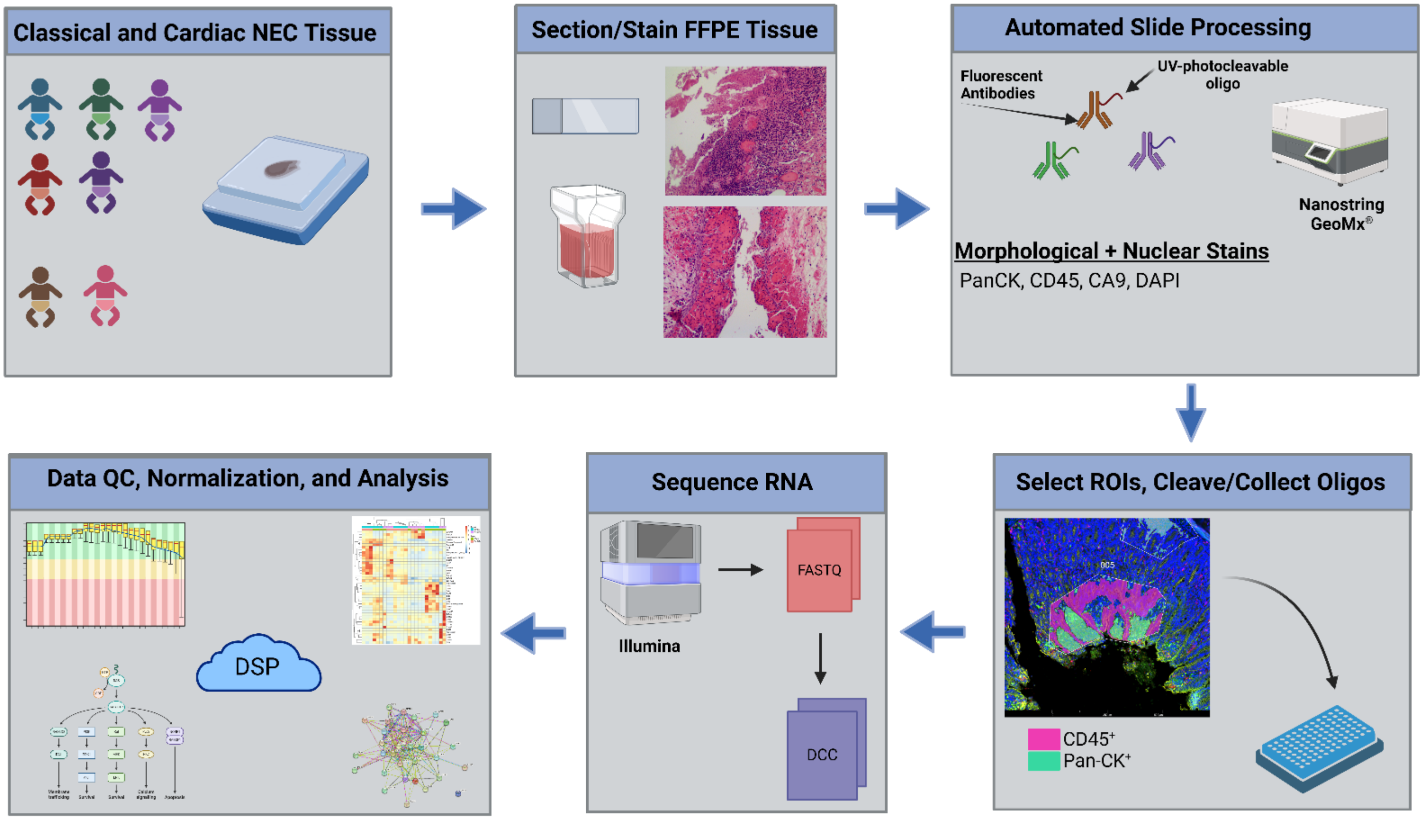

Figure S1. GeoMx® Digital Spatial Profiling (DSP) workflow, related to STAR Methods and Figure 1. Archived FFPE ileal tissues were obtained for cardiac (*n* = 2) and classical (*n* = 5) NEC. Tissue blocks were sectioned, stained with H&E, and scanned with a slide scanner. To morphologically segregate epithelial (panCK)- and immune (CD45)-rich regions, sequential tissue sections were stained with anti-panCK and anti-CD45 fluorescent antibodies bound to UV-photocleavable oligos. Four tissue sections were also stained with anti-CA9 to identify hypoxic tissue, but information gleaned from CA9 staining was limited, and this step was omitted in latter analyses. ROIs, approximately 6 per patient sample, were delineated based on H&E and fluorescent staining, and morphology masks were digitally applied to ROIs to direct UV light. Oligos within the ROIs, containing unique barcodes for over 18,000 gene targets, were cleaved by UV light and collected into individual wells of a 96-well plate. The contents of all wells were then sequenced via Illumina NGS. FASTQ sequencing files of probe counts were converted to spatially referenced DCC files via the GeoMx® NGS Pipeline, and subsequently uploaded to GeoMx® DSP data center for normalization. Abbreviations: NEC: necrotizing enterocolitis; FFPE: formalin-fixed, paraffin-embedded; UV: ultraviolet; panCK: pan-cytokeratin; CA9: carbonic anhydrase 9; DAPI: 4’,6-diamidino-2-phenylinidole; ROIs: regions of interest; RNA: ribonucleic acid; DCC: digital count conversion; QC: quality control; DSP: Digital Spatial Profiling; H&E: hematoxylin and eosin; NGS: next-generation sequencing.

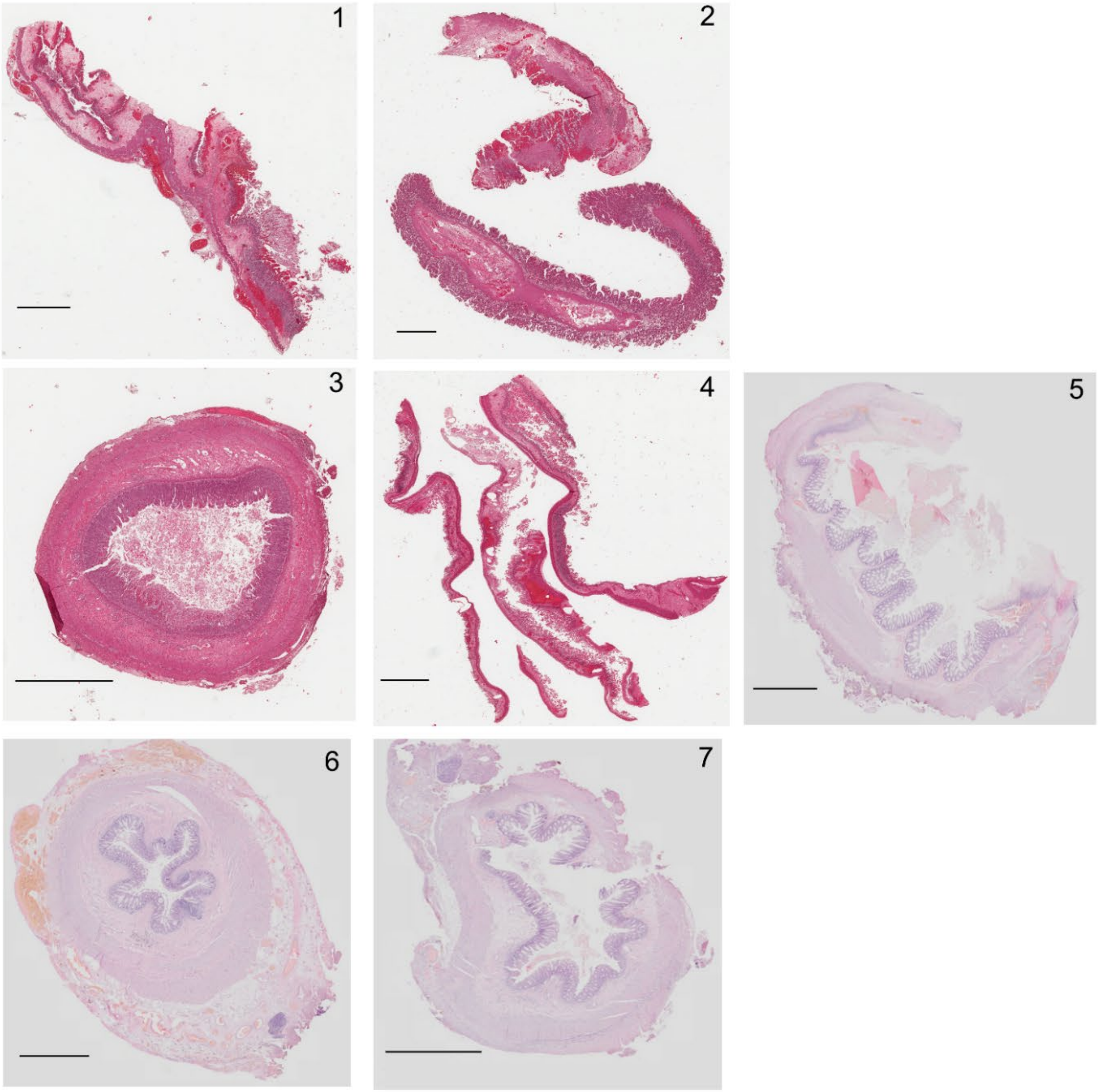

Figure S2. H&E slide scanner images of resected patient ileum, related to Figure 1. Scale bars = 2 mm. Panels 1-2: Cardiac NEC; Panels 3-7: Classical NEC. Abbreviations: H&E: hematoxylin and eosin; NEC: necrotizing enterocolitis.

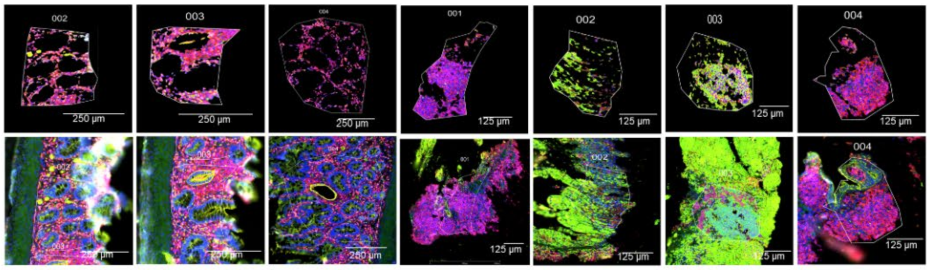

Figure S3. Immunofluorescent staining for cardiac NEC CD45⁺ ROIs, related to Figure 1. CD45 (magenta), PanCK (green), and DAPI (blue). Scale bars = 125-250 µm. Abbreviations: NEC: necrotizing enterocolitis; ROIs: regions of interest; PanCK: pancytokeratin; DAPI: 4',6-diamidino-2-phenylinidole.

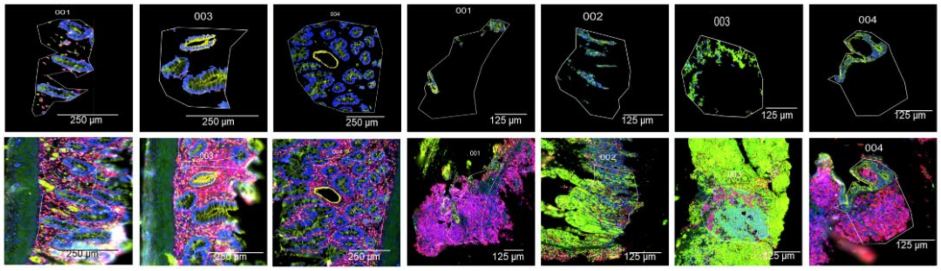

Figure S4. Immunofluorescent staining for cardiac NEC PanCK$^+$ ROIs, related to Figure 1. CD45 (magenta), PanCK (green), and DAPI (blue). Scale bars = 125-250 μm. Abbreviations: NEC: necrotizing enterocolitis; PanCK: pancytokeratin; ROIs: regions of interest; DAPI: 4',6-diamidino-2-phenylinidole.

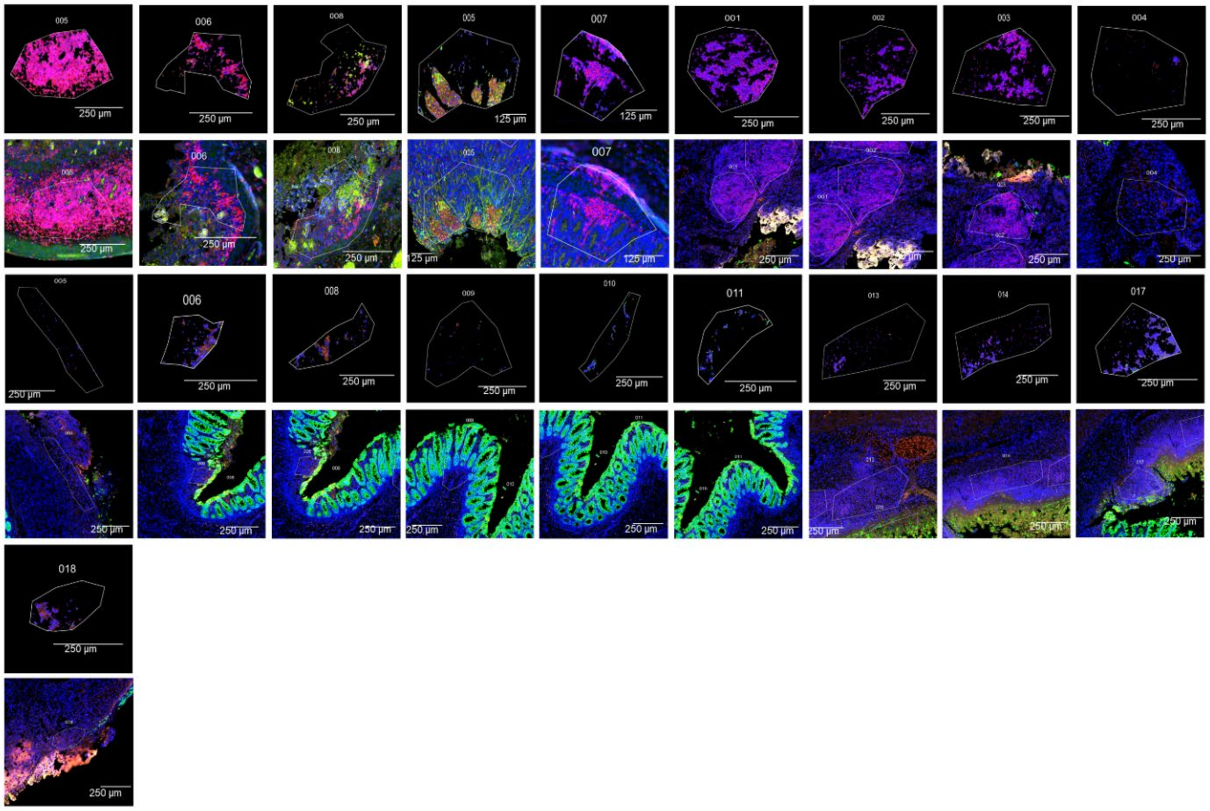

Figure S5. Immunofluorescent staining for classical NEC CD45$^+$ ROIs, related to Figure 1. CD45 (magenta), PanCK (green), and DAPI (blue). Scale bars = 125-250 µm. Abbreviations: NEC: necrotizing enterocolitis; ROIs: regions of interest; PanCK: pancytokeratin; DAPI: 4’,6-diamidino-2-phenylinidole.

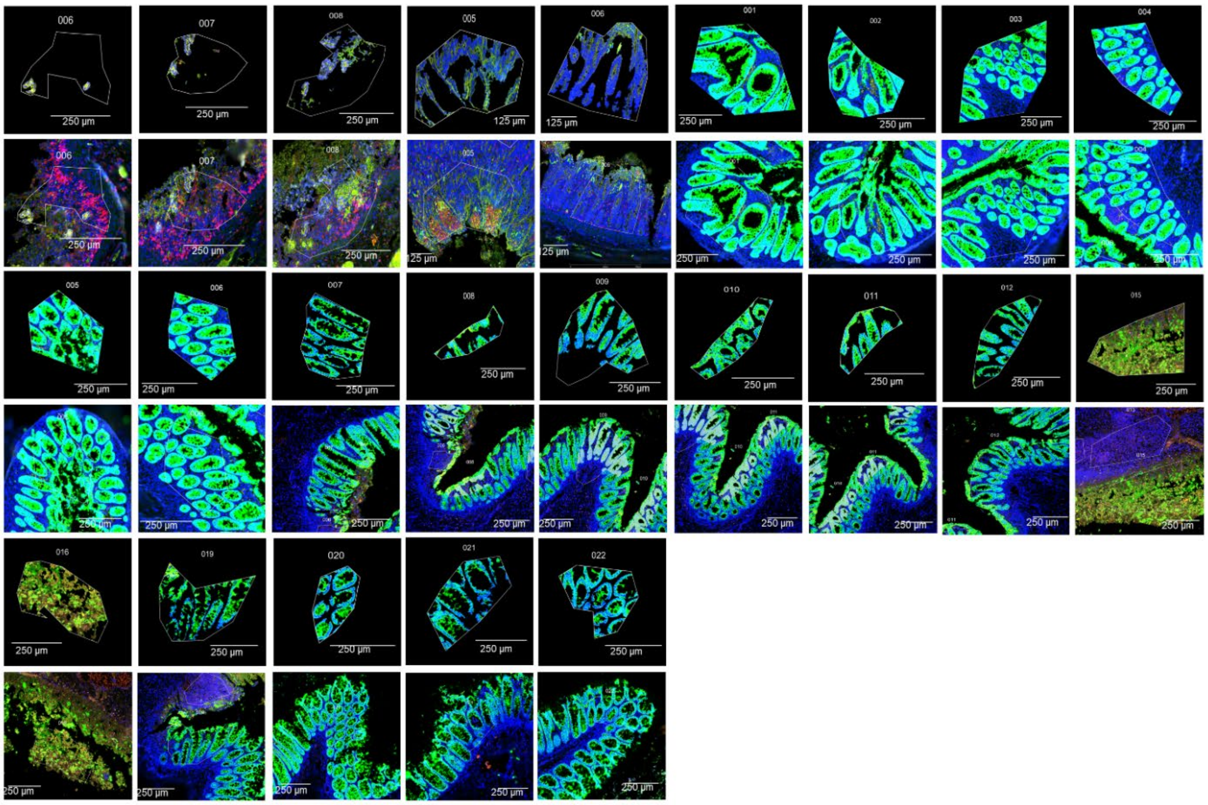

Figure S6. Immunofluorescent staining for classical NEC PanCK⁺ ROIs, related to Figure 1. CD45 (magenta), PanCK (green), and DAPI (blue). Scale bars = 125-250 µm. Abbreviations: NEC: necrotizing enterocolitis; PanCK: pancytokeratin; ROIs: regions of interest; DAPI: 4’,6-diamidino-2-phenylinidole.

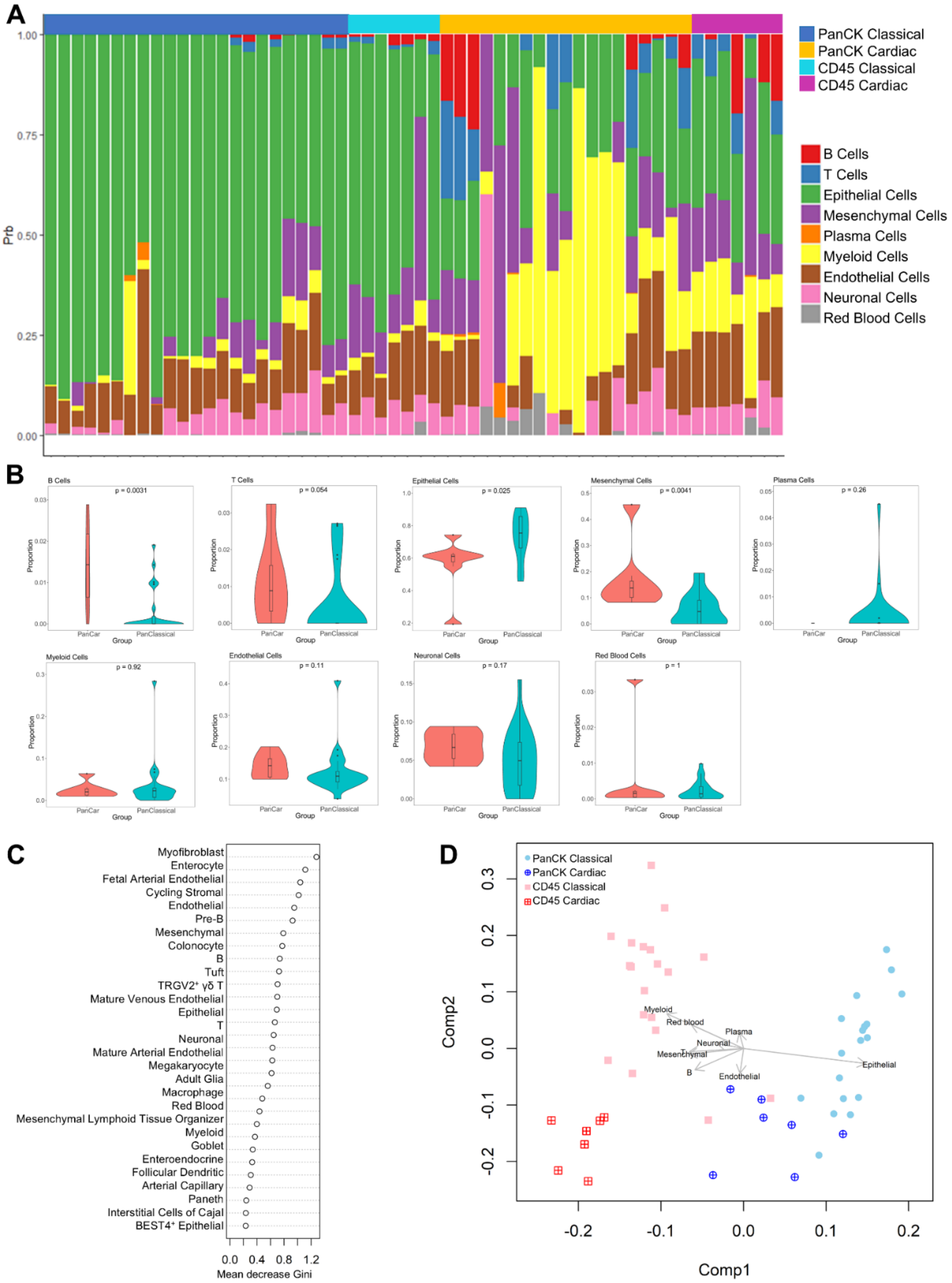

Figure S7. Cell deconvolution using adult gut HCA main cell types, related to Figure 2.
(A) Estimated main cell-type composition per ROI (adult gut HCA reference; see **STAR Methods**).
(B) Violin plots indicating main cell-type compositions across cardiac and classical NEC ROIs, denoted in (A). Middle line represents the median, box represents the interquartile range.
(C) Forest plot indicating variable importance of top cell types in discriminating between classical and cardiac NEC, scored by mean decrease in Gini.
(D) Correspondence analysis biplot indicating relative magnitude of cell-type contributions (arrows) to ROIs differentiated by NEC subtype and cell-type segment. Classical NEC epithelium: light blue, cardiac NEC epithelium: dark blue, classical NEC immune: pink; cardiac NEC immune: red.
Abbreviations: PanCK: pancytokeratin; HCA: Human Cell Atlas; ROI: region of interest; NEC: necrotizing enterocolitis; TRGV2: T cell receptor gamma variable 2; BEST4: bestrophin 4.

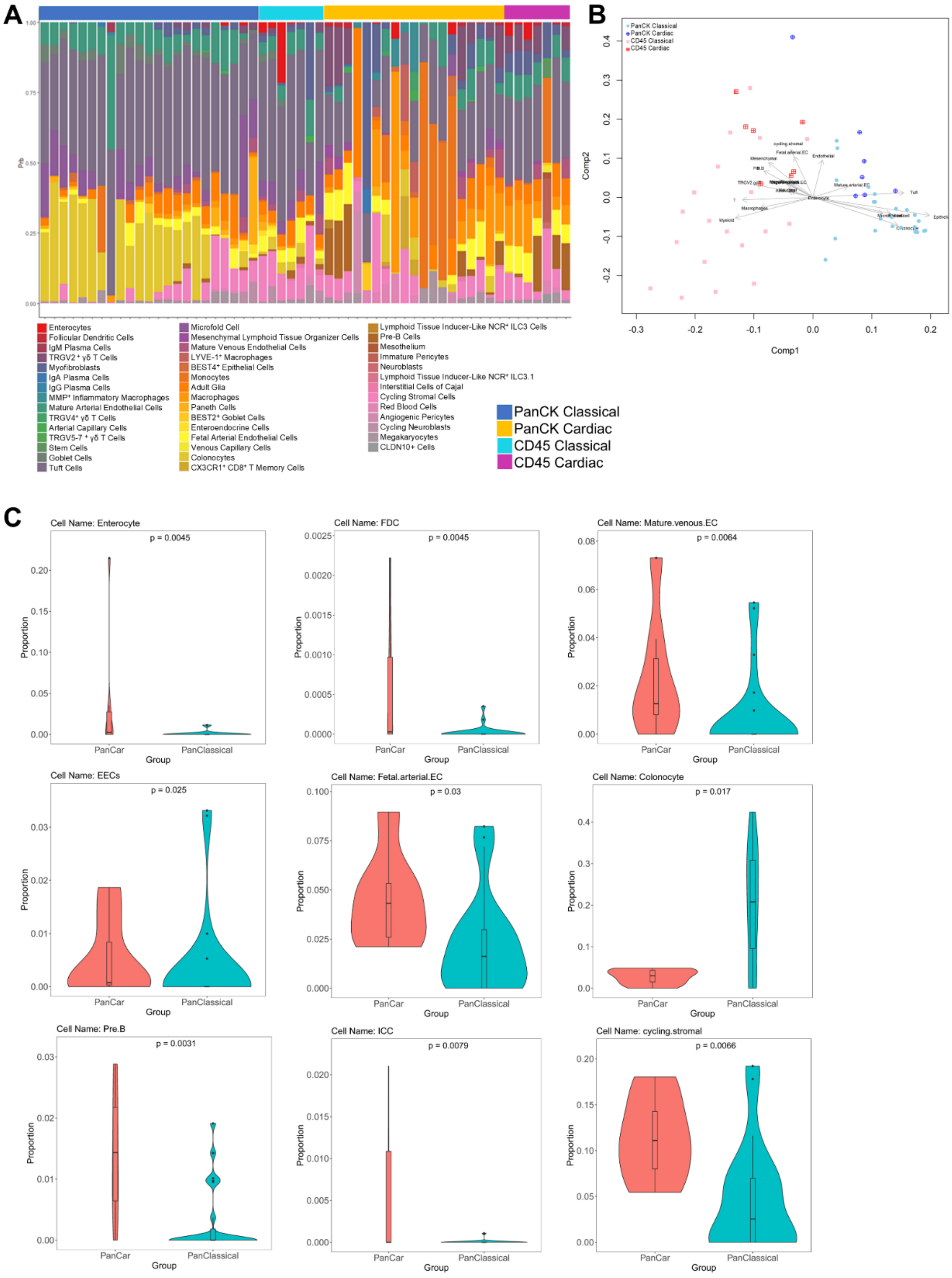

Figure S8. Cell deconvolution using adult gut HCA granular cell types, related to Figure 2.

(A) Estimated granular cell-type composition per ROI (adult gut HCA reference; see **STAR Methods**).

(B) Correspondence analysis biplot indicating relative magnitude of cell-type contributions (arrows) to ROIs differentiated by NEC subtype and cell-type segment. Classical NEC epithelium: light blue, cardiac NEC epithelium: dark blue, classical NEC immune: pink; cardiac NEC immune: red.

(C) Violin plots indicating significant ($p < 0.05$) differences in granular cell-type compositions across cardiac and classical NEC ROIs, as denoted in (A). Middle line represents the median, box represents the interquartile range.

Abbreviations: PanCK: pancytokeratin; HCA: Human Cell Atlas; ROI: region of interest; NEC: necrotizing enterocolitis; TRGV2: T cell receptor gamma variable 2; MMP: matrix metalloproteinase; LYVE: lymphatic vessel endothelial hyaluronan receptor; BEST4: bestrophin 4; CX3CR1: C-X3-C motif chemokine receptor 1; NCR: natural cytotoxicity triggering receptor 1; ILC3: innate lymphoid cells type 3; CLDN10: claudin 10.

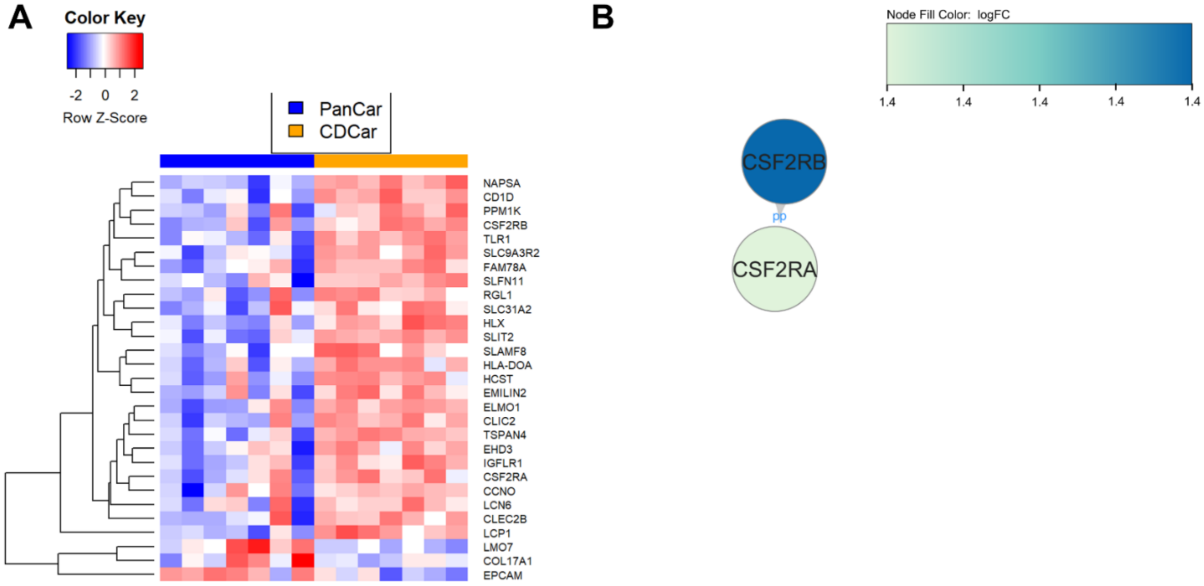

Figure S9. Cardiac NEC CD45^{+} and PanCK^{+} transcriptional differences (cell-type abundance-independent DEGs), related to STAR Methods.

(A) Heatmap of Z-scored normalized expression of DEGs (FDR-adjusted $p < 0.05$).

(B) Largest PPI subnetwork determined by STRING analysis in CytoScape. The limited scope of this network at the indicated FDR-adjusted threshold prevented further functional analysis. Only one pathway was identified in IPA with an estimated Z-score (Immunoregulatory interactions between a lymphoid and a non-lymphoid cell; gene overlap: 4; -log(p-value) 4.07; Z-score: 1; p = 8.43E-05).

Abbreviations: NEC: necrotizing enterocolitis PanCK: pancytokeratin; DEGs: differentially expressed genes; FDR: false discovery rate; PPI: protein-protein interaction; STRING: Search tool for the retrieval of interacting genes/proteins; IPA: Ingenuity Pathway Analysis.

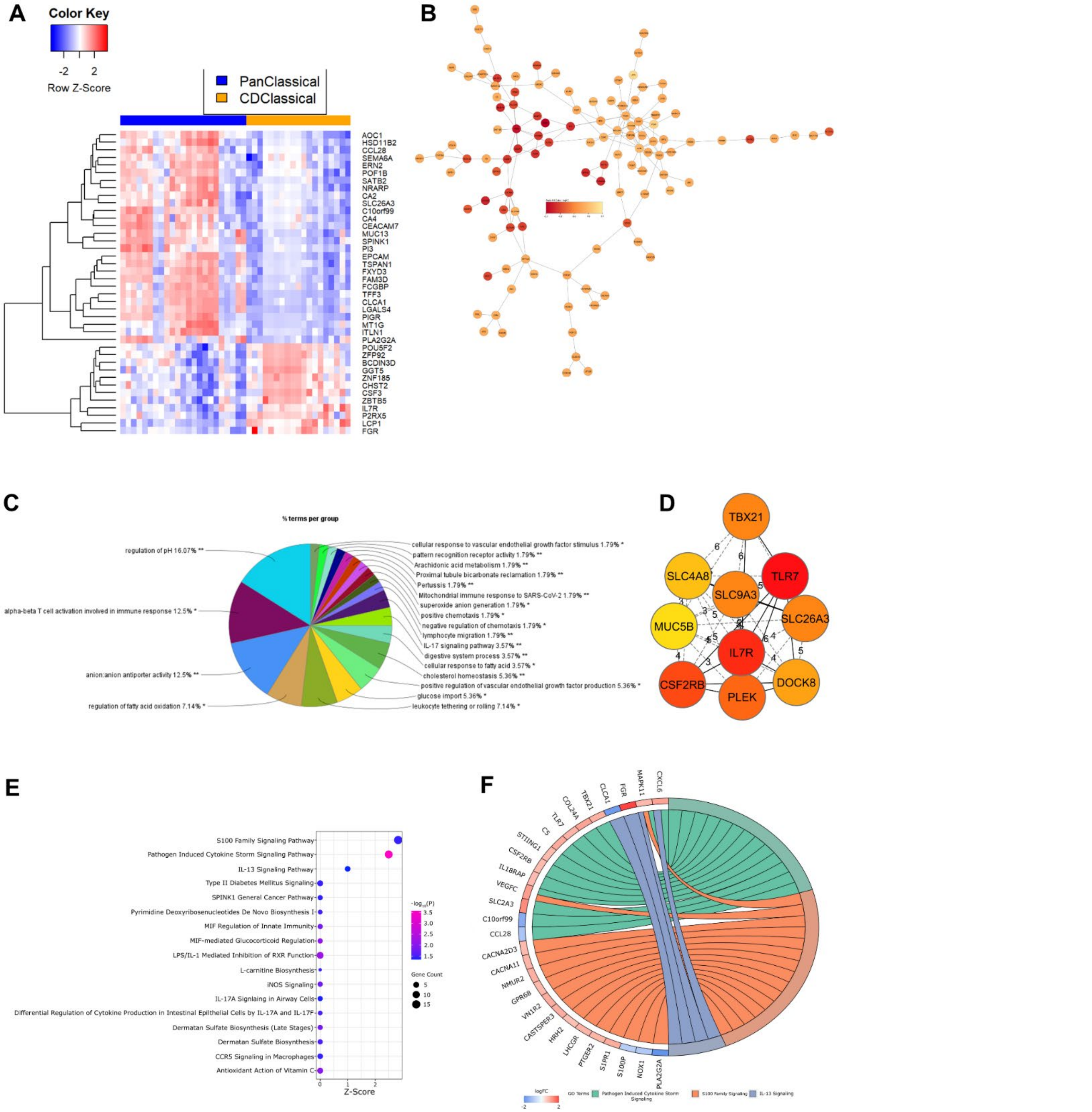

Figure S10. Classical NEC CD45$^+$ and PanCK$^+$ transcriptional differences (cell-type abundance-independent DEGs), related to STAR Methods.

(A) Heatmap of Z-scored normalized expression of the top 40 DEGs (FDR-adjusted $p < 0.01$).

(B) Largest PPI subnetwork determined by STRING analysis in CytoScape.

(C) ClueGO pie chart of GO term functional enrichment (% terms/group); *adjusted $p < 0.05$, **adjusted $p < 0.01$.

(D) Top 10 hub genes identified through CytoHubba (red = most connections).

(E) Bubble plot of IPA-defined differentially enriched pathways. Bubble size correlates with gene overlap (≥2), color indicates significance ($-\log_{10}(P)$) of pathway enrichment, and X-axis represents Z-score.

(F) Chord diagram illustrating composition of 3 differentially regulated pathways (by Z-score and gene count) annotated in (E). Pathways are shown on the right and gene fold change is indicated on the left. Chords connecting genes to pathways indicate gene inclusion within the pathway.

Abbreviations: NEC: necrotizing enterocolitis; PanCK: pancytokeratin; DEGs: differentially expressed genes; FDR: false discovery rate; PPI: protein-protein interaction; STRING: search tool for the retrieval of interacting genes/proteins; GO: gene ontology; IPA: Ingenuity Pathway Analysis.

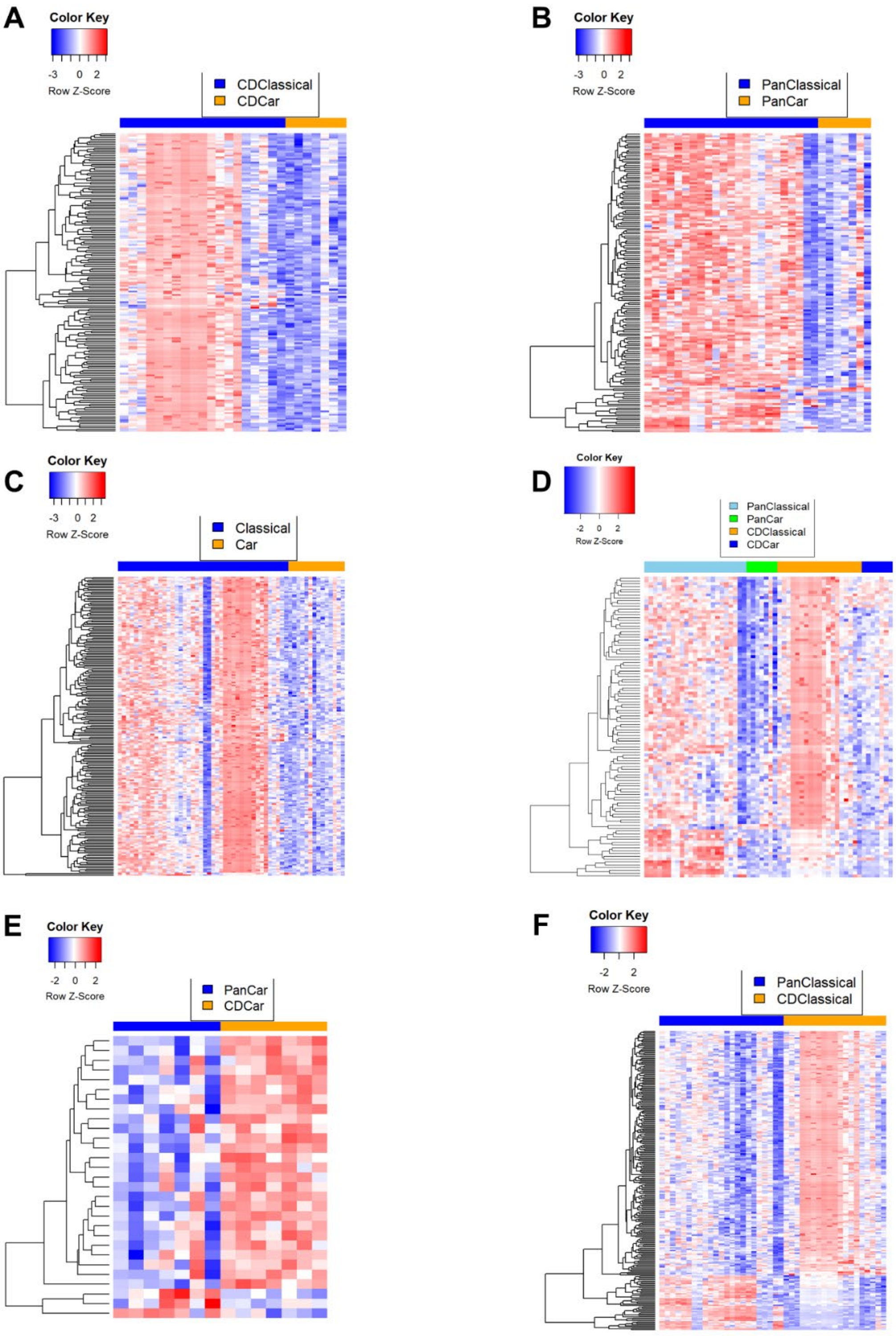

Figure S11. Comprehensive heatmaps of Z-scored normalized expression of DEGs in cell-type abundance-independent analyses, related to Figures 3-6.

(A) Cardiac and classical NEC CD45$^+$ ROI analysis (FDR-adjusted p < 0.01).
(B) Cardiac and classical NEC PanCK$^+$ ROI analysis (FDR-adjusted p < 0.01).
(C) Cardiac and classical NEC cell-type segment directionally concordant analysis (FDR-adjusted p < 0.15).
(D) Cardiac and classical NEC interaction analysis (FDR-adjusted p < 0.05).
(E) Cardiac NEC CD45$^+$ and PanCK$^+$ ROI analysis (FDR-adjusted p < 0.05).
(F) Classical NEC CD45$^+$ and PanCK$^+$ ROI analysis (FDR-adjusted p < 0.01).

Abbreviations: DEGs: differentially expressed genes; NEC: necrotizing enterocolitis; ROI: region of interest; PanCK: pancytokeratin.

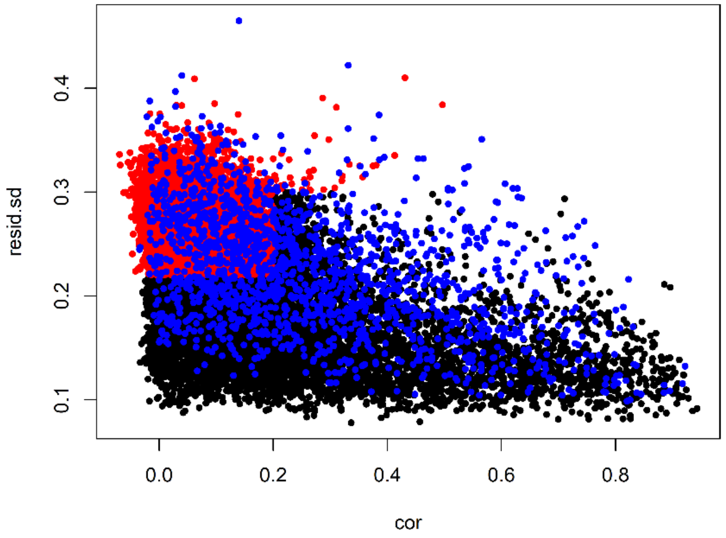

Figure S12. Reverse deconvolution generates cell-type-independent genes for downstream network analysis, related to STAR Methods. Correlation (x-axis) and residual standard deviation (y-axis) indicate the degree to which gene expression variance is dependent upon cell-type abundance within ROIs. Non-DEGs are represented in black and red. Genes with low correlation and high residual standard deviation (red) are likely genes with cell-type abundance-independent expression, while genes with low residual standard deviation (black) indicate cell-type abundance-dependent differences. The full suite of DEGs (blue) contains genes with expressions dependent and independent upon cell-type abundance, as well as genes with unexplained variance. DEGs with low correlation and high residual standard deviation (overlaying red genes) indicate genes with genuine, cell-type abundance-independent expressions, while DEGs with low residual standard deviation (overlaying black genes) indicate expression differences driven by cell-type abundance differences.

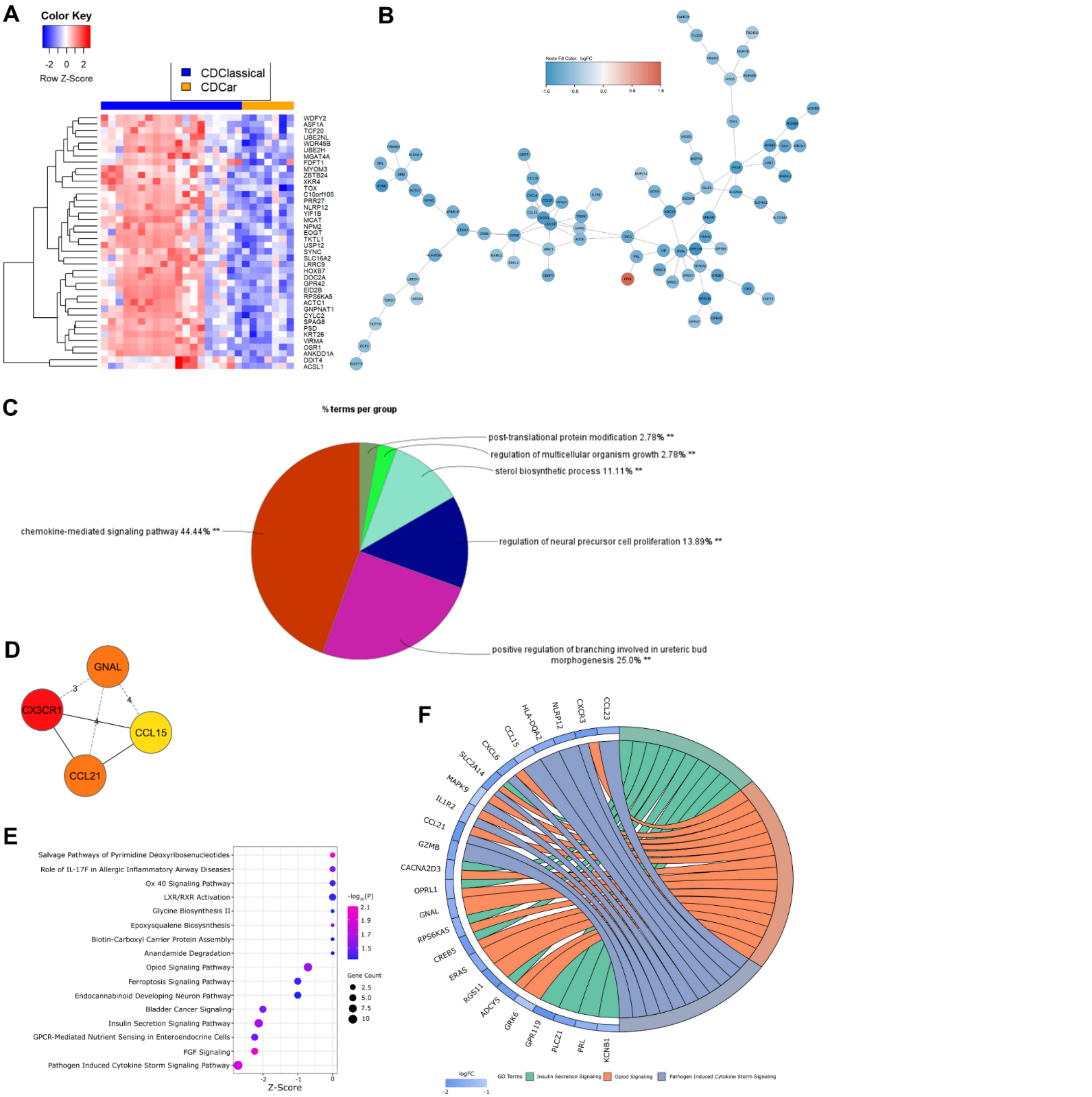

Figure S13. Cardiac and classical NEC CD45⁺ ROI transcriptional differences (comprehensive DEGs), related to Figure 3.

(A) Heatmap of Z-scored normalized expression of the top 40 DEGs (FDR-adjusted $p < 0.15$).

(B) Largest PPI subnetwork determined by STRING analysis in CytoScape.

(C) ClueGO pie chart of GO term functional enrichment (% terms/group); **adjusted $p < 0.01$.

(D) Top 4 hub genes identified through CytoHubba (red = strongest associations).

(E) Bubble plot of IPA-defined differentially enriched pathways. Bubble size correlates with gene overlap (≥2), color indicates significance ($-\log_{10}(P)$) of pathway enrichment, and X-axis represents Z-score.

(F) Chord diagram illustrating composition of 3 differentially regulated pathways (by Z-score and gene count) annotated in (E). Pathways are shown on the right and gene fold change is indicated on the left. Chords connecting genes to pathways indicate gene inclusion within the pathway.

Abbreviations: NEC: necrotizing enterocolitis; ROI: region of interest; DEGs: differentially expressed genes; FDR: false discovery rate; PPI: protein-protein interaction; STRING: search tool for the retrieval of interacting genes/proteins; GO: gene ontology; IPA: Ingenuity Pathway Analysis.

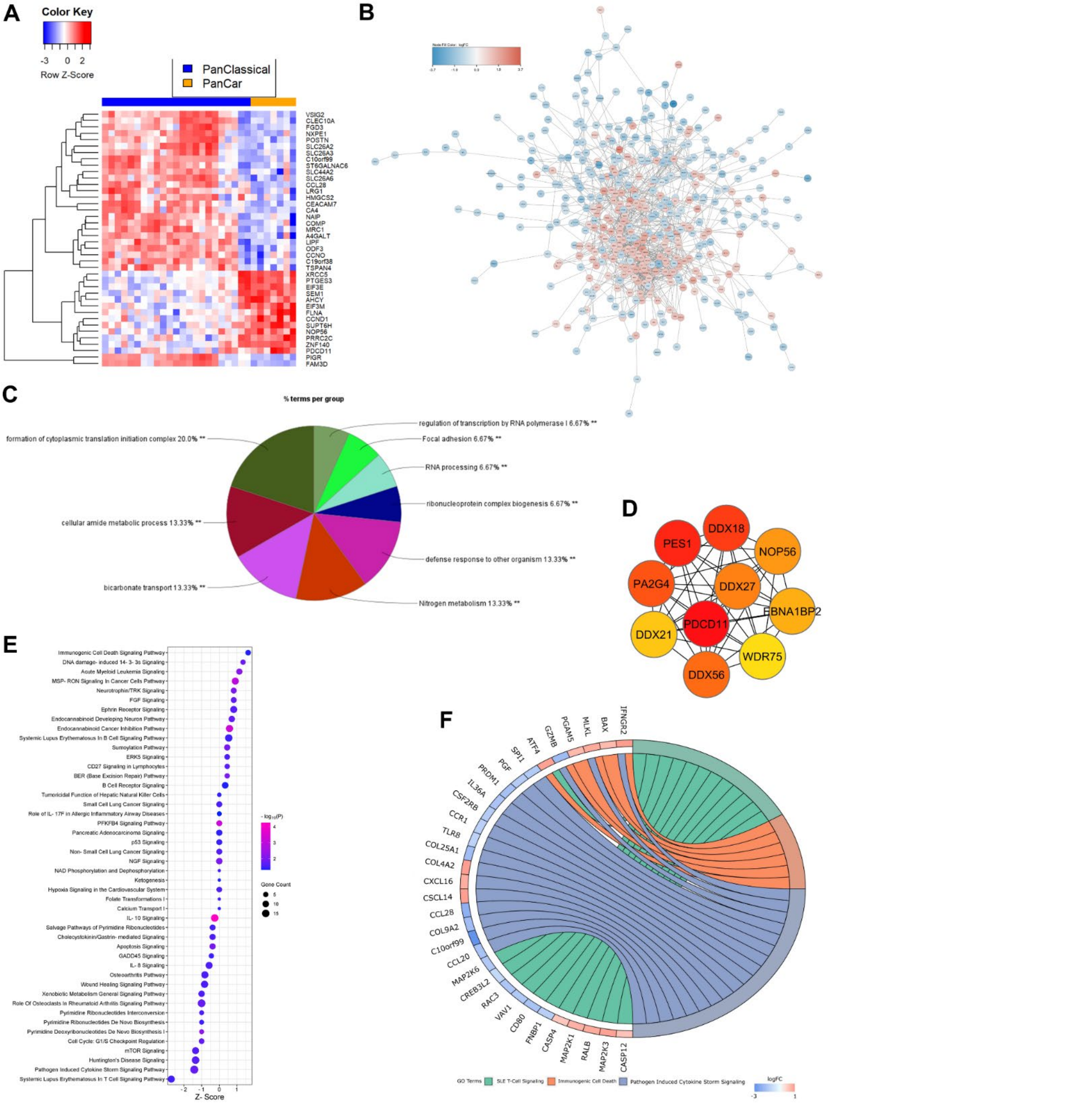

Figure S14. Cardiac and classical NEC PanCK⁺ ROI transcriptional differences (comprehensive DEGs), related to Figure 4.

(A) Heatmap of Z-scored normalized expression of the top 40 DEGs (FDR-adjusted $p < 0.15$).

(B) Largest PPI subnetwork determined by STRING analysis in CytoScape.

(C) ClueGO pie chart of GO term functional enrichment (% terms/group); **adjusted $p < 0.01$.

(D) Top 10 hub genes identified through CytoHubba (red = strongest associations).

(E) Bubble plot of IPA-defined differentially enriched pathways. Bubble size correlates with gene overlap (≥2), color indicates significance ($-\log_{10}(P)$) of pathway enrichment, and X-axis represents Z-score.

(F) Chord diagram illustrating composition of 3 differentially regulated pathways (by Z-score and gene count) annotated in (E). Pathways are shown on the right and gene fold change is indicated on the left. Chords connecting genes to pathways indicate gene inclusion within the pathway.

Abbreviations: NEC: necrotizing enterocolitis; PanCK: pancytokeratin; ROI: region of interest; DEGs: differentially expressed genes; FDR: false discovery rate; PPI: protein-protein interaction; STRING: search tool for the retrieval of interacting genes/proteins; GO: gene ontology; IPA: Ingenuity Pathway Analysis.

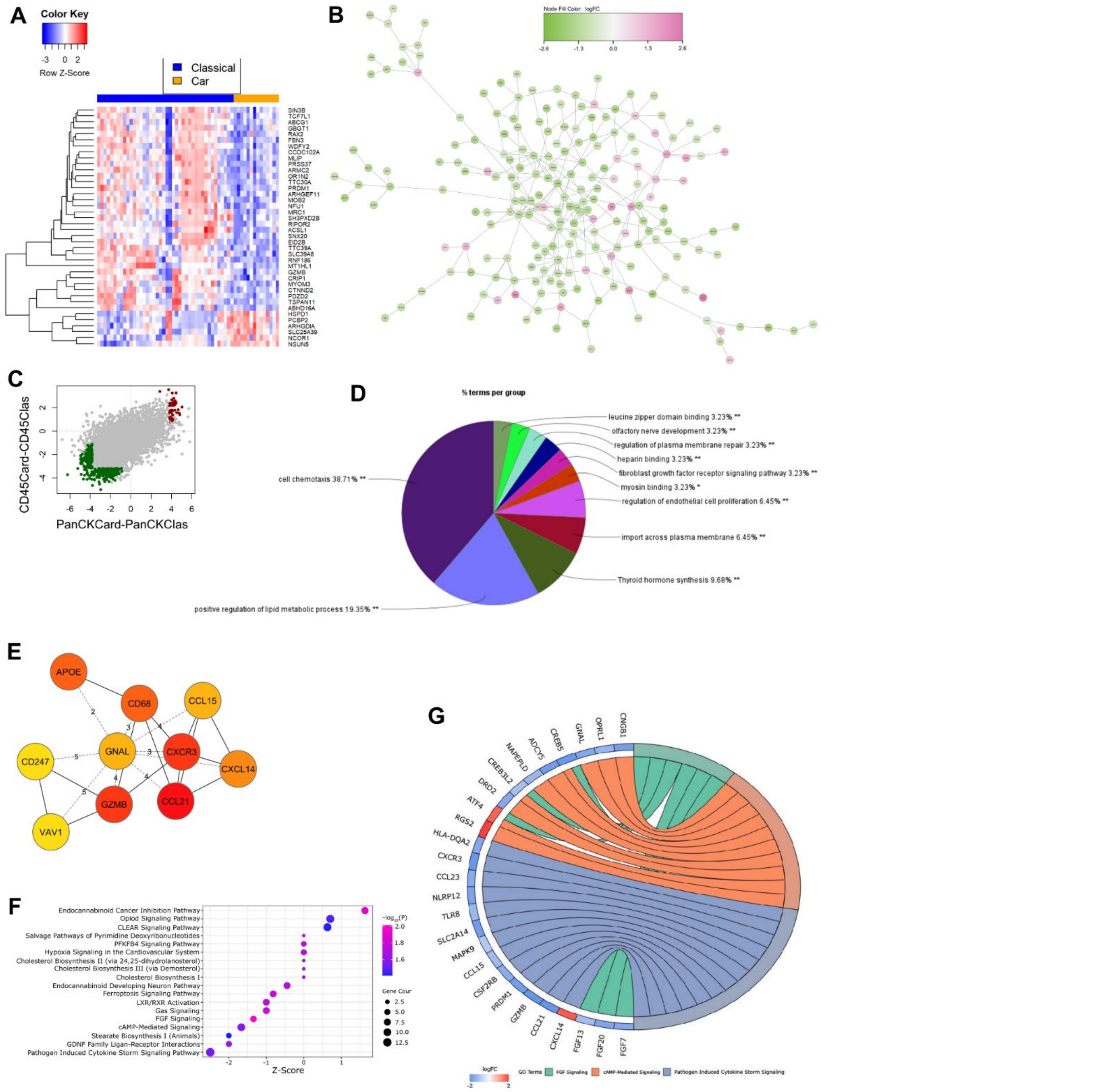

Figure S15. Cell-type segment directionally concordant differences in cardiac and classical NEC (comprehensive DEGs), related to Figure 5.

(A) Heatmap of Z-scored normalized expression of the top 40 DEGs (FDR-adjusted $p < 0.15$).

(B) Largest PPI subnetwork determined by STRING analysis in CytoScape.

(C) Scatterplot of genes expressed differentially between NEC subtypes but in same direction within cell-type segments.

(D) ClueGO pie chart of GO term functional enrichment (% terms/group); *adjusted $p < 0.05$; **adjusted $p < 0.01$.

(E) Top 10 hub genes identified through CytoHubba (red = strongest associations).

(F) Bubble plot of IPA-defined differentially enriched pathways. Bubble size correlates with gene overlap (≥2), color indicates significance ($-\log_{10}(P)$) of pathway enrichment, and X-axis represents Z-score.

(G) Chord diagram illustrating composition of 3 differentially regulated pathways (by Z-score and gene count) annotated in (F). Pathways are shown on the right and gene fold change is indicated on the left. Chords connecting genes to pathways indicate gene inclusion within the pathway.

Abbreviations: NEC: necrotizing enterocolitis; DEGs: differentially expressed genes; FDR: false discovery rate; PPI: protein-protein interaction; STRING: search tool for the retrieval of interacting genes/proteins; GO: gene ontology; IPA: Ingenuity Pathway Analysis.

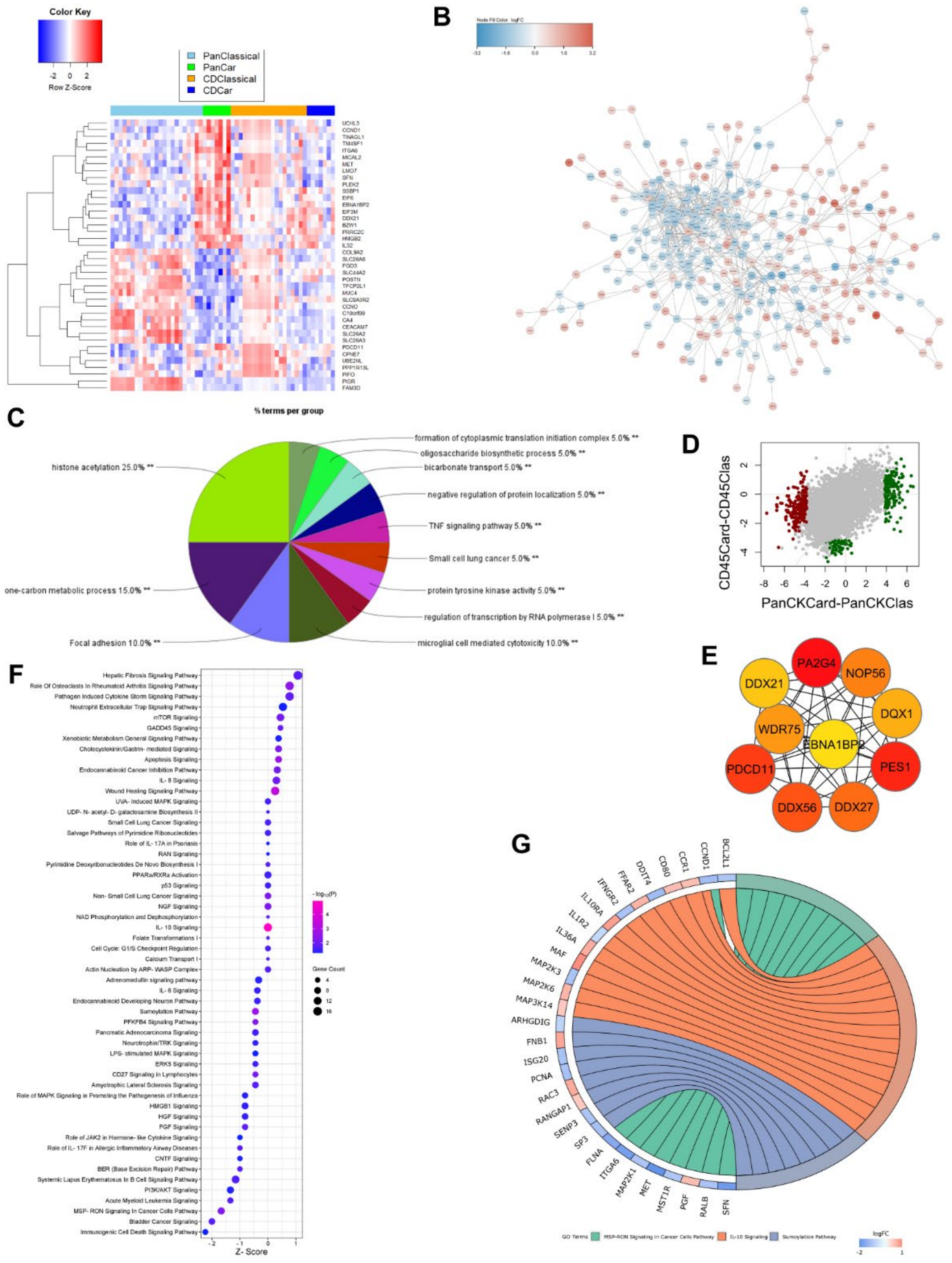

Figure S16. Cardiac and classical NEC transcriptional differences (interaction, comprehensive DEGs), related to Figure 6.

(A) Heatmap of Z-scored normalized expression of the top 40 DEGs (FDR-adjusted $p < 0.15$).

(B) Largest PPI subnetwork determined by STRING analysis in CytoScape.

(C) ClueGO pie chart of GO term functional enrichment (% terms/group); **adjusted $p < 0.01$.

(D) Scatterplot of genes expressed differentially between NEC subtypes but in different direction within cell-type segments.

(E) Top 10 hub genes identified through CytoHubba (red = strongest associations).

(F) Bubble plot of IPA-defined differentially enriched pathways. Bubble size correlates with gene overlap (≥2), color indicates significance ($-\log_{10}(P)$) of pathway enrichment, and X-axis represents Z-score.

(G) Chord diagram illustrating composition of 3 differentially regulated pathways (by Z-score and gene count) annotated in (F). Pathways are shown on the right and gene fold change is indicated on the left. Chords connecting genes to pathways indicate gene inclusion within the pathway.

Abbreviations: NEC: necrotizing enterocolitis; DEGs: differentially expressed genes; FDR: false discovery rate; PPI: protein-protein interaction; STRING: search tool for the retrieval of interacting genes/proteins; GO: gene ontology; IPA: Ingenuity Pathway Analysis.

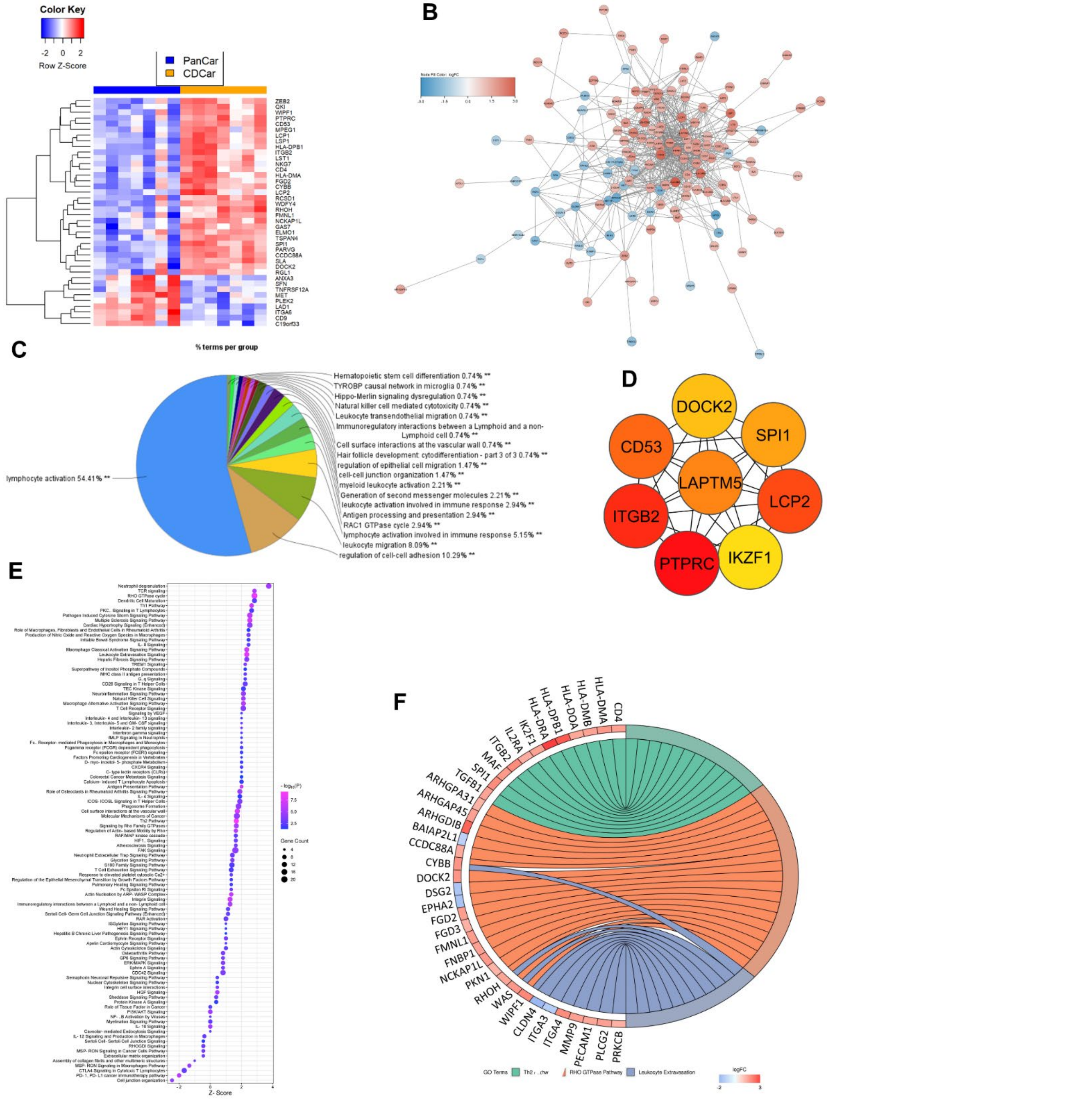

Figure S17. Cardiac NEC CD45⁺ and PanCK⁺ transcriptional differences (comprehensive DEGs), related to STAR Methods.

(A) Heatmap of Z-scored normalized expression of the top 40 DEGs (FDR-adjusted p < 0.05).

(B) Largest PPI subnetwork determined by STRING analysis in CytoScape.

(C) ClueGO pie chart of GO term functional enrichment (% terms/group); **adjusted p < 0.01.

(D) Top 8 hub genes identified through CytoHubba (red = strongest associations).

(E) Bubble plot of IPA-defined differentially enriched pathways. Bubble size correlates with gene overlap (≥2), color indicates significance ($-\log_{10}(P)$) of pathway enrichment, and X-axis represents Z-score.

(F) Chord diagram illustrating composition of 3 differentially regulated pathways (by Z-score and gene count) annotated in (E). Pathways are shown on the right and gene fold change is indicated on the left. Chords connecting genes to pathways indicate gene inclusion within the pathway.

Abbreviations: NEC: necrotizing enterocolitis; PanCK: pancytokeratin; DEGs: differentially expressed genes; FDR: false discovery rate; PPI: protein-protein interaction; STRING: search tool for the retrieval of interacting genes/proteins; GO: gene ontology; IPA: Ingenuity Pathway Analysis.

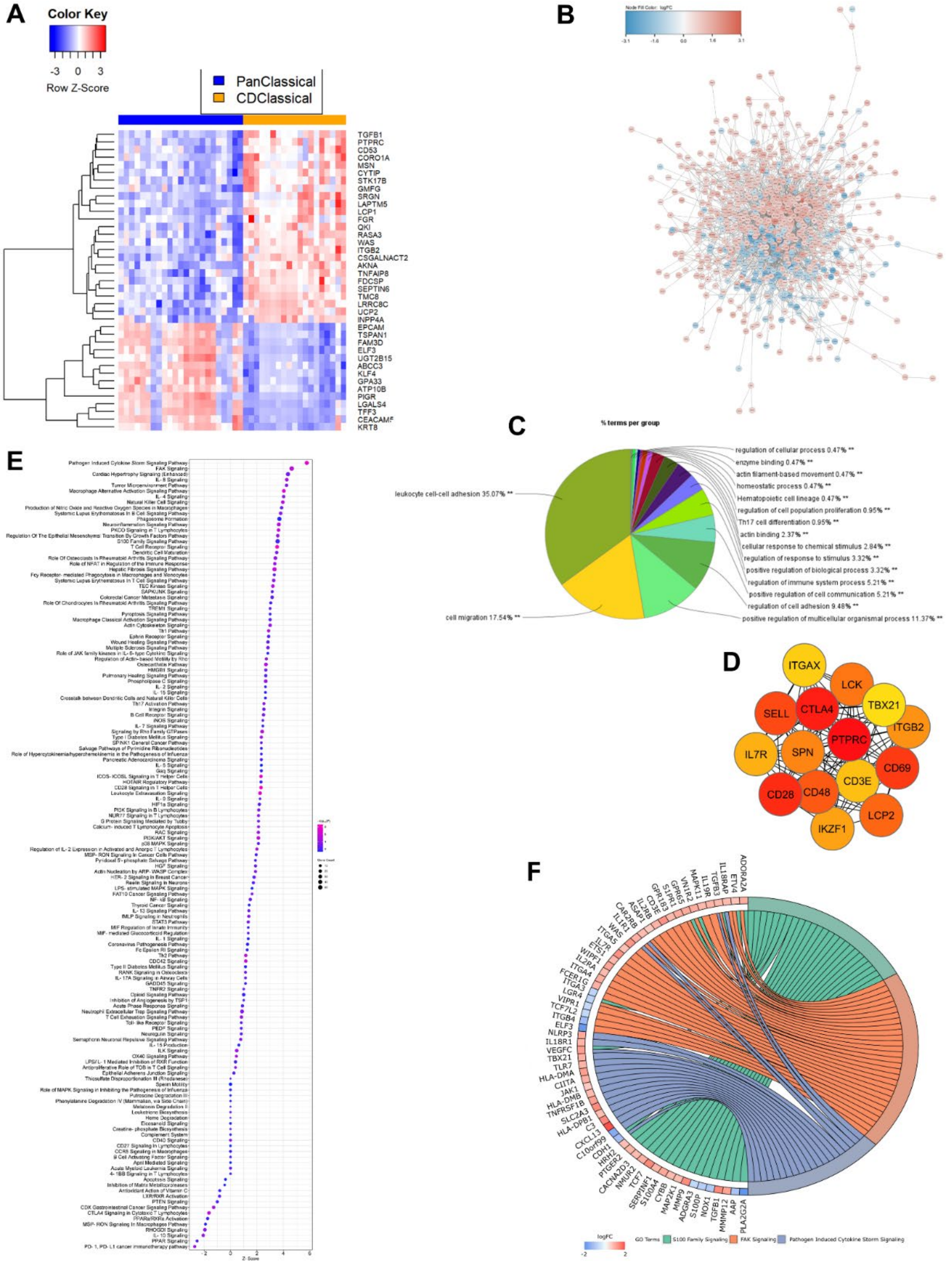

Figure S18. Classical NEC CD45⁺ and PanCK⁺ transcriptional differences (comprehensive DEGs), related to STAR Methods.

(A) Heatmap of Z-scored normalized expression of the top 40 DEGs (FDR-adjusted $p < 0.15$).

(B) Largest PPI subnetwork determined by STRING analysis in CytoScape.

(C) ClueGO pie chart of GO term functional enrichment (% terms/group); **adjusted $p < 0.01$.

(D) Top 15 hub genes identified through CytoHubba (red = strongest associations).

(E) Bubble plot of IPA-defined differentially enriched pathways. Bubble size correlates with gene overlap (≥2), color indicates significance ($-\log_{10}(P)$) of pathway enrichment, and X-axis represents Z-score.

(F) Chord diagram illustrating composition of 3 differentially regulated pathways (by Z-score and gene count) annotated in (E). Pathways are shown on the right and gene fold change is indicated on the left. Chords connecting genes to pathways indicate gene inclusion within the pathway.

Abbreviations: NEC: necrotizing enterocolitis; PanCK: pancytokeratin; DEGs: differentially expressed genes; FDR: false discovery rate; PPI: protein-protein interaction; STRING: search tool for the retrieval of interacting genes/proteins; GO: gene ontology; IPA: Ingenuity Pathway Analysis.

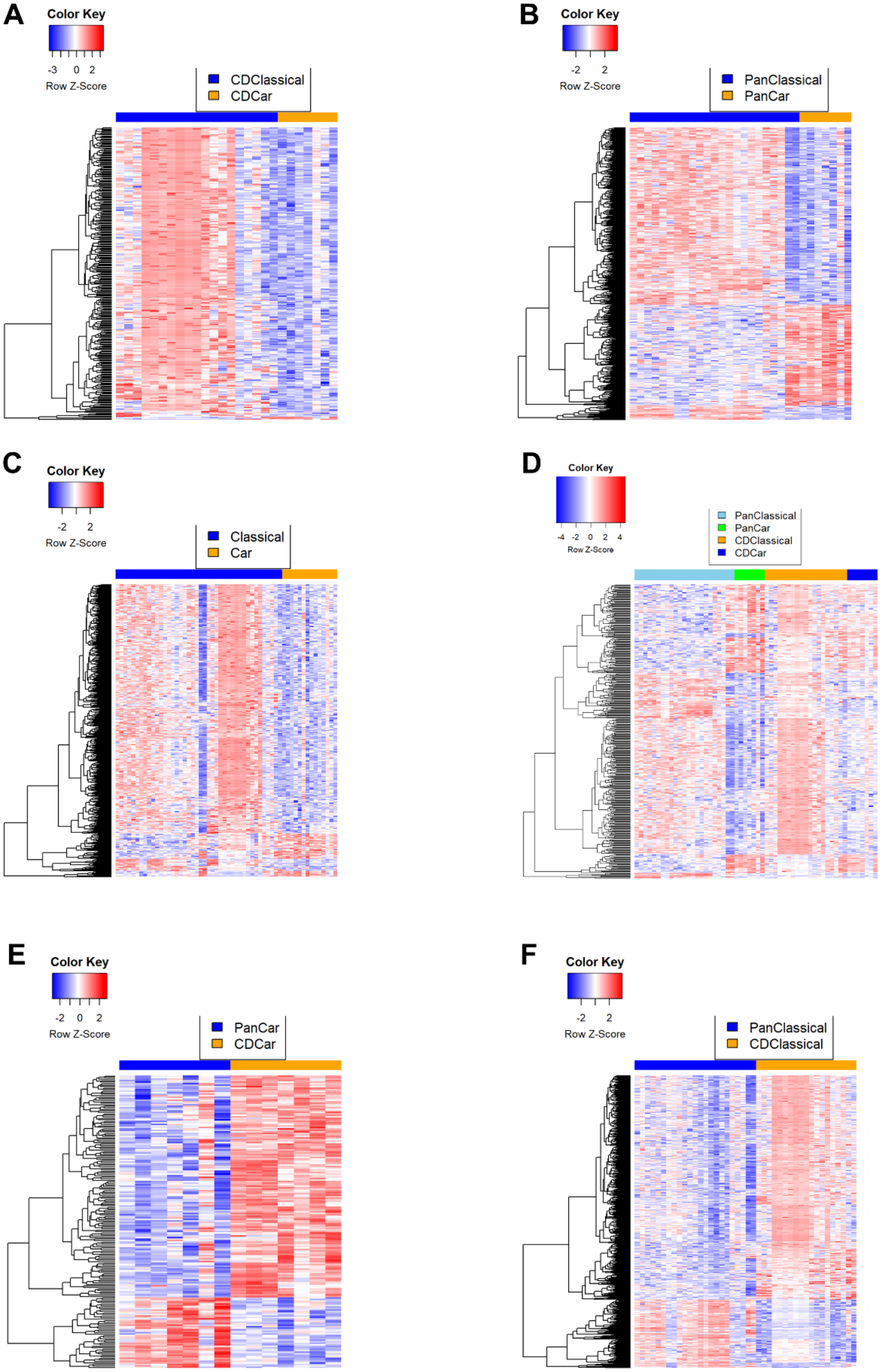

Figure S19. Comprehensive heatmaps of Z-scored normalized expression of DEGs in comprehensive analyses, related to Figures 3-6.

(A) Cardiac and classical NEC $CD45^+$ ROI analysis (FDR-adjusted $p < 0.15$).
(B) Cardiac and classical NEC $PanCK^+$ ROI analysis (FDR-adjusted $p < 0.15$).
(C) Cardiac and classical NEC cell-type segment directionally concordant analysis (FDR-adjusted $p < 0.15$).
(D) Cardiac and classical NEC interaction analysis (FDR-adjusted $p < 0.15$).
(E) Cardiac NEC $CD45^+$ and $PanCK^+$ ROI analysis (FDR-adjusted $p < 0.05$).
(F) Classical NEC $CD45^+$ and $PanCK^+$ ROI analysis (FDR-adjusted $p < 0.15$).

Abbreviations: DEGs: differentially expressed genes; NEC: necrotizing enterocolitis; ROI: region of interest; PanCK: pancytokeratin.

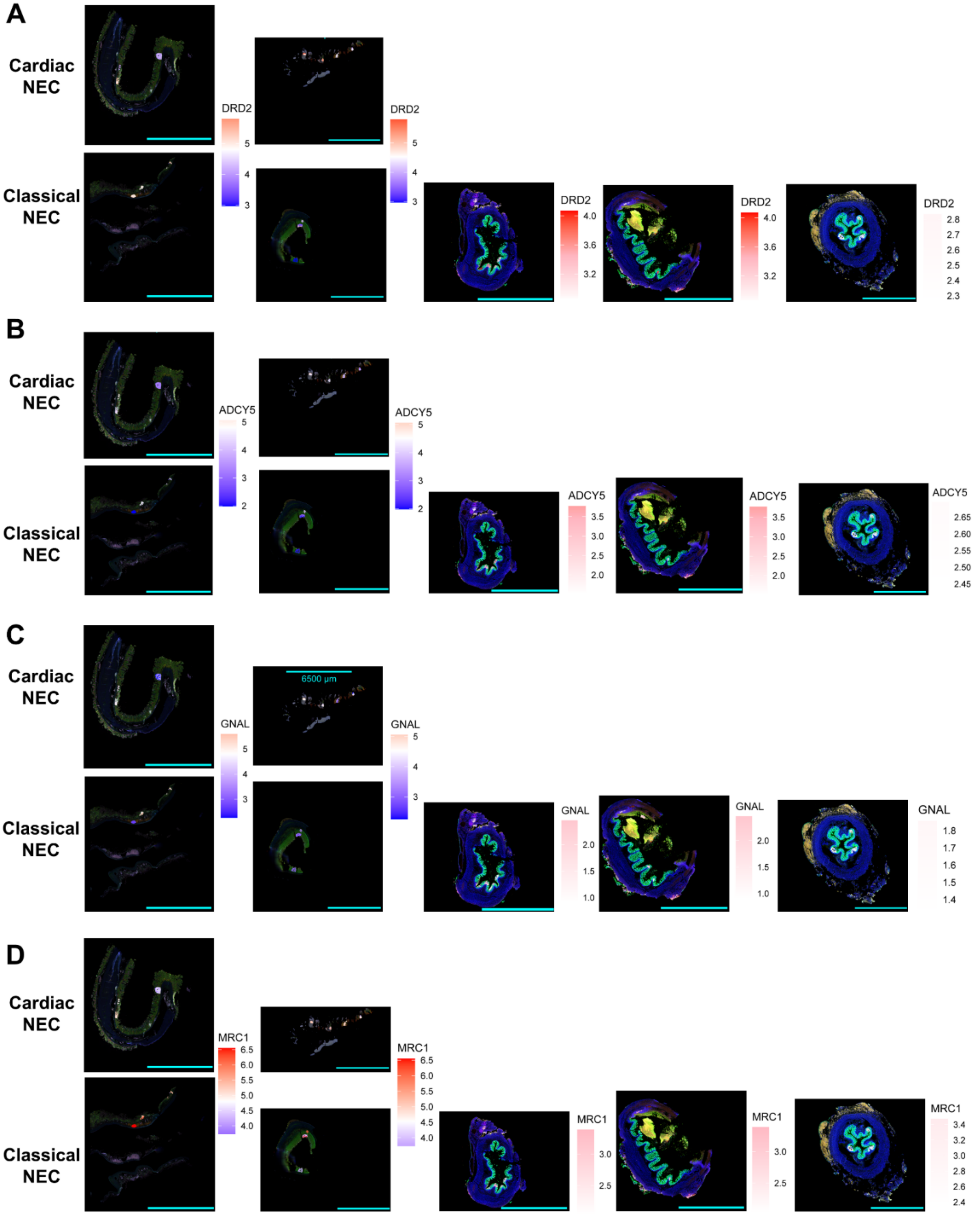

Figure S20. Spatial visualization of expression data using SpatialOmicsOverlay, related to Figures 3-4 .
(A) Cardiac and classical NEC spatial histological overlay of *DRD2* gene expression.
(B) Cardiac and classical NEC spatial histological overlay of *ADCY5* gene expression.
(C) Cardiac and classical NEC spatial histological overlay of *GNAL* gene expression.
(D) Cardiac and classical NEC spatial histological overlay of *MRC1* gene expression.
Scale bars, 6,500 µm. Abbreviations: NEC: necrotizing enterocolitis; *DRD2*: dopamine D2 receptor; *ADCY5*: adenylyl cyclase type 5; *GNAL*: G protein subunit alpha L; *MRC1*: mannose receptor C-type 1.

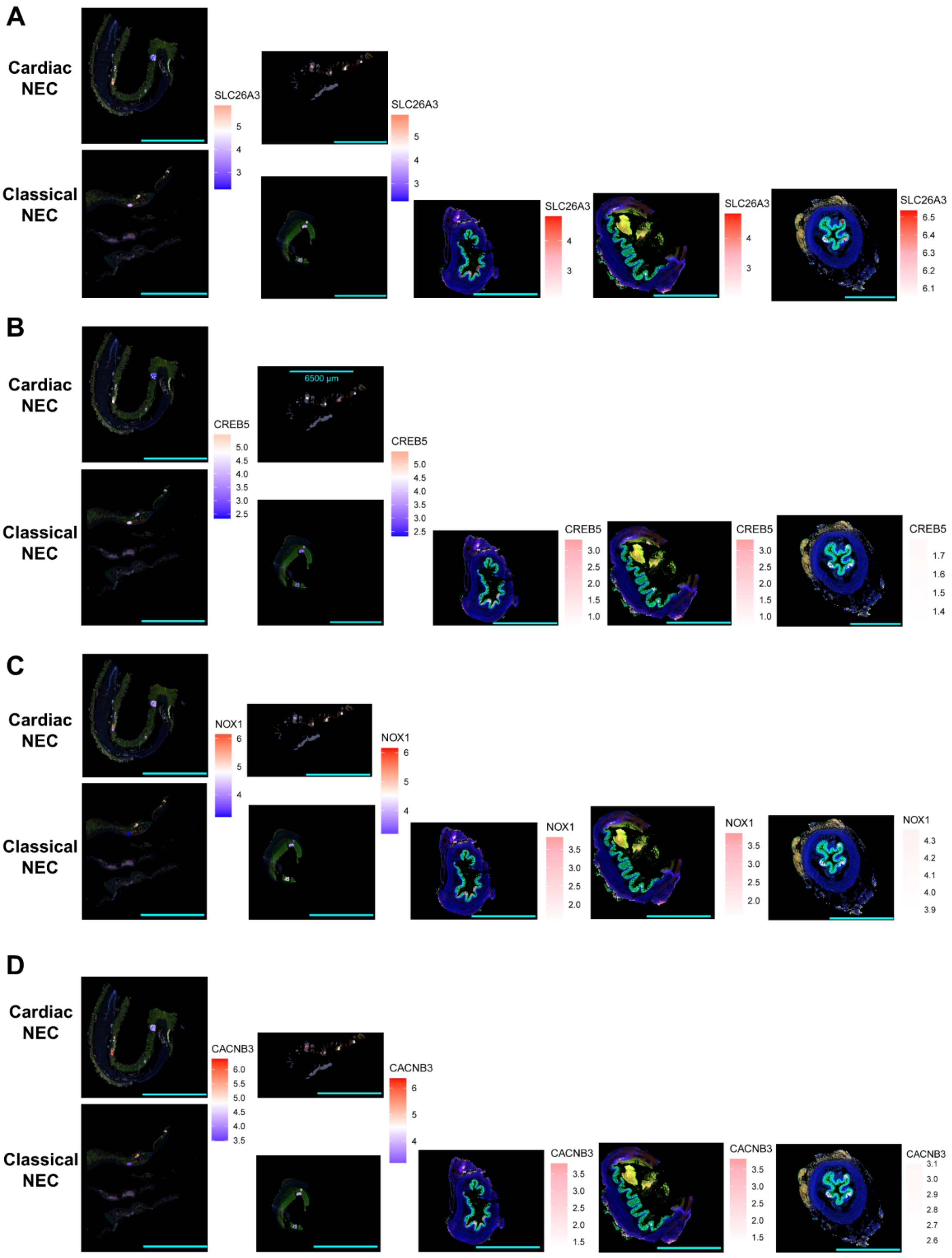

Figure S21. Spatial visualization of expression data using SpatialOmicsOverlay, related to Figures 5-6.

(A) Cardiac and classical NEC spatial histological overlay of *SLC26A3* gene expression.

(B) Cardiac and classical NEC spatial histological overlay of *CREB5* gene expression.

(C) Cardiac and classical NEC spatial histological overlay of *NOX1* gene expression.

(D) Cardiac and classical NEC spatial histological overlay of *CACNB3* gene expression.

Scale bars, 6,500 µm. Abbreviations: NEC: necrotizing enterocolitis; *SLC26A3*: solute carrier family 26 member 3; *CREB5*: cAMP responsive element binding protein 5; *NOX1*: NADPH oxidase 1; *CACNB3*: calcium voltage-gated channel auxiliary subunit beta 3.

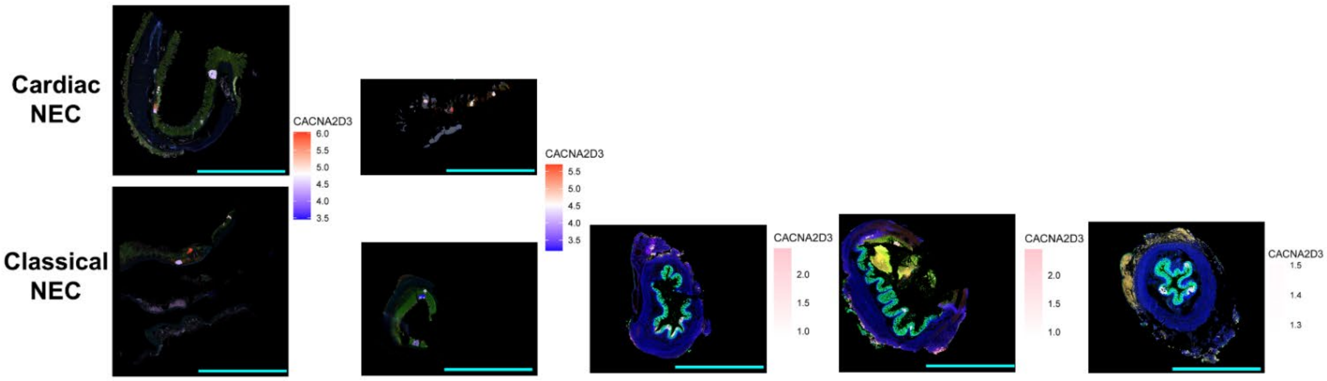

Figure S22. Spatial visualization of expression data using SpatialOmicsOverlay, related to Figure 6. Cardiac and classical NEC spatial histological overlay of *CACNA2D3* gene expression.

Scale bars, 6,500 um. Abbreviations: NEC: necrotizing enterocolitis; *CACNA2D3*: calcium voltage-gated channel auxiliary subunit alpha 2/delta 3.

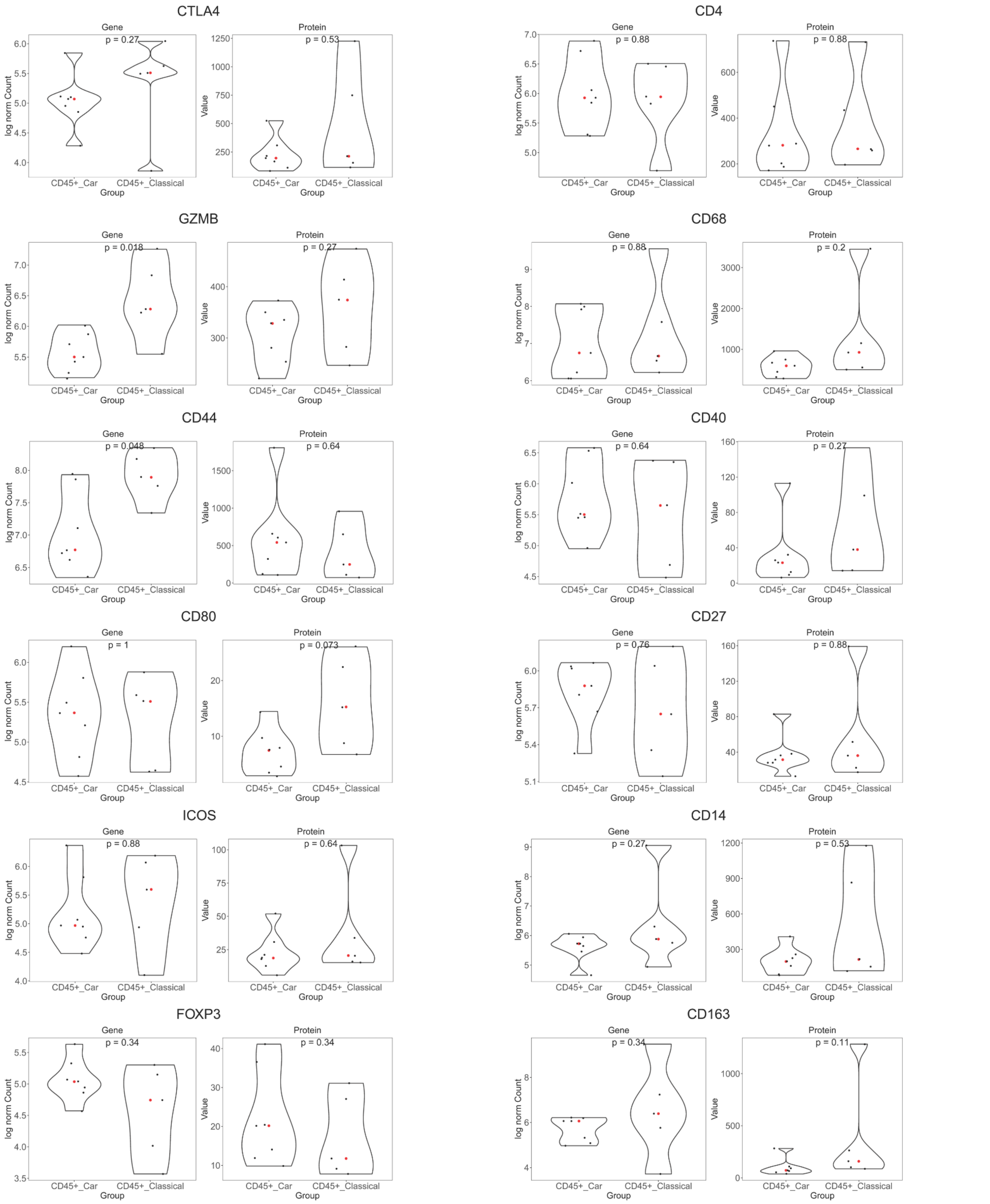

Figure S23. Gene/protein correlation for cardiac and classical NEC CD45⁺ analysis, related to Figure 3. Violin plots of gene and protein counts (normalized to nuclei). Significance determined through Wilcoxon test ($p < 0.05$) comparison of medians. Abbreviations: NEC: necrotizing enterocolitis; CTLA4: cytotoxic T-lymphocyte associated protein 4; GZMB: granzyme B; ICOS: inducible T-cell costimulator; FOXP3: forkhead box P3.

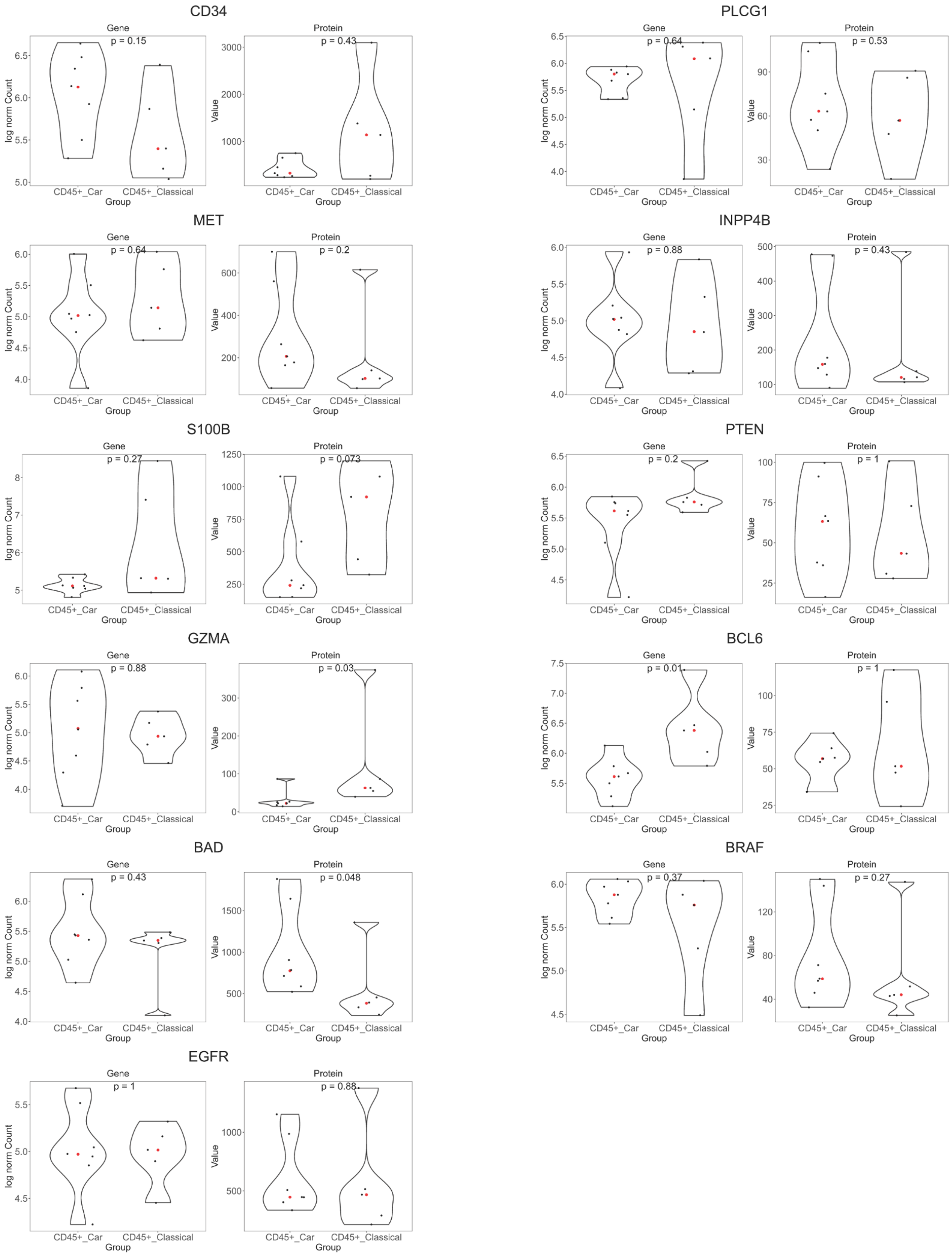

Figure S24. Gene/protein correlation for cardiac and classical NEC CD45⁺ analysis, related to Figure 3. Violin plots of gene and protein counts (normalized to nuclei). Significance determined through Wilcoxon test (*p* < 0.05) comparison of medians. Abbreviations: NEC: necrotizing enterocolitis; PLCG1: phospholipase C gamma 1; INPP4B: inositol polyphosphate-4-phosphatase type II B; S100B: S100 calcium-binding protein B; PTEN: phosphatase and tensin homolog; BCL6: B-cell lymphoma L6 transcription repressor; BAD: BCL2-associated agonist of cell death; BRAF: B-raf proto-oncogene; EGFR: epidermal growth factor receptor.

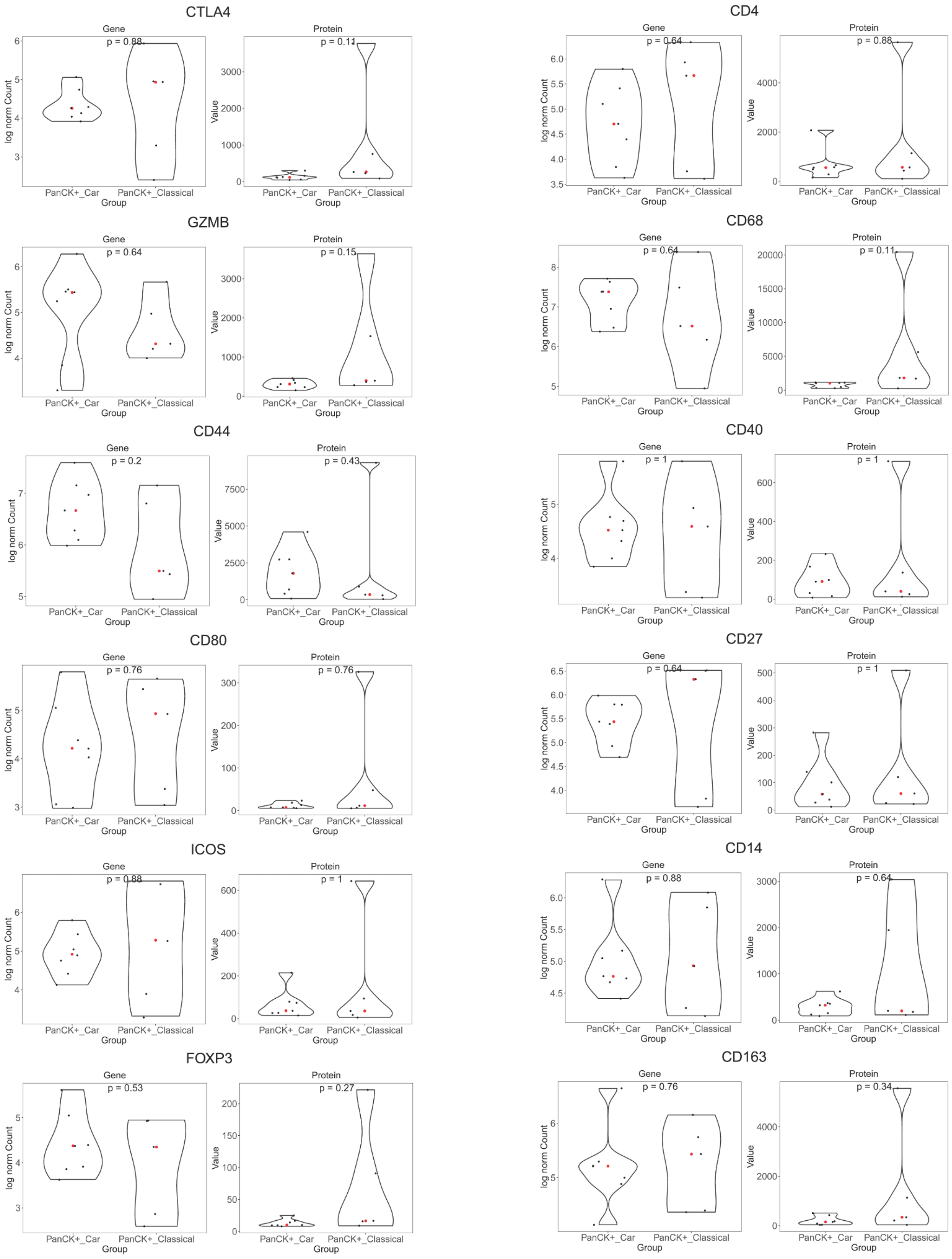

Figure S25. Gene/protein correlation for cardiac and classical NEC PanCK$^+$ analysis, related to Figure 4. Violin plots of gene and protein counts (normalized to nuclei). Significance determined through Wilcoxon test ($p < 0.05$) comparison of medians. Abbreviations: NEC: necrotizing enterocolitis; PanCK: pancytokeratin; CTLA4: cytotoxic T-lymphocyte associated protein 4; GZMB: granzyme B; ICOS: inducible T-cell costimulator; FOXP3: forkhead box P3.

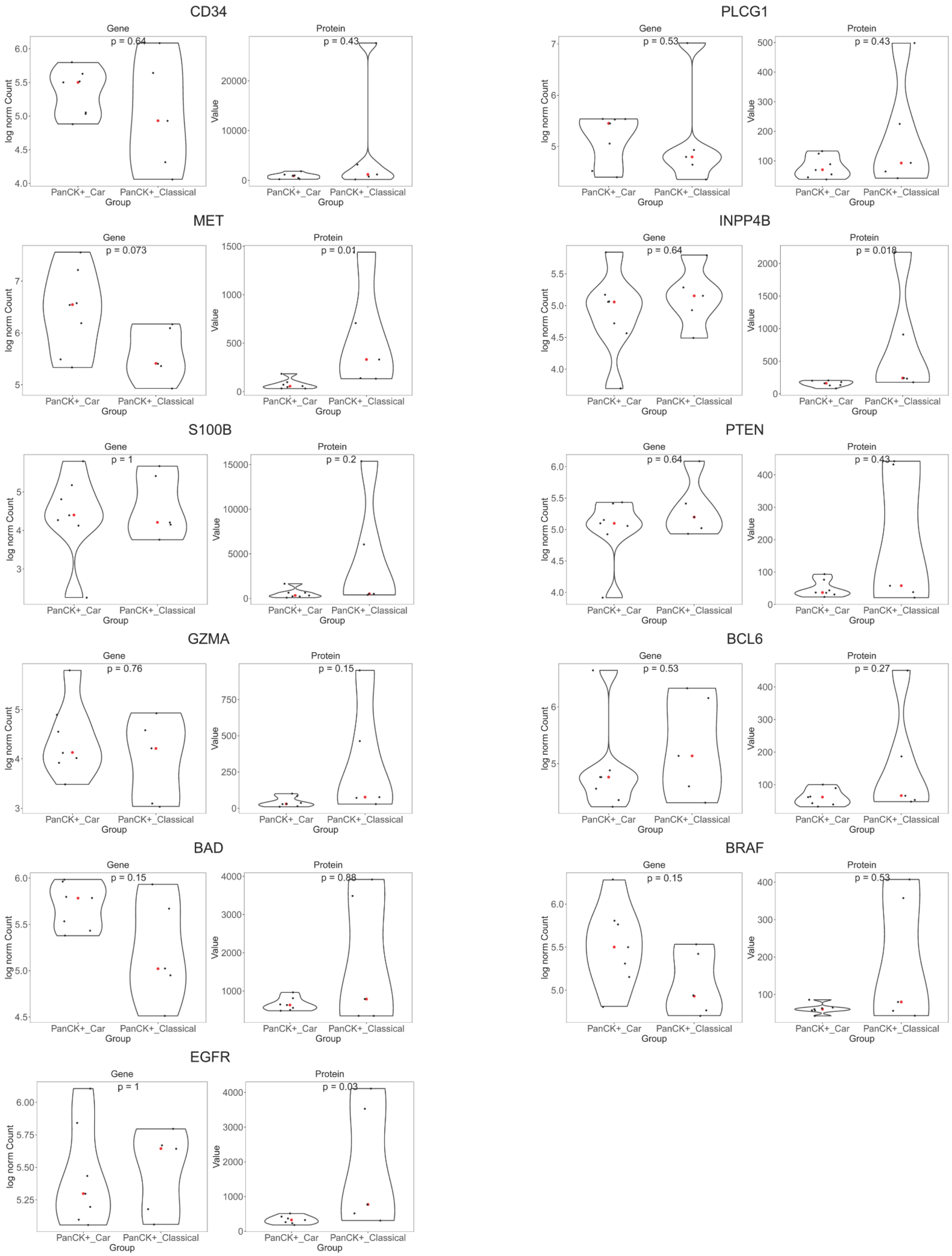

Figure S26. Gene/protein correlation for cardiac and classical NEC PanCK$^+$ analysis, related to Figure 4. Violin plots of gene and protein counts (normalized to nuclei). Significance determined through Wilcoxon test (p < 0.05) comparison of medians. Abbreviations: NEC: necrotizing enterocolitis; PanCK: pancytokeratin; PLCG1: phospholipase C gamma 1; INPP4B: inositol polyphosphate-4-phosphatase type II B; S100B: S100 calcium-binding protein B; PTEN: phosphatase and tensin homolog; BCL6: B-cell lymphoma L6 transcription repressor; BAD: BCL2-associated agonist of cell death; BRAF: B-raf proto-oncogene; EGFR: epidermal growth factor receptor.

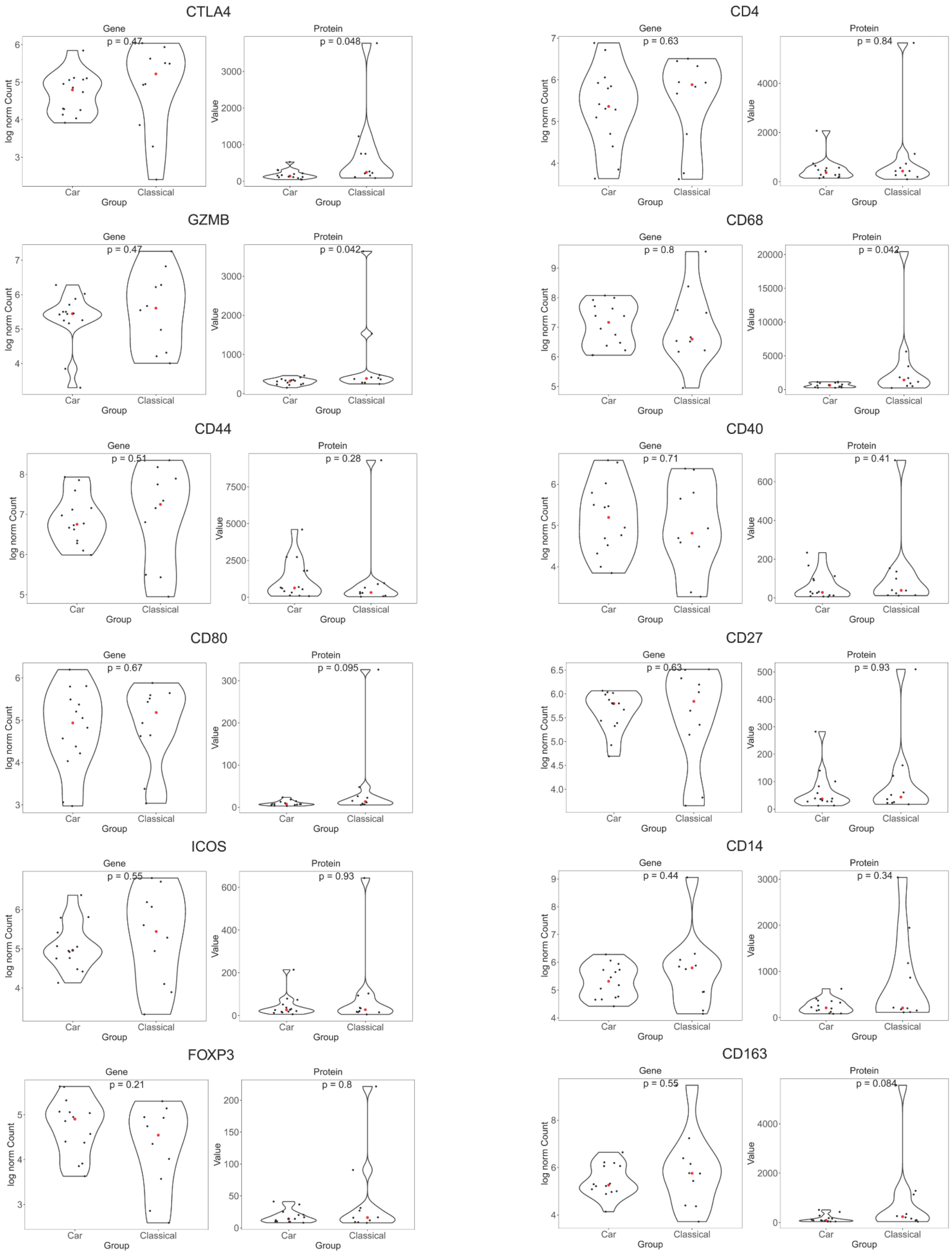

Figure S27. Gene/protein correlation for cell-type segment directionally concordant analysis, related to Figure 5. Violin plots of gene and protein counts (normalized to nuclei). Significance determined through Wilcoxon test ($p < 0.05$) comparison of medians. Abbreviations: CTLA4: cytotoxic T-lymphocyte associated protein 4; GZMB: granzyme B; ICOS: inducible T-cell costimulator; FOXP3: forkhead box P3.

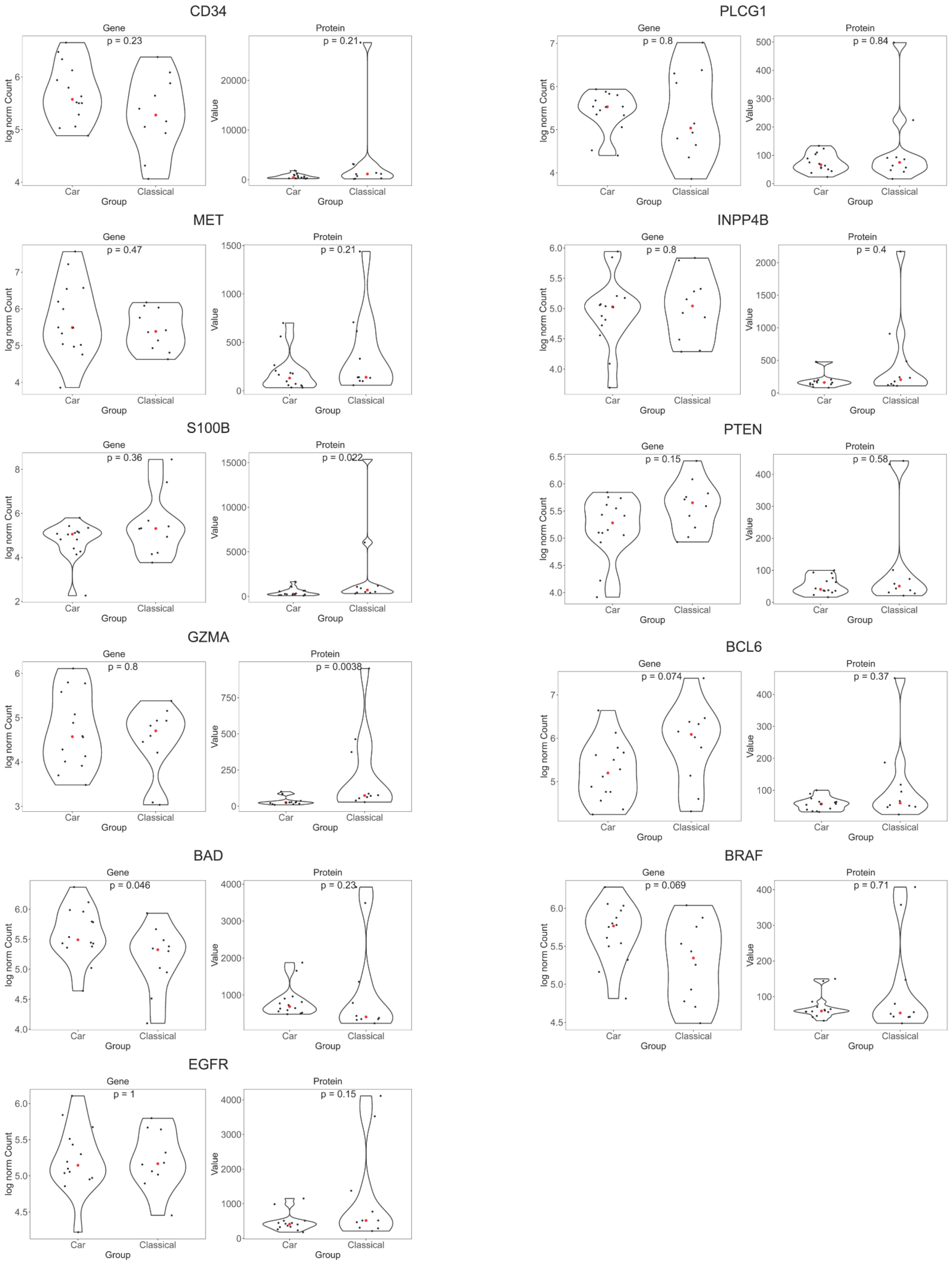

Figure S28. Gene/protein correlation for cell-type segment directionally concordant analysis, related to Figure 5. Violin plots of gene and protein counts (normalized to nuclei). Significance determined through Wilcoxon test ($p < 0.05$) comparison of medians. Abbreviations: PLCG1: phospholipase C gamma 1; INPP4B: inositol polyphosphate-4-phosphatase type II B; S100B: S100 calcium-binding protein B; PTEN: phosphatase and tensin homolog; BCL6: B-cell lymphoma L6 transcription repressor; BAD: BCL2-associated agonist of cell death; BRAF: B-raf proto-oncogene; EGFR: epidermal growth factor receptor.

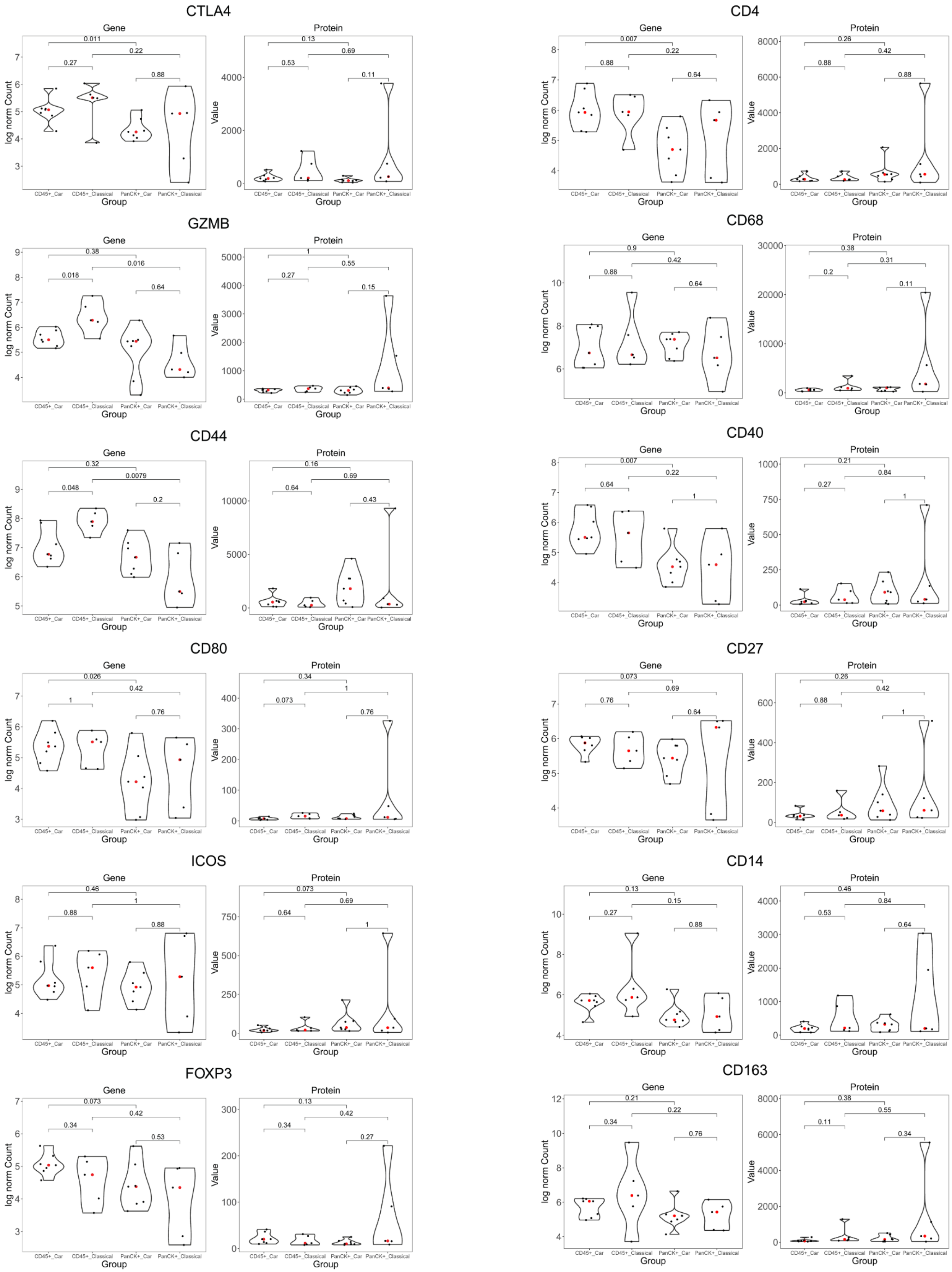

Figure S29. Gene/protein correlation for interaction gene analysis, related to Figure 6. Violin plots of gene and protein counts (normalized to nuclei). Significance determined through Wilcoxon test ($p < 0.05$) comparison of medians. Abbreviations: CTLA4: cytotoxic T-lymphocyte associated protein 4; GZMB: granzyme B; ICOS: inducible T-cell costimulator; FOXP3: forkhead box P3.

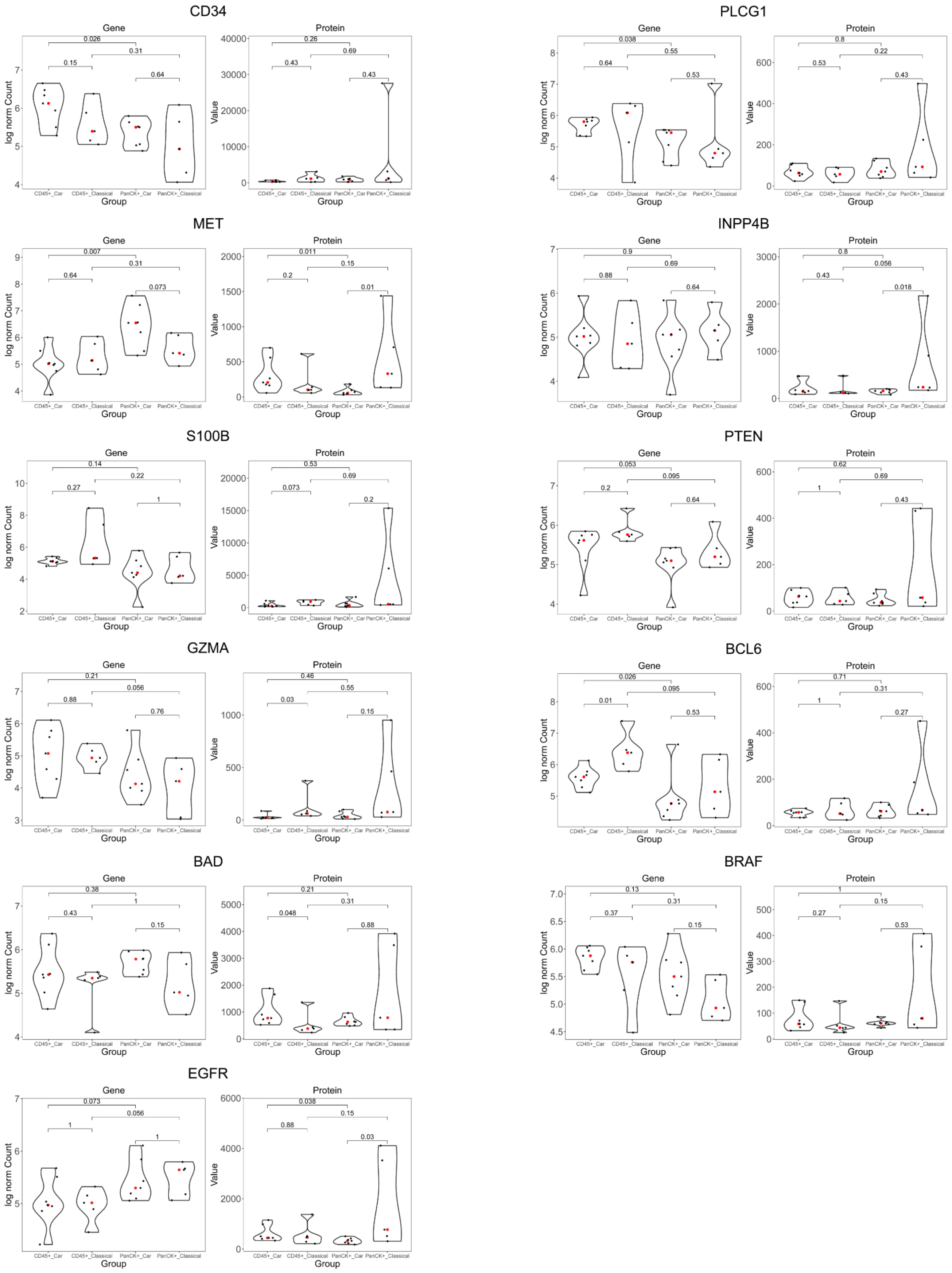

Figure S30. Gene/protein correlation for interaction gene analysis, related to Figure 6. Violin plots of gene and protein counts (normalized to nuclei). Significance determined through Wilcoxon test ($p < 0.05$) comparison of medians. Abbreviations: PLCG1: phospholipase C gamma 1; INPP4B: inositol polyphosphate-4-phosphatase type II B; S100B: S100 calcium-binding protein B; PTEN: phosphatase and tensin homolog; BCL6: B-cell lymphoma L6 transcription repressor; BAD: BCL2-associated agonist of cell death; BRAF: B-raf proto-oncogene; EGFR: epidermal growth factor receptor.

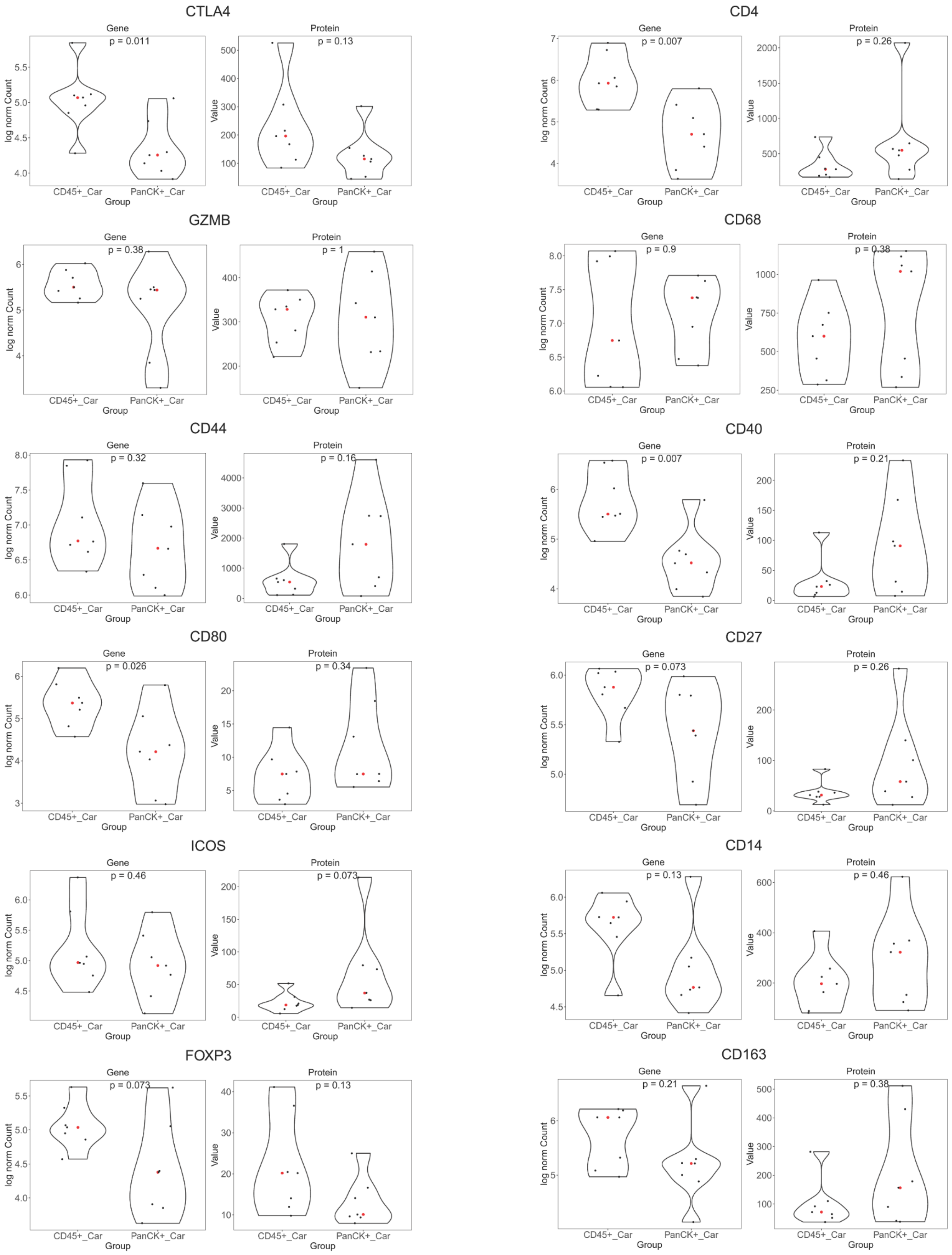

Figure S31. Gene/protein correlation for cardiac NEC CD45⁺ vs PanCK⁺ analysis, related to STAR Methods. Violin plots of gene and protein counts (normalized to nuclei). Significance determined through Wilcoxon test (p < 0.05) comparison of medians. Abbreviations: NEC: necrotizing enterocolitis; PanCK: pancytokeratin; CTLA4: cytotoxic T-lymphocyte associated protein 4; GZMB: granzyme B; ICOS: inducible T-cell costimulator; FOXP3: forkhead box P3.

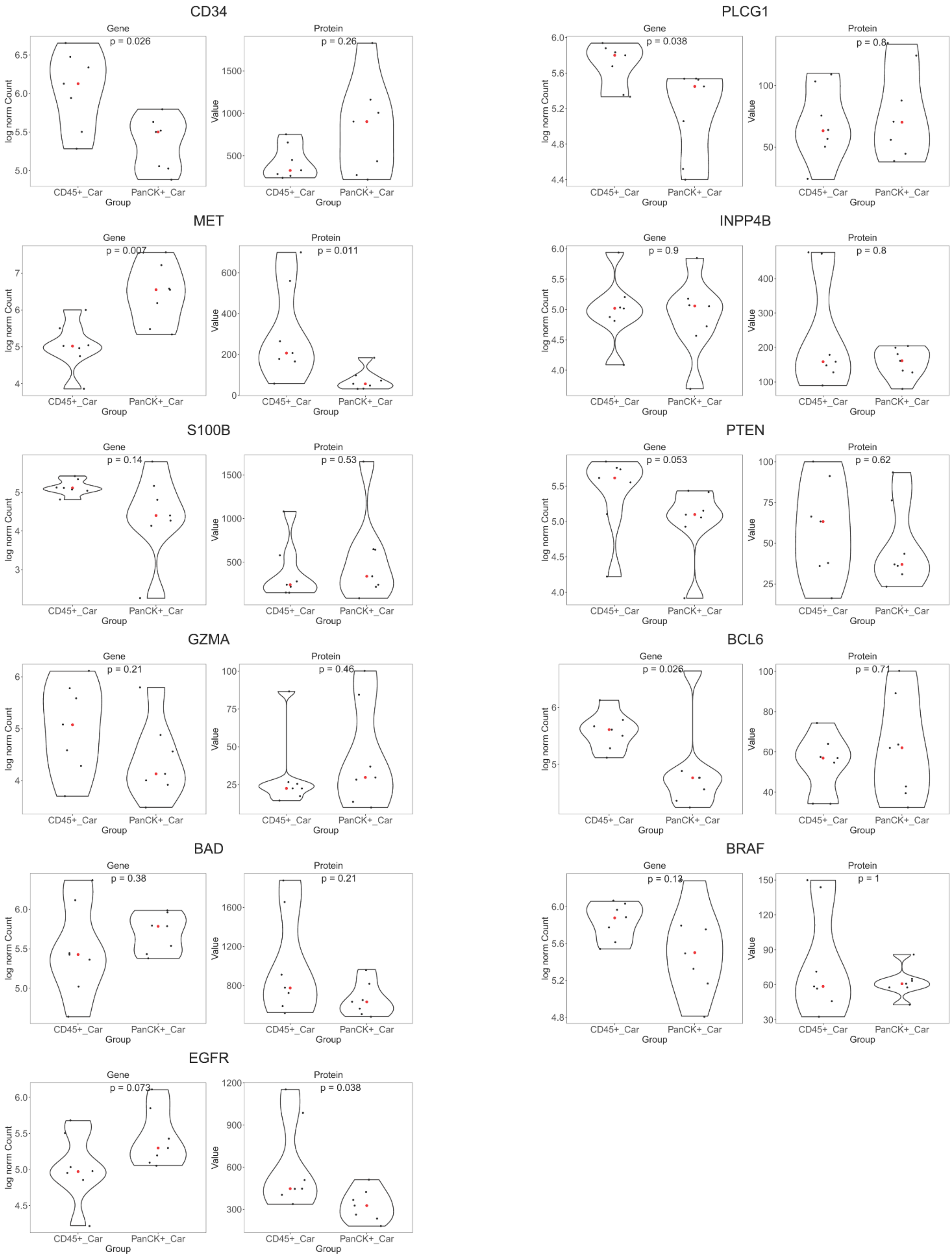

Figure S32. Gene/protein correlation for cardiac NEC CD45$^+$ vs PanCK$^+$ analysis, related to STAR Methods. Violin plots of gene and protein counts (normalized to nuclei). Significance determined through Wilcoxon test (p < 0.05) comparison of medians. Abbreviations: NEC: necrotizing enterocolitis; PanCK: pancytokeratin; PLCG1: phospholipase C gamma 1; INPP4B: inositol polyphosphate-4-phosphatase type II B; S100B: S100 calcium-binding protein B; PTEN: phosphatase and tensin homolog; BCL6: B-cell lymphoma L6 transcription repressor; BAD: BCL2-associated agonist of cell death; BRAF: B-raf proto-oncogene; EGFR: epidermal growth factor receptor.

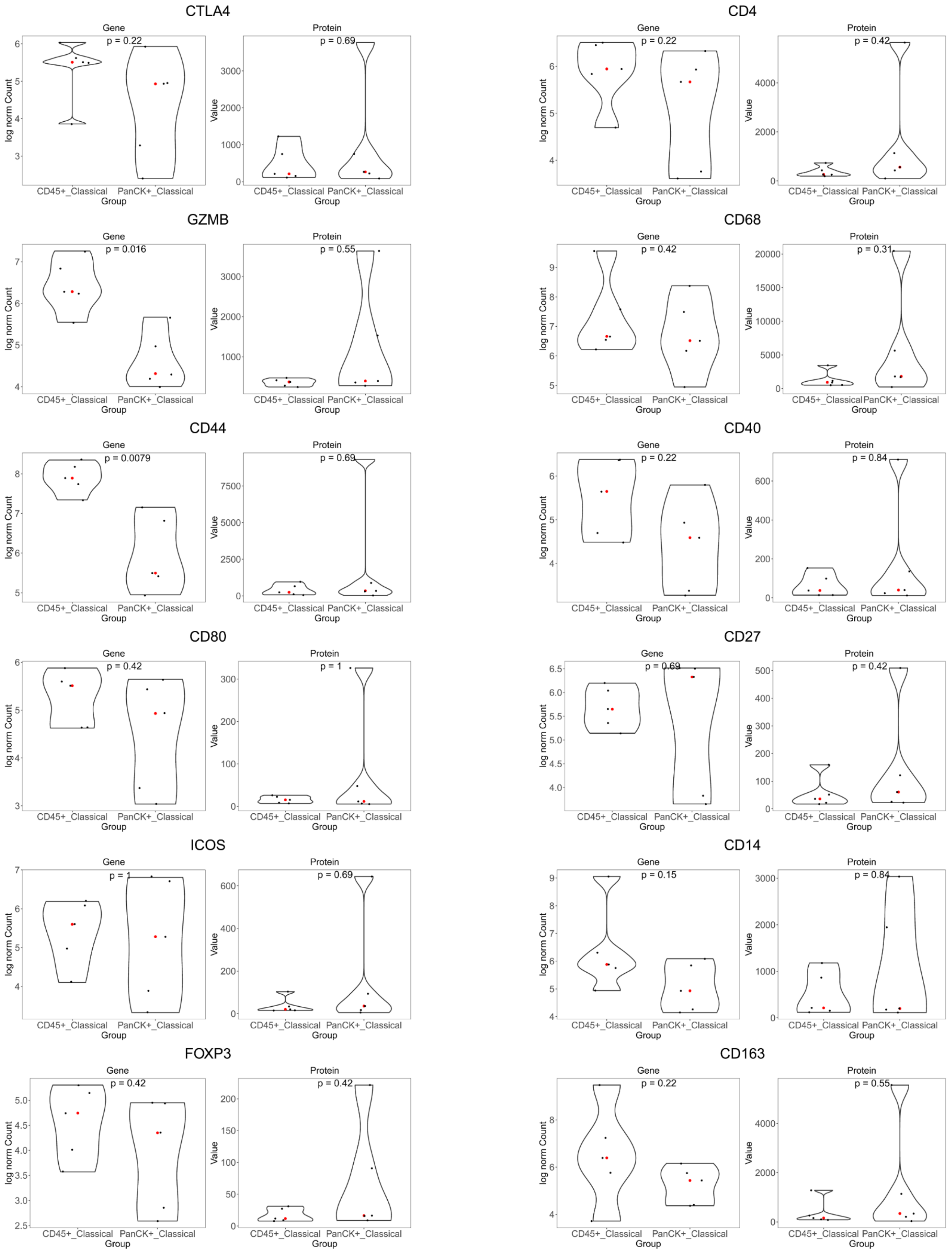

Figure S33. Gene/protein correlation for classical NEC CD45⁺ vs PanCK⁺ analysis, related to STAR Methods. Violin plots of gene and protein counts (normalized to nuclei). Significance determined through Wilcoxon test ($p < 0.05$) comparison of medians. Abbreviations: NEC: necrotizing enterocolitis; PanCK: pancytokeratin; CTLA4: cytotoxic T-lymphocyte associated protein 4; GZMB: granzyme B; ICOS: inducible T-cell costimulator; FOXP3: forkhead box P3.

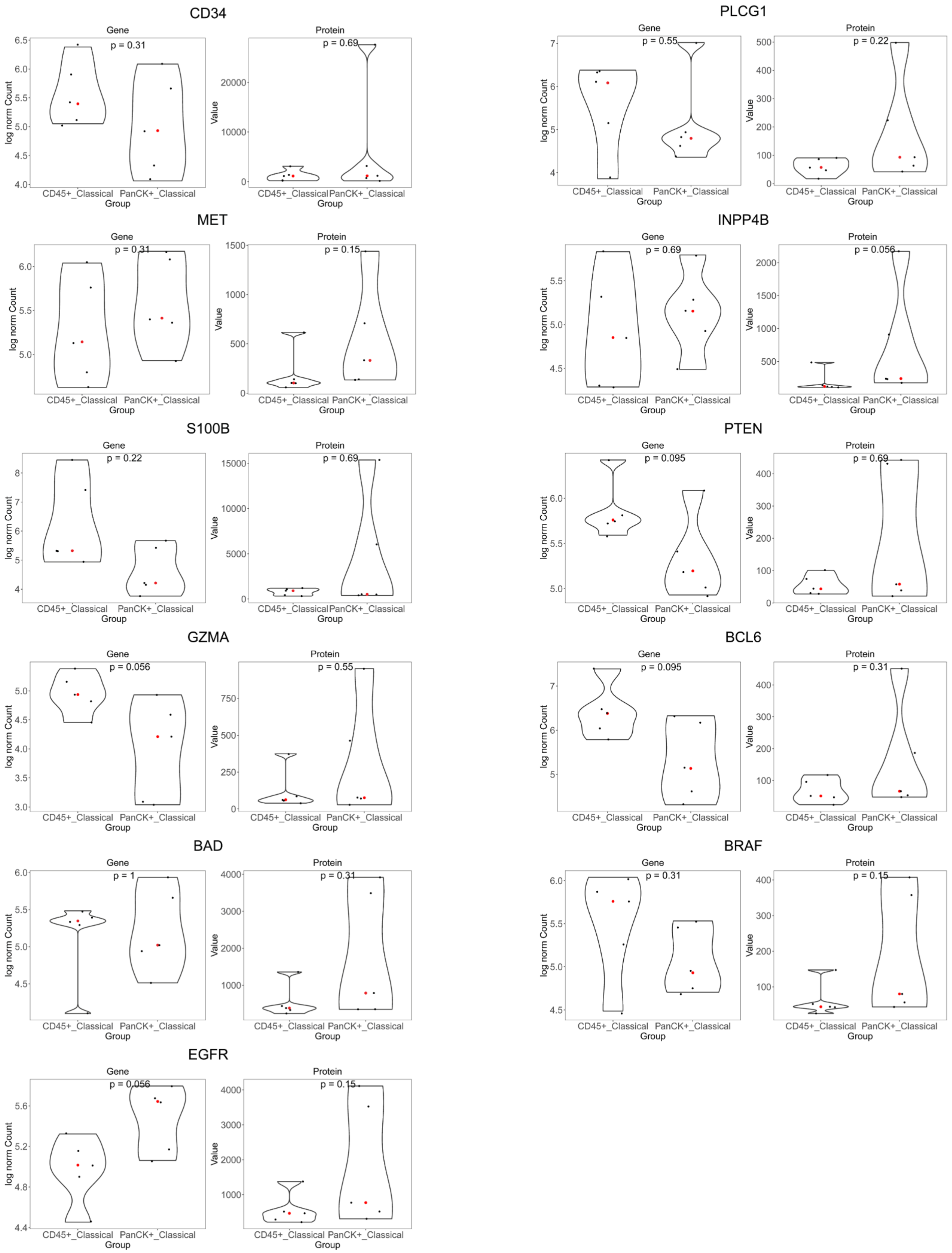

Figure S34. Gene/protein correlation for classical NEC CD45⁺ vs PanCK⁺ analysis, related to STAR Methods. Violin plots of gene and protein counts (normalized to nuclei). Significance determined through Wilcoxon test (p < 0.05) comparison of medians. Abbreviations: NEC: necrotizing enterocolitis; PanCK: pancytokeratin; PLCG1: phospholipase C gamma 1; INPP4B: inositol polyphosphate-4-phosphatase type II B; S100B: S100 calcium-binding protein B; PTEN: phosphatase and tensin homolog; BCL6: B-cell lymphoma L6 transcription repressor; BAD: BCL2-associated agonist of cell death; BRAF: B-raf proto-oncogene; EGFR: epidermal growth factor receptor.

	Patient 1	Patient 2	Patient 3	Patient 4	Patient 5	Patient 6	Patient 7	*p*
**Intestinal Disease**								
NEC Subtype	Cardiac	Cardiac	Classical	Classical	Classical	Classical	Classical	N/A
Pathology Report	Mucosal ischemic necrosis with hemorrhage	Diffuse ischemic necrosis	Transmural necrosis, ischemic necrosis with acute serositis	Transmural necrosis with marked inflammatory infiltrates/ bacterial colonization	Subacute NEC with focal perforation and marked acute/chronic inflammation	Mucosal ulceration, transmural necrosis with acute inflammation/ perforation	Multifocal perforation and bacterial colonization	N/A
**Demographics**								
Sex	Male	Male	Male	Female	Male	Male	Male	>0.999
Race	A	W	AA	W	W	W	AA	>0.999
GA (Delivery), wk	39	32	28	25 3/7	26 4/7	28 1/7	29 4/7	**0.015**
Birthweight, g	3930	2600	1350	564	857	900	1090	**0.003**
GA (Surgery), wk	39 2/7	35 5/7	32 2/7	30 3/7	30 4/7	30 2/7	34 3/7	**0.016**
**Perinatal Metrics**								
Maternal Drug Use	No	No	Yes (EtOH/ THC)	Yes (EtOH)	No	No	Yes (THC)	0.429
Mode of Delivery	Vaginal	Vaginal	C-Section	C-Section	Vaginal	C-Section	C-Section	0.143
Head Circumference, cm	34	31	30	21.5	24.5	26	27	**0.043**
Apgar Score at 5 min	7	9	7	6	7	9	9	0.739
**Comorbidities**								
CHD Diagnosis	Heterotaxy, TAPVR, AV Canal	AV canal, PDA	N/A	N/A	N/A	N/A	N/A	N/A
Other Comorbidities	N/A	N/A	Grade IV IVH, ROP, BPD	SGA	BPD, ROP	Grade II IVH, ROP, BPD	BPD, ROP, Seizures	N/A
**Interventions**								
Transfusions <48 h before Diagnosis	No	Yes	Yes, (24 h prior)	Yes (24 h prior)	No	No	Yes (24 h prior)	>0.999
Respiratory Support, Pre-Diagnosis	RA	NC (1.5 L/min)	NC (1 L/min, 40% O_2)	NC (2 L/min, 40% O_2)	NIV	RA	RA	N/A
Respiratory Support, Post-Diagnosis	Intubated	Intubated	Intubated	Intubated	Intubated	Intubated	Intubated	1
**NICU Metrics**								
Feed Type/Volume	NPO	Formula/ Full Feeds	DM/HMF/ Full Feeds	MOM/HMF/ Full Feeds	MOM/HMF/ Full Feeds	MOM/HMF/ Full Feeds	Formula/ Full Feeds	N/A
Hb/Hct, g/dL/%	15.9/ 45.7	15.5/ 44.8	9.1/ 25.4	9.8/ 27.7	14.5/ 43.1	11/ 31.8	9.4/ 27.5	**0.031/ 0.045**
WBC (x10^3/mm^3)	14.32	3.18	13.65	2.52	2.18	36.2	1.7	0.836
Platelet Nadir w/in 48 h of Diagnosis, per µL	221,000	203,000	32,000	84,000	83,000	39,000	35,000	**0.001**
Lymphocytes, %	20	43	28	40	52	22	77	0.512
Neutrophils, %	71	28	57	47	30	48	7	0.559
Monocytes, %	8	16	3	6	9	9	11	0.222
**Surgical Metrics**								
Resection Location	Ileum/ Colon	Ileum	Ileum/ Colon	Jejunum/ Ileum	Ileum/ICV/ Colon	Ileum/Colon	Ileum	N/A
Length Resected, cm	15 ileum, 5 colon	22	10	40	11 ileum, 2 colon/ICV	10 ileum, 4 colon	22.5	N/A
NEC Timing Relative to CHD Surgery	Before	Before	N/A	N/A	N/A	N/A	N/A	N/A
**Clinical Outcome**								

Survival (cause of death)	No (sepsis)	Yes	Yes	No (NEC)	Yes	Yes	Yes	>0.999
GI Outcomes		G-Tube			G-Tube, SBS (home TPN)		G-Tube	N/A

Table S1. Patient clinical and demographic characteristics, related to STAR Methods and Figure 1. Significance (bolded, $p < 0.05$) was determined by unpaired, two-tailed T-tests or Fisher's exact tests, as appropriate. Abbreviations: NEC: necrotizing enterocolitis; A: Asian; W: White; AA: African American; GA: gestational age; EtOH: ethanol; THC: tetrahydrocannabinol; C-Section: Cesarean Section; CHD: congenital heart disease; TAPVR: total anomalous pulmonary venous return; AV: atrioventricular; PDA: patent ductus arteriosus; IVH: intraventricular hemorrhage; ROP: retinopathy of prematurity; BPD: bronchopulmonary dysplasia; SGA: small-for-gestational age; RA: room air; NC: nasal cannula; NIV: non-invasive ventilation; NPO: nil per os; DM: donor milk; HMF: human milk fortifier; MOM: mother's own milk; Hb: hemoglobin; Hct: hematocrit; WBC: white blood cells; ICV: ileocecal valve; G-Tube: gastrostomy tube; SBS: short bowel syndrome; TPN: total parenteral nutrition.

Comparison	Number of DEGs	FDR-adjusted *p*-value
Cardiac Immune vs. Classical Immune	295	0.15
Cardiac Epithelium vs. Classical Epithelium	587	0.01
Classical Epithelium vs. Classical Immune	898	0.01
Cardiac Epithelium vs. Cardiac Immune	396	0.05
Cardiac vs. Classical Cell-Type Directionally Concordant Genes	411	0.15
Cardiac vs. Classical Interaction Genes	449	0.05

Table S2. Statistical thresholds (FDR-adjusted *p*-values) for determination of DEGs, related to STAR Methods. Abbreviations: DEGs: differentially expressed genes; FDR: false discovery rate.

Immune Cell Profiling	Immune Activation Status	Immune Cell Typing	PI3K/AKT Signaling	Pan Tumor	Cell Death	MAPK Signaling
GZMB	CD127	FOXP3	phospho-GSK3A (S21)/phospho-GSK3B (S9)	PTEN	BAD	phospho-JNK (T183/Y185)
Ms IgG1	CD25	CD14	INPP4B	Bcl-2	BCL6	phospho-MEK1 (S217/S221)
Ms IgG2a	CD27	CD163	MET	EpCAM	BCLXL	phospho-p38 MAPK (T180/Y182)
Rb IgG	CD40	CD34	Pan-AKT	ERα	BIM	BRAF
GAPDH	CD44	CD45RO	Phospho-AKT1 (S473)	Her2	CD95/Fas	EGFR
histone H3	CD80	CD66b	Phospho-GSK3B (S9)	MART1	cleaved caspase-9	p44/42 MAPK (ERK1/2)
S6	ICOS	FAPα	Phospho-PRAS40 (T246)	NY-ESO-1	GZMA	pan-RAS
B2M	PD-L2		Phospho-tuberin (T1462)	PR	neurofibromin	phospho-c-RAF (S338)
CD11c			PLCG1	S100B	p53	phospho-p44/42 MAPK ERK1/2 (T202/Y204)
CD20					PARP	phospho-p90 RSK (T359/S363)
CD3						
CD4						
CD45						
CD56						
CD68						
CD8						
CTLA4						
FN						
HLA-DR						
Ki-67						
PanCK						
PD-1						
PD-L1						
SMA						

Table S3. Proteins targeted with NanoString™ DSP protein panels, related to STAR Methods. Abbreviations: DSP: Digital Spatial Profiling; PI3K: phosphatidylinositol-3-kinase; MAPK: mitogen-activated protein kinase; GZMB: granzyme B; FOXP3: forkhead box P3; GSK3A: glycogen synthase kinase 3 alpha; GSK3B: GSK3 beta; PTEN: phosphatase and tensin homolog; BAD: BCL2-associated agonist of cell death; JNK: cJun/N-terminal kinase; Ms: mouse; INPP4B: inositol polyphosphate-4-phosphatase type II B; Bcl-2: B-cell lymphoma 2 apoptosis regulator; BCL6: B-cell lymphoma L6 transcription repressor; MEK1: mitogen-activated protein kinase kinase 1; EpCAM: epithelial cell adhesion molecule; BCLXL: B-cell lymphoma extra-large; Rb: rabbit; Erα: estrogen receptor alpha; BIM: BCL2 interacting mediator of cell death; GAPDH: glyceraldehyde-3-phosphate dehydrogenase; Her2: receptor tyrosine-protein kinase erbB-2; EGFR: epidermal growth factor receptor; MART1: protein melan-A; ERK1/2: extracellular signal-regulated kinase 1/2; ICOS: inducible T-cell co-stimulator; FAPα: fibroblast activation protein alpha; PRAS40: proline-rich Akt substrate of 40 kDa; NY-ESO-1: New York esophageal squamous cell carcinoma-1; B2M: beta 2-microglobulin; PD-L2: programmed cell death 1 ligand 2; PR: progesterone receptor; PLCG1: phospholipase C gamma 1; S100B: S100 calcium-binding protein B; PARP: poly(ADP-ribose) polymerase 1; RSK: ribosomal S6 kinase; CTLA4: cytotoxic T-lymphocyte associated protein 4; FN: fibronectin; HLA-DR: human leukocyte antigen, DR isotype; PanCK: pan-cytokeratin; PD-1: programmed cell death 1; PD-L1: programmed cell death 1 ligand 1; SMA: alpha-smooth muscle actin.

Viral Protein Interaction with Cytokine and Cytokine Receptor	Chemokine Signaling Pathway	Small Cell Lung Cancer	FCGR-Dependent Phagocytosis	TNFR2 Non-Canonical NF-kB Pathway	Proteasome	Focal Adhesion	Host Interaction of HIV Factors	IL-10 Signaling
CD45$^+$ Classical vs. Cardiac NEC	CD45$^+$ Classical vs. Cardiac NEC	PanCK$^+$ Classical vs. Cardiac NEC	Classical vs. Cardiac NEC (directionally concordant)	Interaction Gene Analysis	Interaction Gene Analysis	Interaction Gene Analysis	Interaction Gene Analysis	Interaction Gene Analysis
CCL15<br>*CCL21*<br>*CCL23*<br>*CX3CR1*<br>*CXCL6*<br>*CXCR3*<br>*PF4V1*<br>IL2RA (CD25)	*ADCY5*<br>*CCL15*<br>*CCL21*<br>*CCL23*<br>*CX3CR1*<br>*CXCL6*<br>*CXCR6*<br>*GNAI3*<br>*GRK6*<br>*PF4V1*<br>BAD<br>BRAF<br>PLCG1	*BAX1*<br>*BCL2L1*<br>*CCND1*<br>*COL4A2*<br>*E2F1*<br>*ITGA6*<br>*LAMA3*<br>*LAMA4*<br>*MAX*<br>*RXRB*<br>*ZBTB17*<br>BCL2<br>PTEN	*CD247*<br>*ELMO2*<br>*IGHG2*<br>*IGHG3*<br>*MYH2*<br>*NCKAP1L*<br>*VAV1*<br>PLCG1	*MAP3K14*<br>*PSMA7*<br>*PSMB5*<br>*PSMD12*<br>*PSMF1*<br>*SEM1*<br>*TNFRSF12A*<br>CD40	*ADRM1*<br>*PSMA7*<br>*PSMB5*<br>*PSMD12*<br>*PSMF1*<br>*SEM1*<br>PSMC4	*CCND1*<br>*CCND2*<br>*COL4A2*<br>*COL9A2*<br>*COMP*<br>*FLNA*<br>*ITGA6*<br>*LAMA3*<br>*LAMA4*<br>*MAP2K1*<br>*MET*<br>*PGF*<br>*PP1R12C*<br>*RAC3*<br>*THBS3*<br>BAD<br>BCL2<br>BRAF<br>EGFR<br>MET<br>PTEN	*CD8B*<br>*PSMB5*<br>*PSMF1*<br>*PSMD12*<br>*PSMA7*<br>*NUP214*<br>*RANGAP1*<br>*SEM1*<br>*ELMO1*<br>*BTRC*<br>PSMC4	*CCL20*<br>*CCR1*<br>*CD80*<br>*IL10RA*<br>*IL1R2*<br>CD80

Table S4. Results of integrated gene/protein network analyses, related to Figures 3-6. DEGs (italicized) and proteins (non-italicized) overlapping with indicated differentially enriched pathways in classical compared with cardiac NEC. Second row indicates analysis in which pathways were differentially enriched. Abbreviations: FCGR: Fc gamma receptor; TNFR2: tumor necrosis factor receptor 2; NF-kB: nuclear factor kappa beta; HIV: human immunodeficiency virus; IL-10: interleukin 10; *CCL15*: CC motif chemokine ligand 15; *ADCY5*: adenylate cyclase 5; *BAX1*: BCL2 associated X, apoptosis regulator 1; *MAP3K14*: mitogen-activated protein kinase kinase kinase 14; *ADRM1*: adhesion regulating molecule 1; CCND1: cyclin D1; *BCL2L1*: BCL2 like 1; *ELMO2*: engulfment and cell motility 2; *PSMA7*: proteasome 20S subunit alpha 7; *CCR1*: CC motif chemokine receptor 1; *IGHG2*: immunoglobulin heavy constant gamma 2; *COL4A2*: collagen type IV alpha 2 chain; *CX3CR1*: C-X3-C motif chemokine receptor 1; *IL10RA*: interleukin 10 receptor subunit alpha; *CXCL6*: CXC motif chemokine ligand 6; *E2F1*: E2F transcription factor 1; *MYH2*: myosin-2; *COMP*: cartilage oligomeric matrix protein; *IL1R2*: interleukin 1 receptor type 2; *CXCR3*: CXC motif chemokine receptor 3; *ITGA6*: integrin subunit alpha 6; *NCKAP1L*: NCK associated protein 1 like; *SEM1*: SEM1 26S proteasome subunit; *FLNA*: filamin A; *NUP214*: nucleoporin 214; *PF4V1*: platelet factor 4 variant 1; *LAMA3*: laminin subunit alpha-3; *VAV1*: vav guanine nucleotide exchange factor 1; *TNFRSF12A*: TNF receptor superfamily member 12A; *RANGAP1*: Ran GTPase activating protein 1; *GNAI3*: G protein subunit alpha i3; PLCG1: phospholipase C gamma 1; *GRK6*: G protein-coupled receptor kinase 6; *MAX*: MYC associated factor X; *RXRB*: retinoid X receptor beta; *BTRC*: beta-transducin repeat containing E3 ubiquitin protein ligase; BAD: BCL2 associated agonist of cell death; *ZBTB17*: zinc finger and BTB domain containing 17; *MET*: mesenchymal epithelial transition; BRAF: B-raf proto-oncogene; BCL2: B-cell lymphoma 2; *PGF*: placental growth factor; PTEN: phosphatase and tensin homolog; *PP1R12C*: protein phosphatase 1 regulatory subunit 12C; *RAC3*: rac family small GTPase 3; *THBS3*: thrombospondin 3; EGFR: epidermal growth factor receptor; DEGs: differentially expressed genes.
